# Supplementary figures and images for: A model of early-life interactions between the gut microbiome and adaptive immunity provides insights into the ontogeny of immune tolerance
Source: PLoS Biol. 2025 Aug 14;23(8):e3003263. doi: 10.1371/journal.pbio.3003263 (PMC12352683; doi:10.1371/journal.pbio.3003263)

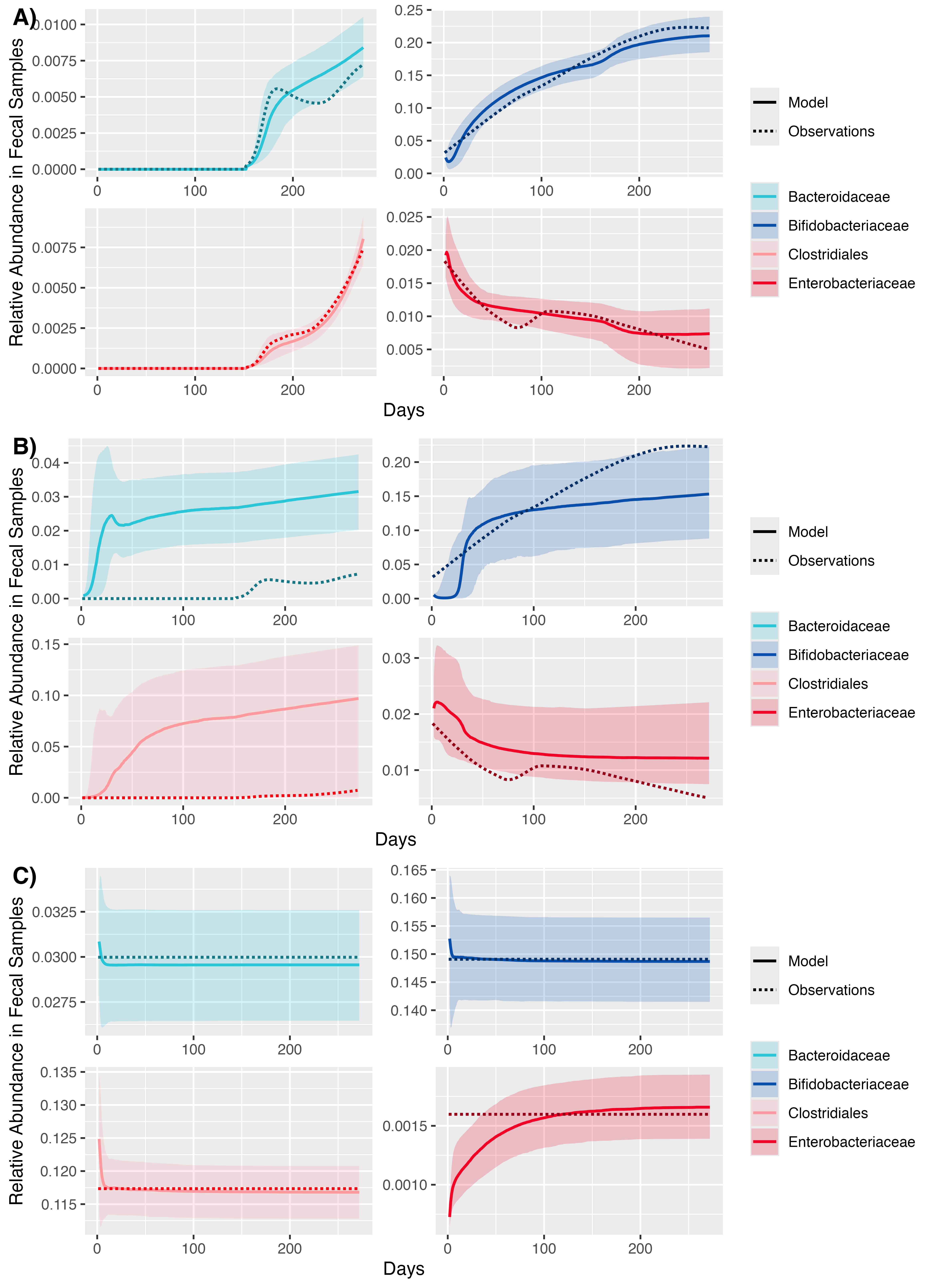

Supplement: S1 Fig — Two chains are used with 500 and 1,000 for warm-up and total iterations, respectively. Twenty-four of 2000 (2.4%) transitions ended with a divergence. (A) Relative abundance estimate results for the maternal phase, for 154 days of exclusive breastfeeding (EBF) and 308 days of mixed feeding (MF). (B) Relative abundance estimate results for the maternal phase, for 308 days of MF with no EBF. (C) Relative abundance estimate results for the steady phase, for 154 days of EBF (EBF) and 308 days of MF. Shaded areas represent the 95% confidence intervals. The data underlying this figure can be found in https://doi.org/10.5281/zenodo.15629746. (TIF) [file pbio.3003263.s001.tif]

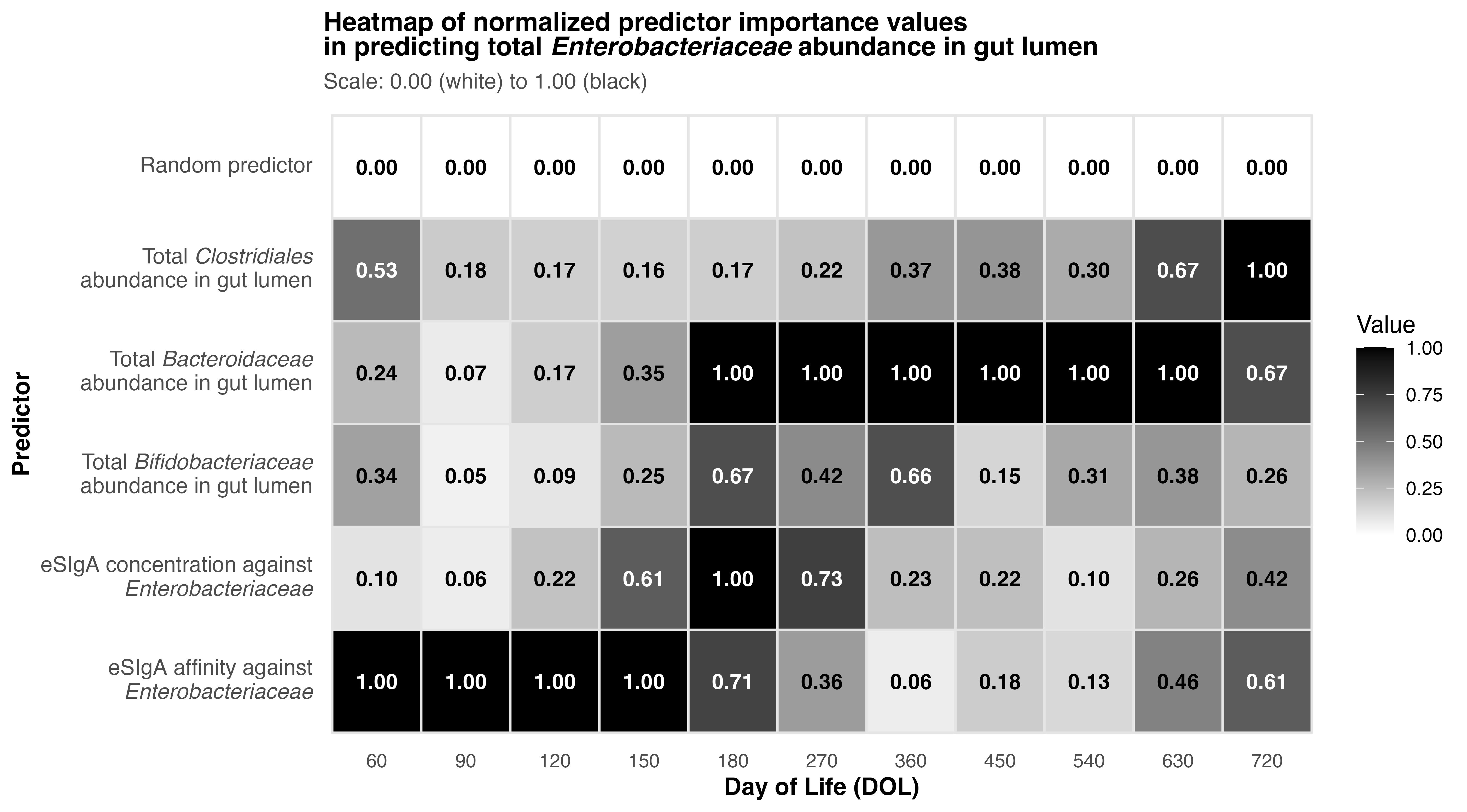

Supplement: S2 Fig — Each cell shows the normalized importance of a predictor in explaining total fecal abundance of Enterobacteriaceae at a given time point (in days), as determined by conditional permutation importance in a random‑forest model. Predictors include eSIgA affinity and concentration against Enterobacteriaceae, and total abundance of Bifidobacteriaceae, Bacteroidaceae, and Clostridiales in the gut lumen. A random predictor was included as a negative control. Predictor importance values were normalized within each time point between 0 and 1. The most influential variable in any column is black (value = 1.00) and progressively lighter shades indicate lower relative importance. Numeric values are overlaid for clarity. Starting from month 6 (DOL 180), endogenous immune responses against symbiotic commensals combined with ecological competition become the primary regulators of Enterobacteriaceae population, exerting stronger selection pressure than the endogenous SIgA (eSIgA) responses to Enterobacteriaceae itself, as seen from the decreasing importance of both the affinity and the concentration of eSIgA against Enterobacteriaceae. By DOL 720, importance values are relatively evenly distributed across predictors, consistent with the similarity in predictive power between total and SIgA-bound Enterobacteriaceae in Fig 3E. The data underlying this figure can be found in https://doi.org/10.5281/zenodo.15629746. (TIF) [file pbio.3003263.s002.tif]

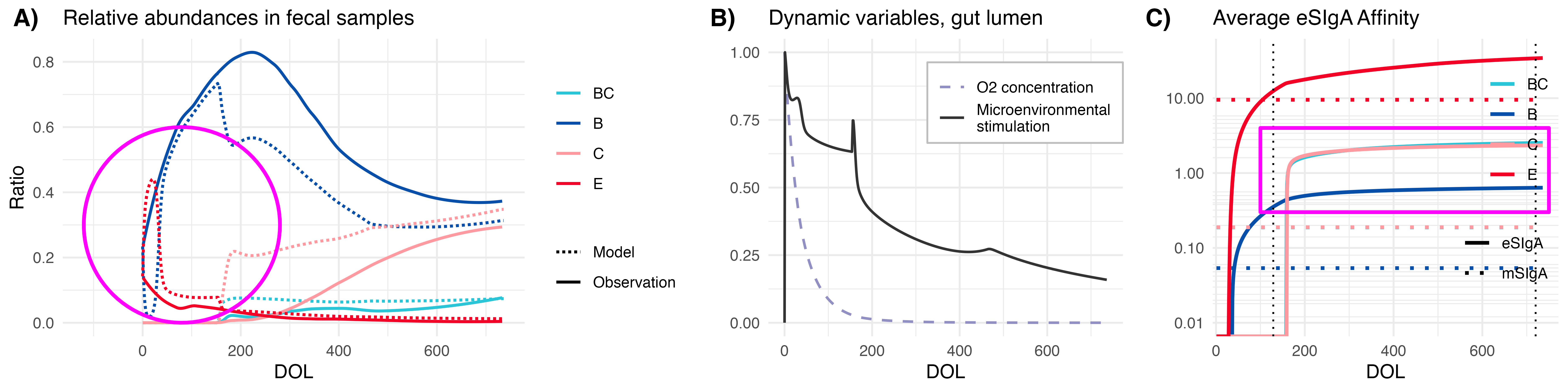

Supplement: S3 Fig — Simulation of an early-life infection scenario by giving a selective advantage to the Escherichia-Shigella genus (compartment E) relative to Bifidobacteriaceae (compartment B) by reducing the human milk oligosaccharides (HMOs) to 25% of their normal concentration in breastmilk while keeping all other parameters constant (maternal SIgA levels and affinities, breastfeeding duration, and mixed feeding periods). Panel A) demonstrates the early life dysbiosis characterized by the overgrowth of E compared to data (magenta circle), which results in higher levels of sustained inflammation during early life (panel B)), leading to a more hyperreactive response against symbiotic commensals (magenta rectangle, panel C)). The data underlying this figure can be found in https://doi.org/10.5281/zenodo.15629746. (TIF) [file pbio.3003263.s003.tif]

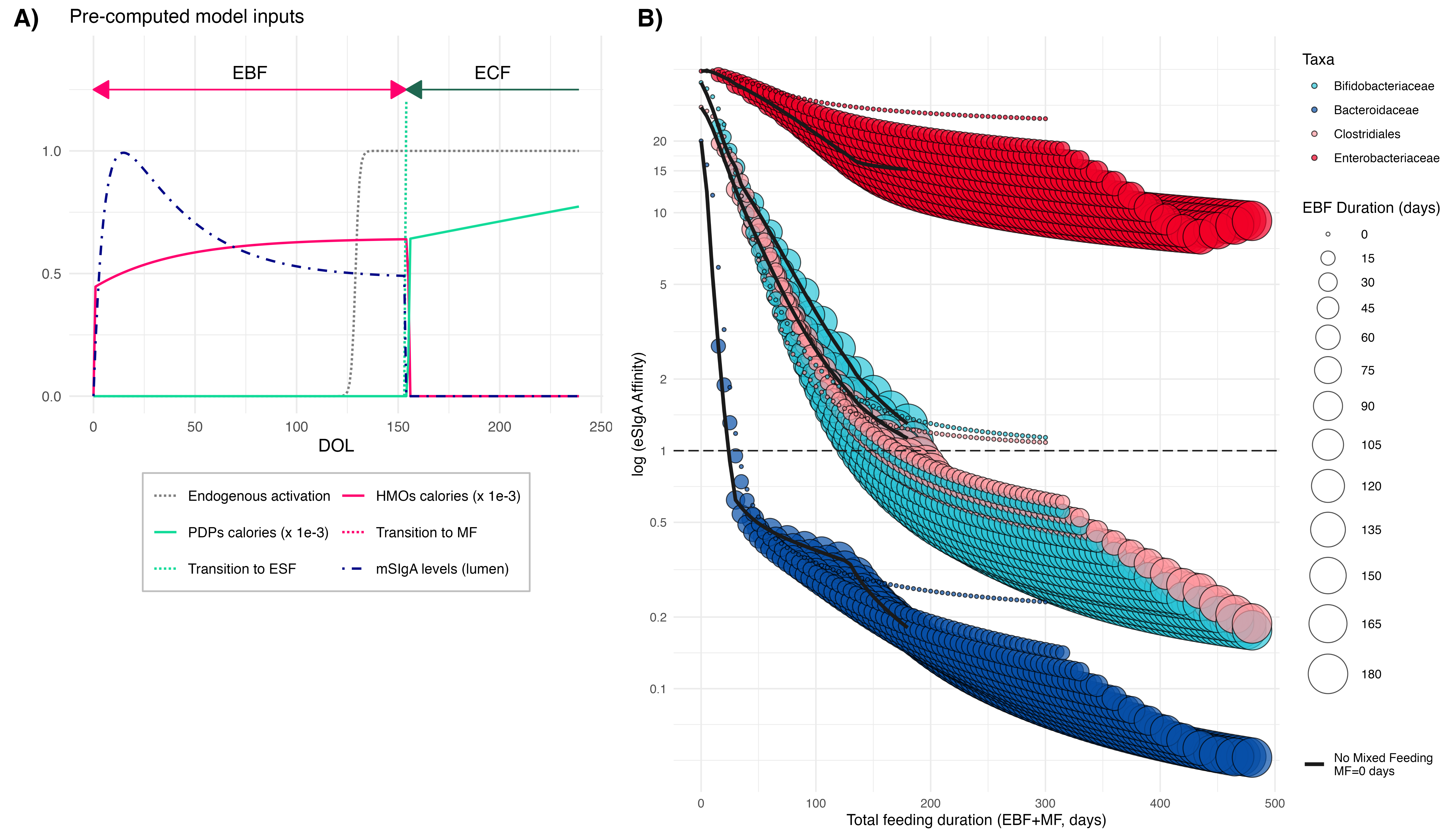

Supplement: S4 Fig — (A) Model inputs demonstrating a sharp transition from exclusive breastfeeding (EBF) to exclusive complementary feeding (ECF), with no MF period in between, including normalized maternal secretory immunoglobulin A (mSIgA) concentration, human milk oligosaccharide (HMOs) and plant-derived polysaccharides (PDPs) calorie inputs, and timing of the endogenous immune system activation over time. EBF: exclusive breastfeeding; MF: mixed feeding; ECF: exclusive complementary feeding. (B) log (eSIgA Affinity) values at steady state for different combinations of EBF and MF durations, where the solid black line demonstrates the case of no MF followed by EBF. DOL: Day of life; E: Enterobacteriaceae; B: Bifidobacteriaceae; BC: Bacteroidaceae; C: Clostridiales. The data underlying this figure can be found in https://doi.org/10.5281/zenodo.15629746. (TIF) [file pbio.3003263.s004.tif]

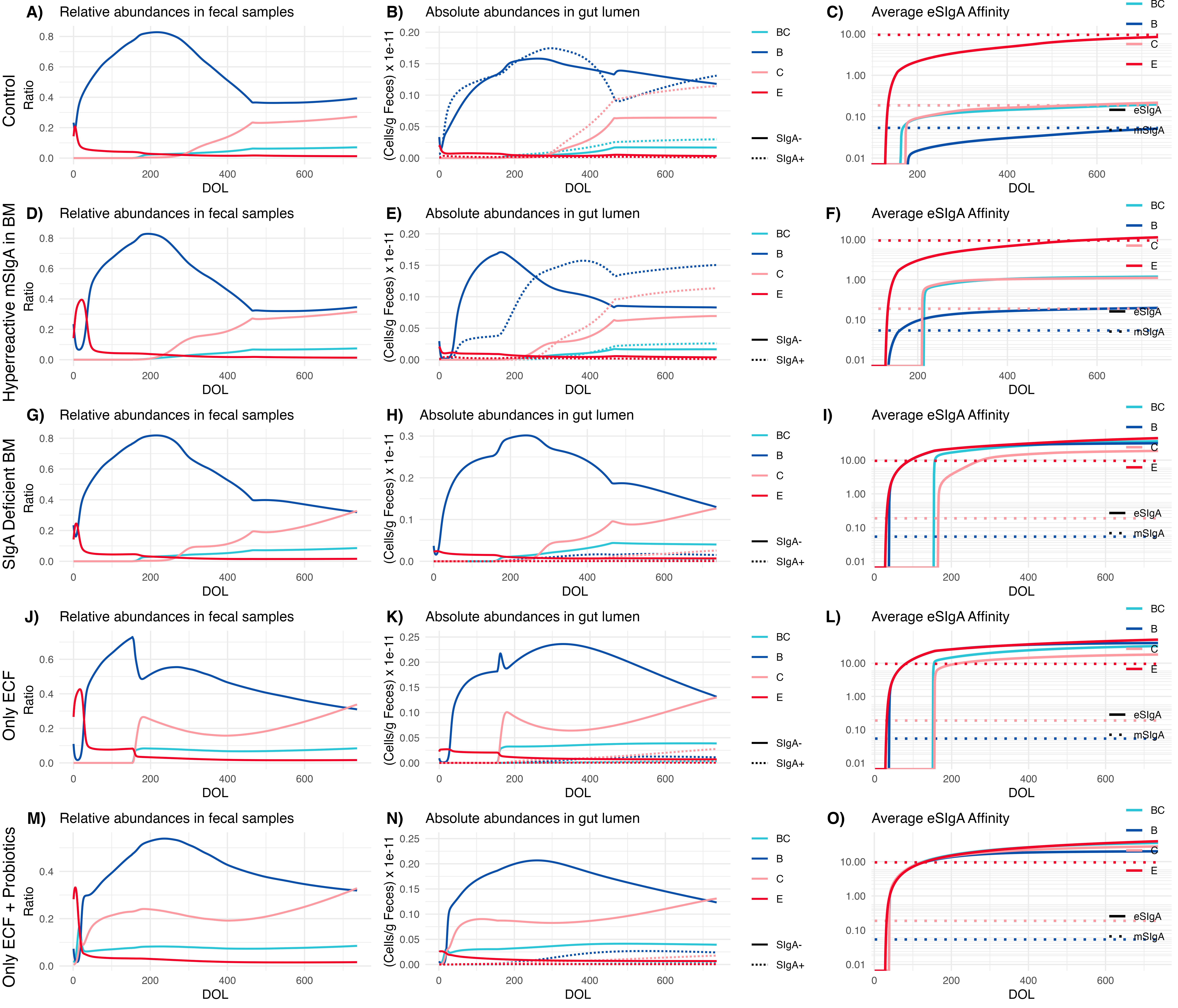

Supplement: S5 Fig — Relative abundances in fecal samples, absolute abundances in the gut lumen, and temporal progression of average endogenous SIgA (eSIgA) affinities for (A)–(C) control, (D)–(F) hyperreactive mSIgA in breastmilk (BM), (G)–(I) SIgA deficient BM, (J)–(L) only exclusive complementary feeding (ECF), and (M)–(O) ECF with probiotic (Bacteroidaceae and Clostridiales) supplementation. DOL: Day of life; E: Enterobacteriaceae; B: Bifidobacteriaceae; BC: Bacteroidaceae; C: Clostridiales. The data underlying this figure can be found in https://doi.org/10.5281/zenodo.15629746. (TIF) [file pbio.3003263.s005.tif]

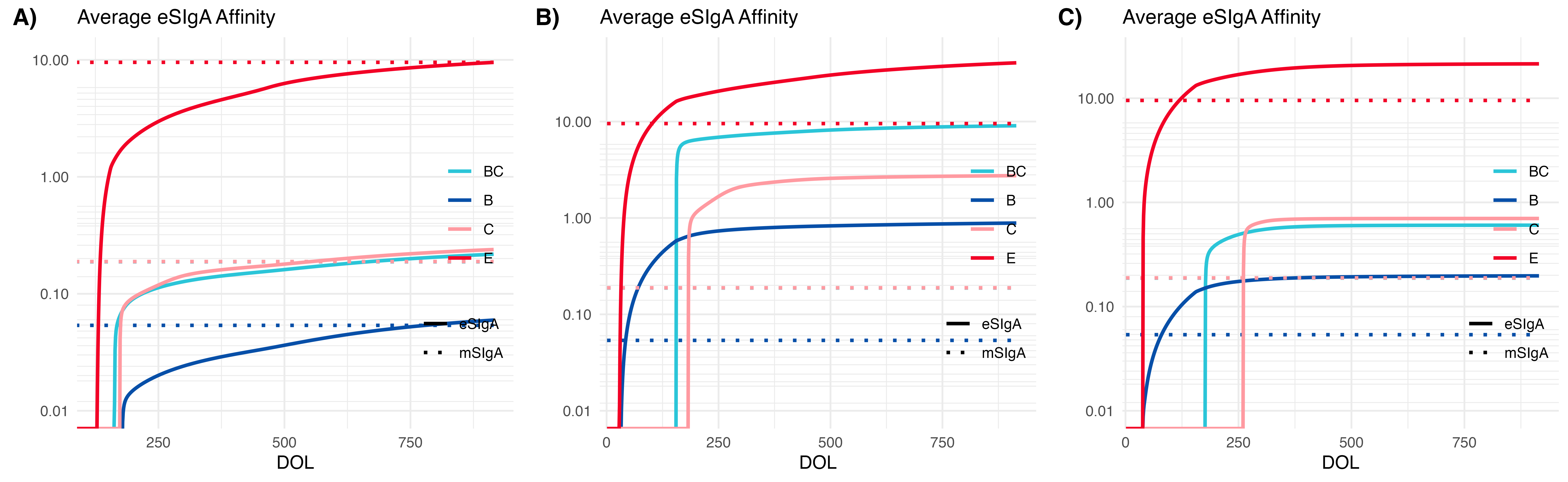

Supplement: S6 Fig — Temporal progression of average endogenous SIgA (eSIgA) affinities for (A) control, (B) when mSIgA levels are 85% reduced, (C) when mSIgA levels are 85% reduced with TLR4 antagonists’ administration (90% reduction in TLR4 stimulation). DOL: Day of life; E: Enterobacteriaceae; B: Bifidobacteriaceae; BC: Bacteroidaceae; C: Clostridiales. The data underlying this figure can be found in https://doi.org/10.5281/zenodo.15629746. (TIF) [file pbio.3003263.s006.tif]

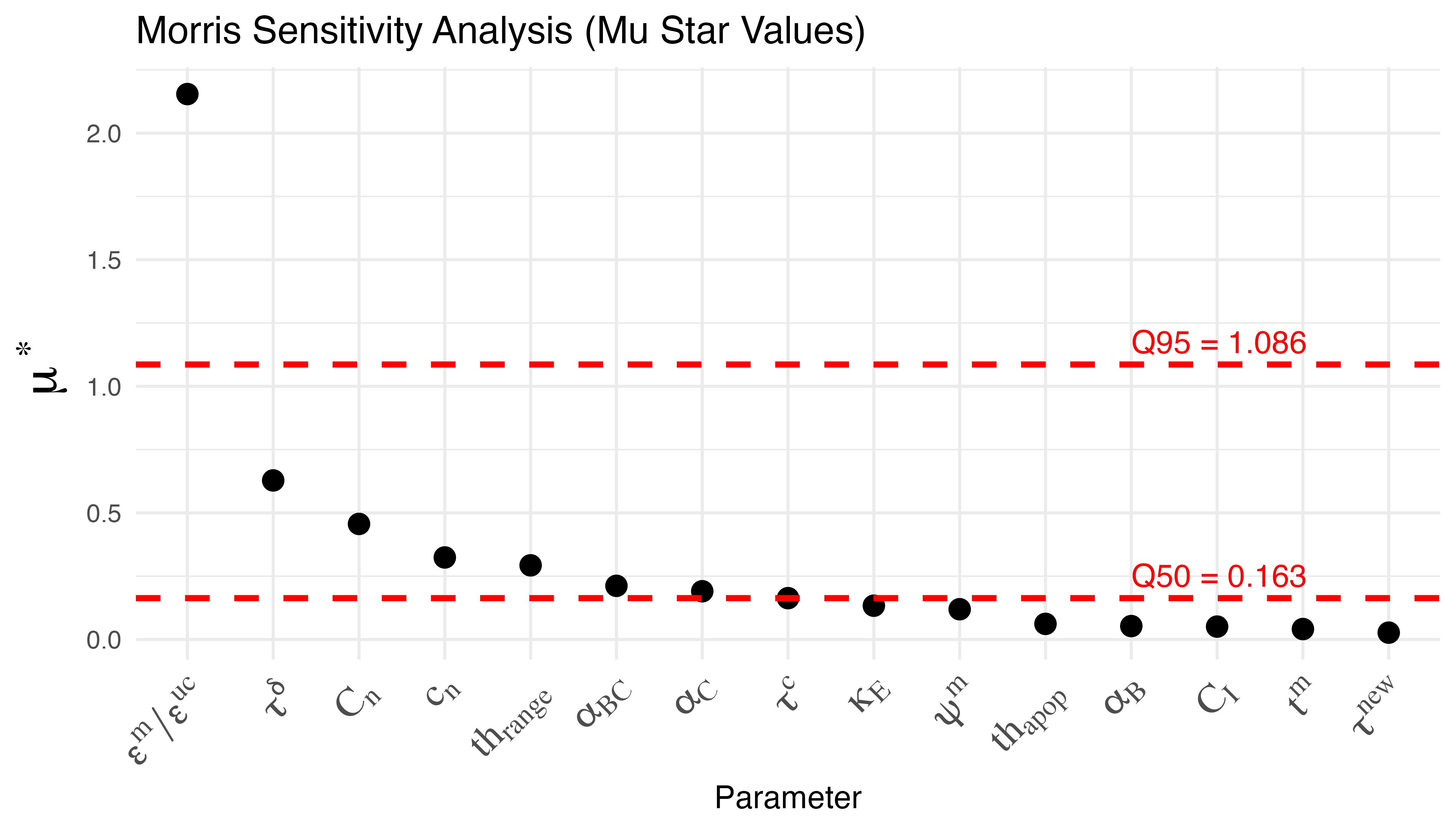

Supplement: S7 Fig — Visualization of the global sensitivity analysis presented in S5 Table. Red dashed lines represent the 50th and 95th percentiles to distinguish the most influential parameters. The data underlying this figure can be found in https://doi.org/10.5281/zenodo.15629746. (TIF) [file pbio.3003263.s007.tif]

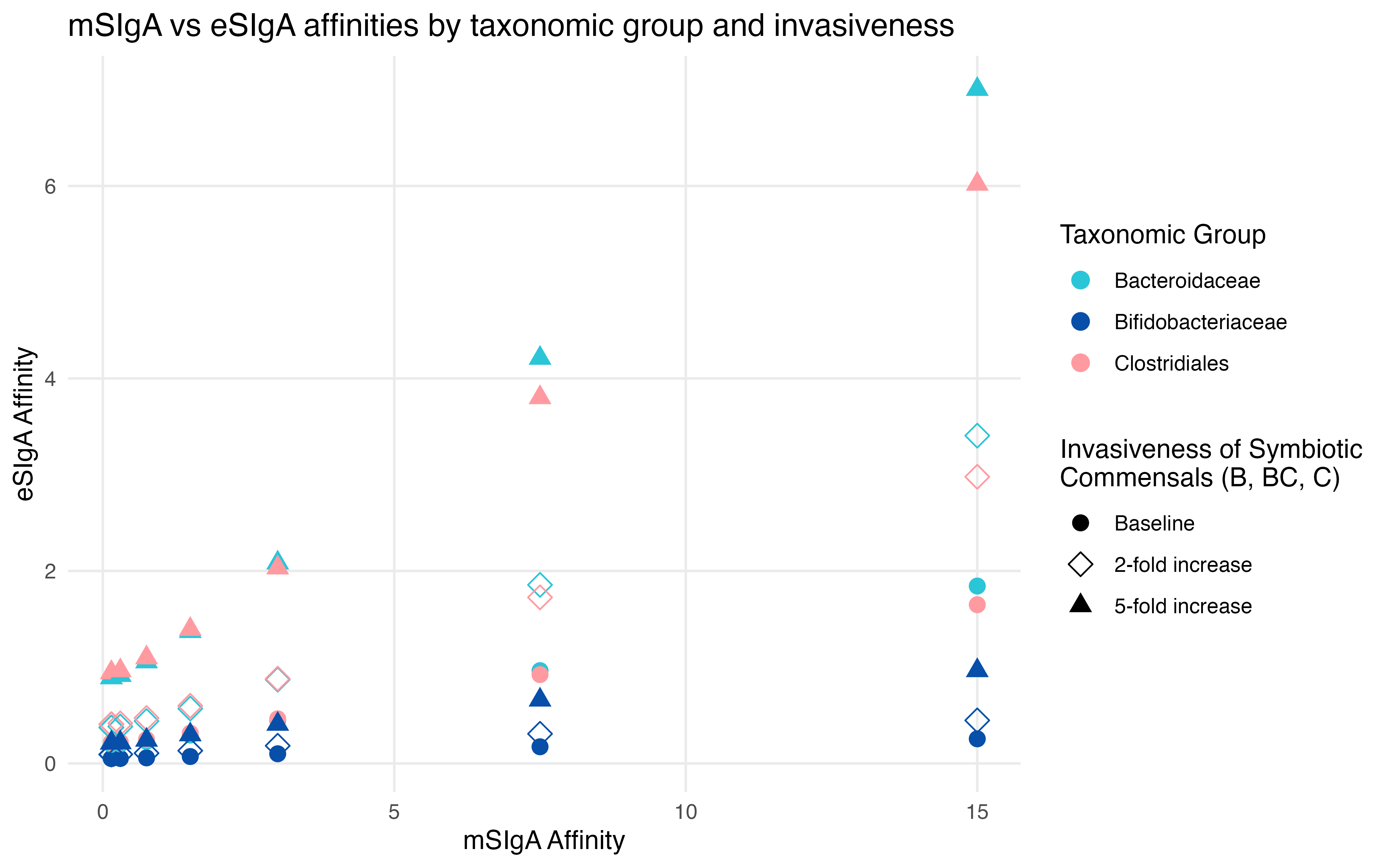

Supplement: S8 Fig — Endogenous versus maternal affinity levels (eSIgA vs. mSIgA) across taxonomic groups and varying levels of invasiveness. Each point represents the converged eSIgA affinity level for a given taxon under different levels of invasiveness, with shape denoting the degree of invasiveness (baseline, 2-fold, or 5-fold increase) and color indicating taxonomic group (Bacteroidaceae, Bifidobacteriaceae, Clostridiales). As invasiveness increases, uncoated bacteria more readily access GALT inductive sites, resulting in enhanced dendritic cell activation and a higher Tfh:Tfr ratio, which promotes increased eSIgA affinity. The data underlying this figure can be found in https://doi.org/10.5281/zenodo.15629746. (TIF) [file pbio.3003263.s008.tif]

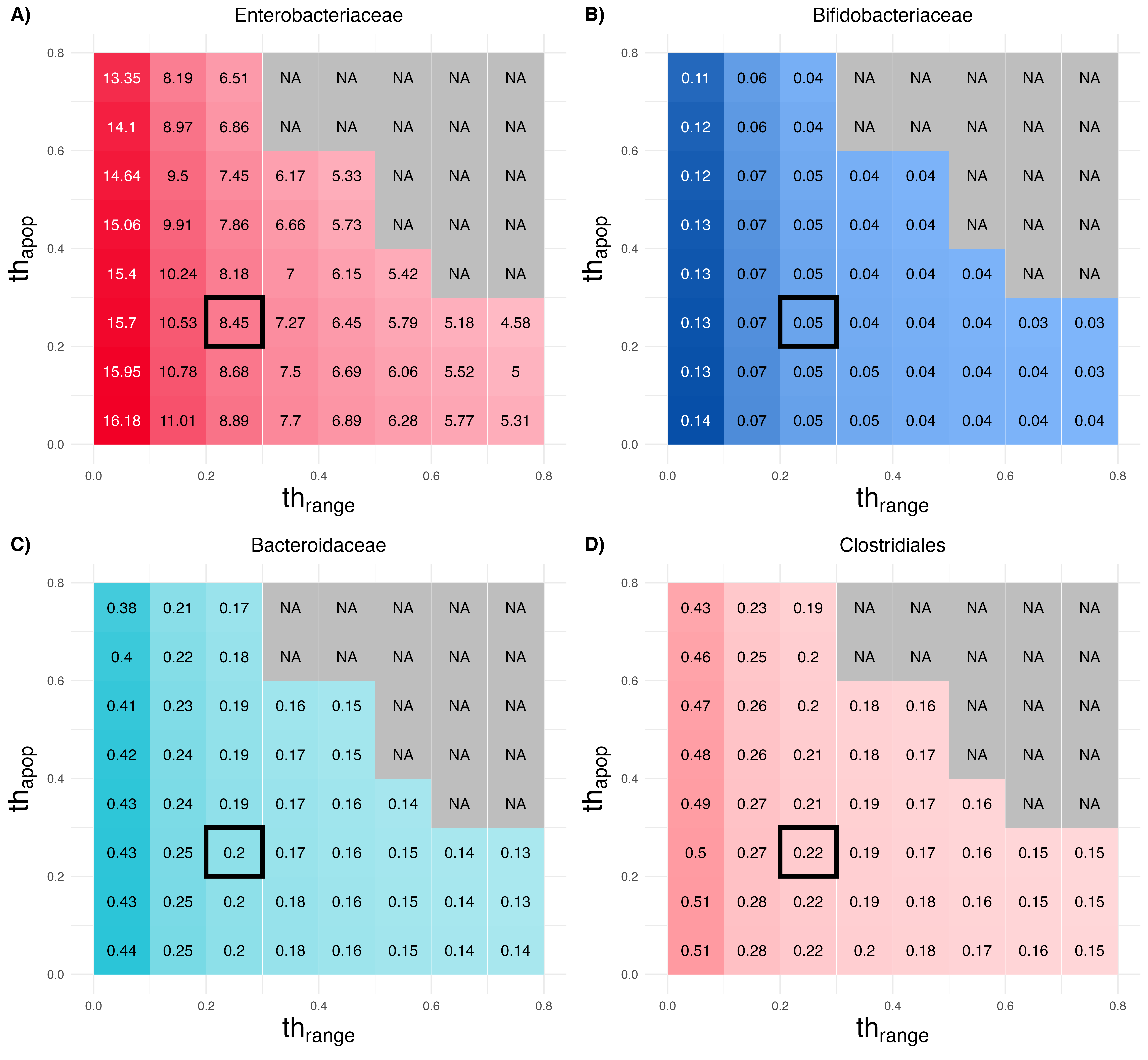

Supplement: S9 Fig — Heatmaps of average endogenous SIgA (eSIgA) affinity values at the end of 735 days (2 years) for 0.05≤thrange≤0.75 and 0.05≤thapop≤0.75 for A) Enterobacteriaceae, B) Bifidobacteriaceae, C) Bacteroidaceae and D) Clostridiales. Colors represent the magnitude of the eSIgA affinity values, with darker colors indicating larger values. Numerical values are indicated in each box. NA represents {thrange,thapop} combinations with 1−thrange≥thapop constraint. Baseline values (thrange=0.25 and thapop=0.25) are indicated with the bold black boxes. Note that all affinity values targeting the symbiotic commensals (Bifidobacteriaceae, Bacteroidaceae, Clostridiales) are below 1, reflecting their predominantly masking behavior. This figure demonstrates the robustness of our affinity maturation model, showing that the exact numerical values of thapop and thrange do not affect the functional properties of the endogenous antibodies. The data underlying this figure can be found in https://doi.org/10.5281/zenodo.15629746. (TIF) [file pbio.3003263.s009.tif]

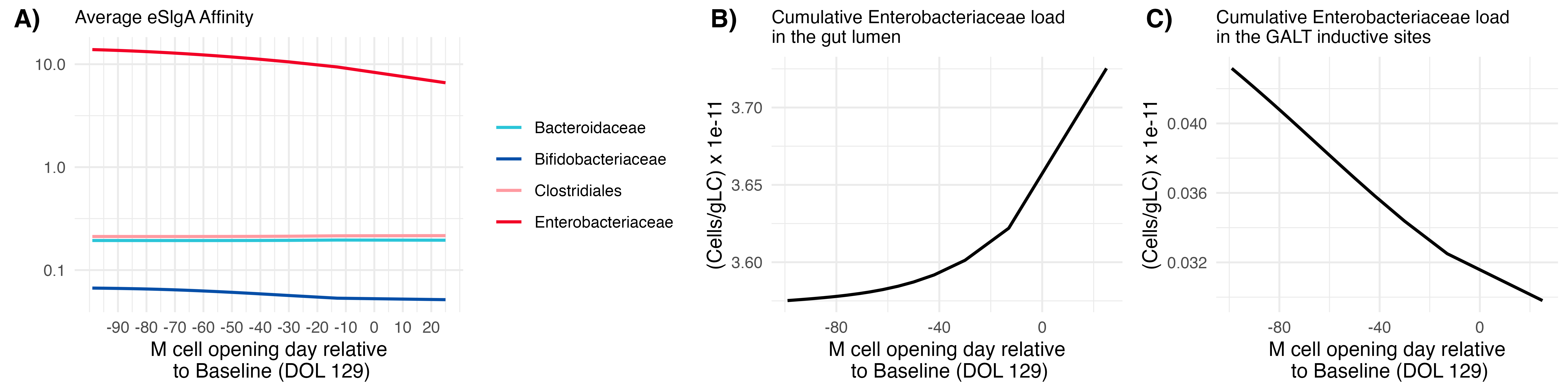

Supplement: S10 Fig — A) Average eSIgA Affinity values, B) Cumulative Enterobacteriaceae load in the gut lumen, and C) Cumulative Enterobacteriaceae load in the GALT inductive sites over the course of 2 years (735 days) for different delay durations of M cell opening relative to the baseline M cell opening time (DOL 129). DOL: Day of life. Early M cell opening increases Enterobacteriaceae antigen recovery in GALT inductive sites, potentially heightening susceptibility to enteric infections, yet triggers a more aggressive affinity maturation process against Enterobacteriaceae, reducing their cumulative burden in the gut lumen over time. Immune responses against symbiotic commensals remain largely unaffected. The data underlying this figure can be found in https://doi.org/10.5281/zenodo.15629746. (TIF) [file pbio.3003263.s010.tif]

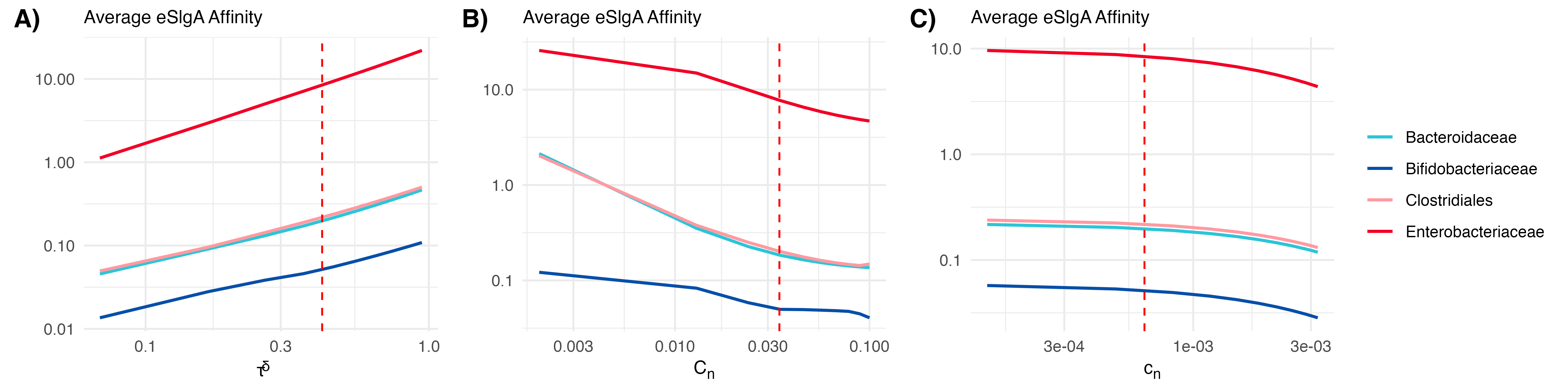

Supplement: S11 Fig — Sensitivity of the average endogenous SIgA (eSIgA) affinity values at the end of 735 days (2 years) in response to A) the multiplier to adjust the incremental increase in the selection threshold during GC reactions (τδ), and B) the amplitude (Cn) and C) the decay rate (cn) of the exponential function describing the diminishing pool of naïve T and B cells. Red dashed line marks the baseline values. Note that both axes are shown on logarithmic (base 10) scales. The data underlying this figure can be found in https://doi.org/10.5281/zenodo.15629746. (TIF) [file pbio.3003263.s011.tif]

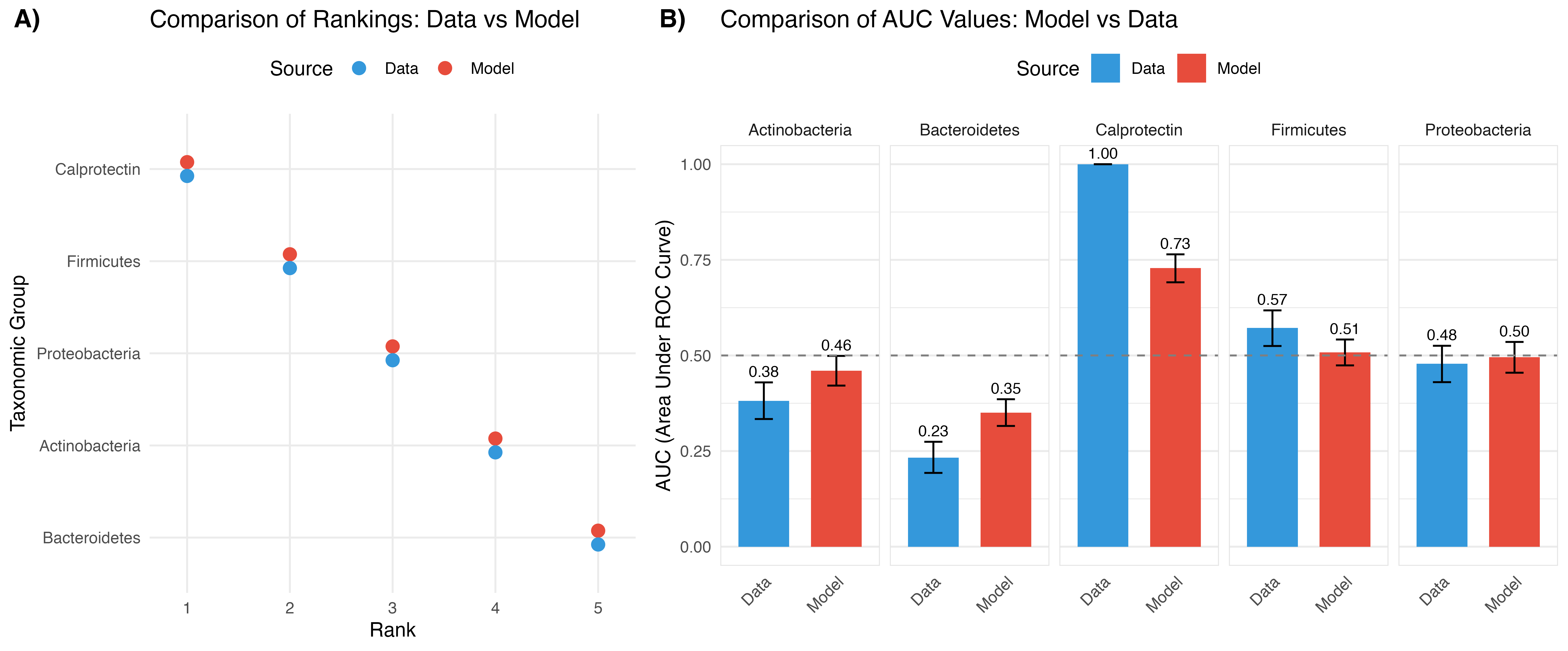

Supplement: S12 Fig — A) Comparison of rankings for predictors between experimental data (blue) and model predictions (red). Connected points indicate the same predictor, with horizontal position showing rank order (1–5) in predictive importance. Identical ranking of paired points demonstrates the strong agreement between the model and the cohort data. B) Comparison of AUC (Area Under ROC Curve) values for cohort data (blue) and model predictions (red). Error bars represent the standard error. Calprotectin shows the highest predictive power in both datasets, while taxonomic groups demonstrate a moderate predictive performance. The dashed horizontal line at 0.5 represents the threshold for random prediction. The data underlying this figure can be found in https://doi.org/10.5281/zenodo.15629746. (TIF) [file pbio.3003263.s012.tif]
