# Supplementary material for: A model of early-life interactions between the gut microbiome and adaptive immunity provides insights into the ontogeny of immune tolerance
Source: PLoS Biol. 2025 Aug 14;23(8):e3003263. doi: 10.1371/journal.pbio.3003263 (PMC12352683; doi:10.1371/journal.pbio.3003263)
Supplement: S1 Table — Summary of the taxonomic classification methods employed in studies used for inference, including details on the reference databases used, subjects, selection criteria, clustering methods, and bioinformatic tools. (DOCX) [file pbio.3003263.s013.docx]

| **Study** | **Data used** | **Unit** | **Subjects** | **Selection** | **Taxonomic Classification** |
| --- | --- | --- | --- | --- | --- |
| Tsukuda et al. [1] | Relative abundances during the first 2 years of life. | Ratios | Fecal samples of 12 human subjects during the first 2 years of life. | All subjects except Subject ID K (transition to solid food is not recorded.) were used since there were no subjects free of both antibiotic and probiotic exposure. Characteristics are provided in S1 Table of the respective study. | Taxonomy was assigned using the SILVA database (Release 138) with a 50% bootstrap threshold via the Qiime feature-classifier. Phylotypes were clustered using open-reference clustering against the NCBI 16S RefSeq records​. |
| Palmer et al. [2] | Bacterial counts to scale the relative abundances to absolute abundances. | Cells/g of stool | Fecal samples of 14 healthy, full-term human infants during the first year of life. | Babies #7 and #9 who were delivered by vaginal birth, exclusively breastfed, and not exposed to antimicrobials. | Each sequence was taxonomically classified using the 2004 prokMSA taxonomy via BLAST alignment. This method compared sequences from 16S rRNA gene sequencing to the prokMSA taxonomy to assign taxonomic ranks​. |
| Planer et al. [3] | IgA indexes for the Maternal Phase (days 60 and 120) and Steady Phase. | Unitless | Fecal samples of 40 healthy human twin pairs during the first 2 years of life. | Subjects are selected based on their 'Ratio of Breast milk: Formula' for the exclusive breastfeeding and mixed feeding periods. No information regarding antibiotic or probiotic exposure is provided. | Taxonomy was assigned using operational taxonomic units (OTUs) clustered with 97% identity against the GreenGenes 2013 reference database, analyzed with QIIME version 1.8. An abundance-filtered dataset was generated, only considering OTUs with relative abundances above 0.1% in at least 1% of the samples​. |
| Pan et al. [4]. | Relative abundances for the case of no breastfeeding at day 30. | Ratios | Fecal samples of 100 healthy human newborns collected at day 3 and days 30-42 of life. | Analysis results based on all subjects are used. None of the subjects were on perinatal antibiotics. No probiotic data provided. | Sequences were clustered into OTUs using 16S rRNA sequencing, and classification was performed with Qiime 1.9.1. The reference database for genus-level classification was not specified. |
| van der Waaij et al. [5] | IgA coating ratio of the Steady Phase. | Ratio | Fecal samples of 15 healthy human adults (non-inflammatory controls in this study). | Analysis results based on non-inflammatory controls are used. Patients and controls had not used antibiotics within 2 weeks of sampling. No probiotic data provided. | This study did not use 16S rRNA sequencing. Instead, it relied on flow cytometry for measuring immunoglobulin-coated bacteria, without employing genus calling techniques. |
