# Supplementary material for: A model of early-life interactions between the gut microbiome and adaptive immunity provides insights into the ontogeny of immune tolerance
Source: PLoS Biol. 2025 Aug 14;23(8):e3003263. doi: 10.1371/journal.pbio.3003263 (PMC12352683; doi:10.1371/journal.pbio.3003263)
Supplement: S2 Table — (DOCX) [file pbio.3003263.s014.docx]

| **Family / Order** | **Genus** | **Inoculation** | $O_{2}$ **metabolism** | **Carbohydrate metabolism** |
| --- | --- | --- | --- | --- |
| *Enterobacteriaceae* | *Escherichia-Shigella* | At birth. | Facultative anaerobes, ${\phi_{E}}^{O_{2}}=0.$ | Mostly PDPs, ${\phi_{E}}^{HMOs}$ and ${\phi_{E}}^{PDPs}$ are inferred from data. |
| *Bifidobacteriaceae* | *Bifidobacterium* | At birth and with milk within the first 3 days. | Strict anaerobes, ${\phi_{B}}^{O_{2}}=-1.$ | Mostly HMOs, but also PDPs, ${\phi_{B}}^{HMOs}$=1 and ${\phi_{B}}^{PDPs}$ are inferred from data. |
| *Bacteroidaceae* | *Bacteroides* | With mixed feeding. | Strict anaerobes, ${\phi_{BC}}^{O_{2}}=-1.$ | Mostly PDPs, but also HMOs, ${\phi_{BC}}^{HMOs}$ is inferred from data and ${\phi_{BC}}^{PDPs}=1.$ |
| *Clostridiales* | *Anaerostipes, Blautia, [Eubacterium] hallii group, Faecalibacterium, Ruminococcus* | With mixed feeding. | Strict anaerobes, ${\phi_{C}}^{O_{2}}=-1.$ | Mostly PDPs, but also HMOs, ${\phi_{C}}^{HMOs}$ is inferred from data and ${\phi_{C}}^{PDPs}=1.$ |
