# Supplementary material for: A model of early-life interactions between the gut microbiome and adaptive immunity provides insights into the ontogeny of immune tolerance
Source: PLoS Biol. 2025 Aug 14;23(8):e3003263. doi: 10.1371/journal.pbio.3003263 (PMC12352683; doi:10.1371/journal.pbio.3003263)
Supplement: S3 Table — A priori distributions for τc and τnew are parametrized to reflect the significant contribution of somatic hypermutation (SHM) relative to the contribution of newly activated B cells in increasing BCR diversity. While some degree of affinity maturation can occur in the absence of germinal centers, the extent and efficiency of this process are substantially greater with SHM. SHM targets the variable regions of BCR genes for high-rate mutations, leading to a dramatic increase in BCR diversity and specificity. This mechanism far surpasses the initial diversity provided by newly activated B cells through V(D)J recombination, crucially enhancing the immune system’s ability to fine-tune and strengthen responses to specific antigens [6]. Γ(α,β) denotes the gamma distribution, where α and β denote the shape and the rate parameter, respectively. β(α,β) denotes the beta distribution, where α and β denote the shape parameters. Ɲ(μ,σ) denotes the normal distribution, where μ and σ denote the mean and the standard deviation, respectively. U(a,b) denotes the uniform distribution, where a and b denote the upper and lower bounds, respectively. (DOCX) [file pbio.3003263.s015.docx]

| **Notation** | **Description** | **Units** | **Quantification** | **Range/**  **Constraints** | **Prior Dist./**  **Value** | **Model fit estimate** |
| --- | --- | --- | --- | --- | --- | --- |
| **State Variables** | | | | | | |
| $mSIgA$ | Maternal SIgA concentration in the gut lumen. | Unitless | - | [0,1] |  |  |
| $eSIgA$ | Endogenous SIgA concentration in the gut lumen. | Unitless | - | [0,1] |  |  |
| $O_{2}$ | Oxygen concentration in the gut lumen. | Unitless | - | [0,1] |  |  |
| ${y_{i}}^{L}$ | Absolute abundance of taxon $i$ in the gut lumen. | Cells/gLC | - | (0, ∞) |  |  |
| ${y_{i}}^{L, uc}$ | Absolute abundance of SIgA- taxon $i$ in the gut lumen. | Cells/gLC | - | (0, ∞) |  |  |
| ${y_{i}}^{L, c}$ | Absolute abundance of SIgA+ (C) taxon $i$ in the gut lumen. | Cells/gLC | - | (0, ∞) |  |  |
| ${y_{i}}^{L, \sum n}$ | Cumulative absolute abundance of neutralized taxon *i* in the gut lumen. | Cells/gLC | - | (0, ∞) |  |  |
| $\delta_{i}$ | Selection threshold for taxon *i*. | Unitless | - | (0, ∞) |  |  |
| ${B_{i}}^{n}$ | Number of naïve B cells available for entering circulation, dedicated to taxon *i*. | Unitless | - | (0, ∞) |  |  |
| ${B_{i}}^{c}$ | Number of circulating B cells within the GCs, dedicated to taxon $i$. | Unitless | - | (0, ∞) |  |  |
| ${B_{i}}^{p}$ | Number of plasma cells in LP, dedicated to taxon $i$. | Unitless | - | [0,1] |  |  |
| $\bar{{\rho_{i}}^{c}}$ | Average BCR affinity of circulating B cells, dedicated to taxon $i$. | Unitless | - | [0, ∞) |  |  |
| $\bar{{\rho_{i}}^{p}}$ | Average BCR affinity of plasma B cells, dedicated to taxon $i$. | Unitless | - | [0, ∞) |  |  |
| ${\sigma_{i}}^{c}$ | Standard deviation of the BCR affinity distribution of circulating B cells, dedicated to taxon $i$. | Unitless | - | [0, ∞) |  |  |
| **Time-independent parameters** | | | | | | |
| $\mu_{O2}$ | Degradation rate of $O_{2}$ by facultative anaerobes. | 1/ (days x Cells/gLC) | Inferred-1 | (0,1) | $\Gamma$(10,6) | 1.35 |
| ${\phi_{i}}^{O_{2}}$ | Impact of factor $O_{2}$ on the net growth rate of taxon $i$. | Unitless | ABL [7] | {0, 1} | - | ${\phi_{E}}^{O_{2}}=0$  ${\phi_{B}}^{O_{2}}=-1$  ${\phi_{BC}}^{O_{2}}=-1$  ${\phi_{C}}^{O_{2}}=-1$ |
| ${\phi_{i}}^{HMOs, PDPs}$ | Impact of factor HMOs and PDPs on the net growth rate of taxon $i$. | Unitless | Inferred-1 | $0<{\phi_{E}}^{HMOs} <0.9$  ${\phi_{E}}^{HMOs}<{\phi_{C}}^{HMOs}<0.9$  ${\phi_{C}}^{HMOs}<{\phi_{BC}}^{HMOs}<0.9$  $0<{\phi_{E}}^{PDPs}<0.9$  ${\phi_{E}}^{PDPs}<{\phi_{B}}^{PDPs}<0.9$ | $\beta$(5,5) | ${\phi_{E}}^{HMOs}=0.31$  ${\phi_{BC}}^{HMOs}=0.61$  ${\phi_{C}}^{HMOs}=0.47$  ${\phi_{E}}^{PDPs}=0.48$  ${\phi_{B}}^{PDPs}=0.63$ |
| $\lambda_{i}$ | Net growth rate of taxon $i$. | 1/days | Inferred-1 | (0, ∞) | Γ (3,2) | $\lambda_{E}=1.323$  $\lambda_{B}=1.19$  $\lambda_{BC}=1.00$  $\lambda_{C}=1.34$ |
| $\vert\beta_{i,j}\vert$ | Magnitude of impact of taxon $j$ abundance on taxon $i$ abundance for each pair of $\{i,j\}$, where the directionality of impact is known *a priori.* | 1/ (days x Cells/gLC) | Inferred-1 | (0, ∞) | Γ (2,0.1) | $\beta_{E,E}=-56.61$  $\beta_{E,B}=-28.31$  $\beta_{E,BC}=-14.94$  $\beta_{E,C}=-24.40$  $\beta_{B,E}=-2.43$  $\beta_{B,B}=-5.92$  $\beta_{BC,E}=-6.16$  $\beta_{BC,BC}=-17.64$  $\beta_{C,E}=-1.90$  $\beta_{C,C}=-5.28$ |
| $\beta_{i,j}$ | Value of impact of taxon $j$ abundance on taxon $i$ abundance for each pair of $\{i,j\}$, where the directionality of impact is not known *a priori.* | 1/ (days x Cells/gLC) | Inferred-1 | (-∞, ∞) | $Ɲ$(0,10) | $\beta_{B,BC}=-3.70$  $\beta_{B,C}=-4.73$  $\beta_{BC,B}=10.67$  $\beta_{BC,C}=-15.02$  $\beta_{C,B}=-5.49$  $\beta_{C,BC}=-2.14$ |
| $\rho_{i}$ | Average mSIgA affinity, specific to taxon $i$. | Unitless | Inferred-1 | [9,99] if $i \in\{E\}$,  (0.10,0.25] if $i \in\{B\},$ (0,0.10] if $i \in\{BC, C\}.$ | U (9,99) if $i\in\{E\}$,  Γ(5,100) if $i\in\{B\}$,  Γ (20,100) if  $i\in\{BC, C\}$. | $\rho_{E} = 9.52$  $\rho_{B} = 0.05$  $\rho_{BC} = 0.18$  $\rho_{C} = 0.18$ |
| ${\mu_{i}}^{m, m}$ | Masking rate of mSIgA, specific to taxon $i$. | 1/days | Dependent | 0 if $i \in\{E\}$,  [0.8,1) if $i \notin\{E\}.$ | - | ${\mu_{E}}^{m, m}= 0.10$  ${\mu_{B}}^{m, m}= 0.95$  ${\mu_{BC}}^{m, m}= 0.84$  ${\mu_{C}}^{m, m}= 0.84$ |
| ${\mu_{i}}^{m, n}$ | Neutralizing rate of mSIgA, specific to taxon $i$. | 1/days | Dependent | [0.8,0.95] if $i \in\{E\}$,  0 if $i \notin\{E\}.$ | - | ${\mu_{E}}^{m, n}= 0.90$  ${\mu_{B}}^{m, n}= 0.05$  ${\mu_{BC}}^{m, n}= 0.16$  ${\mu_{C}}^{m, n}= 0.16$ |
| $r_{d}$ | Dissociation rate of SIgA | Unitless | Inferred-1 | (0, 1) | Γ (10,20) | 0.602 |
| $\omega_{i}$ | Binding ability of mSIgA, specific to taxon $i$. | Unitless | Dependent | [0, 1] | - | $\omega_{E}= 0.94$  $\omega_{B} = 0.43$  $\omega_{BC}= 0.49$  $\omega_{C} = 0.49$ |
| $\epsilon^{uc}$ | Antigenic-sampling rate ${y_{i}}^{L, uc}$ for any taxon $i$. | Unitless | Calibrated | $\epsilon^{c}$ > $\epsilon^{uc}$ | - | 0.005 |
| $\epsilon^{c}$ | Antigenic-sampling rate ${y_{i}}^{L, c}$ for any taxon $i$. | Unitless | Calibrated | $\epsilon^{c}$ > $\epsilon^{uc}$ | - | 0.05 |
| $\alpha_{i}$ | Relative invasiveness of taxon $i$. | Unitless | Inferred-2 | $\alpha_{E} >max\{\alpha_{BC} ,\alpha_{C} \} >\alpha_{B} ,$[8]. | Γ (2,250) if $i \in\{B\}$,  Γ (2,50) if $i \in\{BC, C\}$. | $\alpha_{E}=1$ (fixed)  $\alpha_{B}=0.008$  $\alpha_{BC}=0.036$  $\alpha_{C}=0.038$ |
| $\kappa_{i}$ | Relative immunostimulatory capacity of the SIgA- members of taxon $i$. | Unitless | Inferred-2 | A priori distribution is parameterized given the relative immunostimulatory capacities reported in the literature [5,9]. | Γ (30,0.1) if $i \in\{E\}$. | $\kappa_{E}=314.1$  $\kappa_{B}=1$ (fixed)  $\kappa_{BC}=1$ (fixed)  $\kappa_{C}=1$ (fixed) |
| $I_{i}$ | Binary variable representing the inherent anti-inflammatory potential of taxon $i$. | Unitless | ABL [10-12] | 0 if $i \in\{E\}$,  1 if $i \notin\{E\}.$ | - | $I_{E} = 0$  $I_{B} = 1$  $I_{BC} = 1$  $I_{C} = 1$ |
| $C_{n}$ | Amplitude of the exponential function describing the diminishing pool of naïve T and B cells. | Unitless | Calibrated | - | - | 0.03 |
| $c_{n}$ | Decay rate of the exponential function describing the diminishing pool of naïve T and B cells. | Unitless | Calibrated | - | - | 0.000634 |
| $th_{apop}$ | Selection threshold multiplier determining the minimum BCR affinity to get sufficient T cell help to avoid apoptosis. | Unitless | Calibrated | - | - | 0.25 |
| $th_{high}$ | Selection threshold multiplier determining the minimum BCR affinity to get sufficient T cell help to differentiate to plasma cells. | Unitless | Calibrated | - | - | 0.75 |
| $th_{ang}$ | Selection threshold multiplier determining the minimum BCR affinity for B cells to be rendered anergic through regulatory mechanisms. | Unitless | Calibrated | - | - | 1.25 |
| $r_{p}$ | Proliferation rate of circulating B cells in the dark zone of the Germinal Center. | 1/days | Calibrated | - | - | 1 (Rapidly proliferating B cells in the dark zone of the germinal centers have cell cycles typically between 6 and 12 h [13]. Here we assume a 12h cycle, meaning the cell count doubles within a day. ) |
| $\tau^{c}$ | Multiplier to calculate the additional standard deviation in BCR affinity distribution after somatic hypermutation. | Unitless | Inferred-2 | - | Γ (2,0.05) | 28.33 |
| $\tau^{new}$ | Multiplier to calculate the additional standard deviation in BCR affinity distribution in newly activated B cells. | 1/(Cells/gLC) | Inferred-2 | - | Γ (10,0.05) | 180.21 |
| $\tau^{\delta}$ | Multiplier to adjust the incremental increase in the selection threshold calculated in Eqn. 1.2.8. | Unitless | Inferred-2 | - | Γ (2,5) | 0.42 |
| $t^{m}$ | Time of M cell activation and the start of antigenic sampling. | Days | - | - | - | $(t^{50},30)$ |
| $t_{MF}$ | Time of switching to MF from EBF. | Days | - | [0,180] | - | - |
| ${t^{*}}_{MF}$ | Average time of switching to MF from EBF in the dataset used for parameter inference after data pre-processing. | Days | - | - | - | 154 |
| $T_{EBF}$ | Duration of EBF. | Days | - | [0,180] | - | - |
| $T_{MF}$ | Duration of MF. | Days | - | [0,300] | - | - |
| ${T^{*}}_{MF}$ | Average duration of MF in the dataset used for parameter inference after data pre-processing. | Days | - | - | - | 308 |
| $K_{H1}$ | $f_{HMOs}(t,t_{MF} ,T_{MF})$ parameter (Eqn. 1.1.18) | Unitless | Calibrated | - | - | 0.44 |
| $V_{0}$ | $f_{HMOs}(t,t_{MF} ,T_{MF})$ parameter (Eqn. 1.1.18) | Unitless | Calibrated | - | - | 0.2 |
| $c_{H}$ | $f_{HMOs}(t,t_{MF} ,T_{MF})$ parameter (Eqn. 1.1.18) | 1/days | Calibrated | - | - | 40 |
| $K_{C}$ | $f_{TOT}(t,{t^{*}}_{MF} ,{T^{*}}_{MF})$ parameter (Eqn. 1.1.19) | Unitless | Calibrated | - | - | 1.28 |
| $c_{c}$ | $f_{TOT}(t,{t^{*}}_{MF} ,{T^{*}}_{MF})$ parameter (Eqn. 1.1.19) | 1/days | Calibrated | - | - | 0.005 |
| $t_{C}$ | $f_{TOT}(t,{t^{*}}_{MF} ,{T^{*}}_{MF})$ parameter (Eqn. 1.1.19) | Days | Calibrated | - | - | 720 |
| $K_{I}$ | ${\Delta f}_{mSIgA}(t)$ parameter (Eqn. 1.1.21) | Unitless | Calibrated | - | - | 0.15 |
| $m_{I}$ | ${\Delta f}_{mSIgA}(t)$ parameter (Eqn. 1.1.21) | Unitless | Calibrated | - | - | 0.38 |
| $c_{I}$ | ${\Delta f}_{mSIgA}(t)$ parameter (Eqn. 1.1.21) | 1/days | Calibrated | - | - | 0.0316 |
| $C_{I}$ | Maximum secretion capacity of plasma cells. | Unitless | Inferred-2 | - | Γ (10,1) | 12.39 |
| $I_{hl}$ | Half Life of SIgA in the gut lumen. | Days | ABL [14] | - | - | 5 |
| $\psi^{c}$ | Activation rate of naïve B cells per unit of SIgA-antigen complex. | 1/(Cells/gLC) | Calibrated | - | - | 0.1 |
| $\psi^{uc}$ | Activation rate of naïve B cells per unit of SIgA-free antigen. | 1/(Cells/gLC) | Calibrated | - | - | 1 |
| $s^{m}$ | Observation rate of masked bacteria in fecal samples. | Unitless | Inferred-1 | [0,1] | $\beta$(5,4) | 0.48 |
| $s^{uc}$ | Observation rate of uncoated bacteria in fecal samples. | Unitless | Inferred-1 | [$s^{c}$, 1] | $\beta$(10,4) | 0.82 |
| $s^{n}$ | Observation rate of neutralized bacteria in fecal samples. | Unitless | Inferred-1 | [$s^{uc}$, 1] | $\beta$(20,4) | 0.85 |
| **Time-dependent parameters** | | | | | | |
| ${\lambda_{i}}^{adj}$ | Adjusted growth rate of taxon $i$ based on $\lambda_{i}$, ${\phi_{i}}^{x}$ , $O_{2}(t)$, $HMOs(t)$, and $PDPs(t)$. | 1/days | Dependent | Determined by Eqn. 1.1.4. | - | - |
| ${\mu_{i}}^{e, c}$ | Coating rate of taxon $i$ induced by endogenous SIgA. | 1/days | Dependent | Determined by Eqn. 1.1.1. | - | - |
| ${\mu_{i}}^{e, n}$ | Neutralization rate of taxon $i$ induced by endogenous SIgA. | 1/days | Dependent | Determined by Eqn. 1.1.2. | - | - |
| ${\omega_{i}}^{e, n}$ | Binding ability of neutralizing eSIgA, specific to taxon $i$. | Unitless | Dependent | Determined by Eqn. 1.1.3. | [0,1] | - |
| ${\omega_{i}}^{e, c}$ | Binding ability of coating eSIgA, specific to taxon $i$. | Unitless | Dependent | Determined by Eqn. 1.1.3. | [0,1] | - |
| $\sigma$ | Microenvironmental stimulation | Unitless | Dependent | Determined by Eqn. 1.1.8. | - | - |
| $t^{50}$ | Time point when mSIgA concentration falls below 50% of its peak value, representing the beginning of antigenic sampling and activation of the endogenous immune system. | Days | Dependent | - | - | - |
| ${z_{i}}^{uc}$ | IgA-free antigens transported to Peyer’s Patches through M cells. | Cells/gLC | Dependent | Determined by Eqn. 1.1.6. | - | - |
| ${z_{i}}^{c}$ | IgA-antigen immune complexes transported to Peyer’s Patches through M cells. | Cells/gLC | Dependent | Determined by Eqn. 1.1.7. | - | - |
| ${y_{fn}}^{L}$ | Abundance of facultative anaerobes in the gut lumen. | Cells/gLC | Dependent | - | - | - |
| $\psi_{i}$ | Total B cell activation rate dedicated to taxon $i$. | 1/Cells/gLC | Dependent | Determined by Eqn. 1.1.5. | - | - |
| ${B_{i}}^{c, new}$ | Newly activated B cells dedicated to taxon $i$. | Unitless | Dependent | $\psi_{i}{B_{i}}^{n}$ | - | - |
| ${w^{n}}_{i}$ | Absolute abundance of neutralized bacteria observed per fecal content for taxon $i$. | Cells/gFC | Dependent | Determined by Eqn. 1.1.14. | - | - |
| ${w^{m}}_{i}$ | Absolute abundance of masked bacteria observed per fecal content for taxon $i$. | Cells/gFC | Dependent | Determined by Eqn. 1.1.15. | - | - |
| ${w^{uc}}_{i}$ | Absolute abundance of uncoated bacteria observed per fecal content for taxon $i$. | Cells/gFC | Dependent | Determined by Eqn. 1.1.16. | - | - |
| $w_{i}$ | Absolute abundance of total bacteria observed per fecal content for taxon $i$. | Cells/gFC | Dependent | Determined by Eqn. 1.1.17. | - | - |
