# Supplementary material for: A model of early-life interactions between the gut microbiome and adaptive immunity provides insights into the ontogeny of immune tolerance
Source: PLoS Biol. 2025 Aug 14;23(8):e3003263. doi: 10.1371/journal.pbio.3003263 (PMC12352683; doi:10.1371/journal.pbio.3003263)
Supplement: S4 Table — (DOCX) [file pbio.3003263.s016.docx]

| Assumption | References |
| --- | --- |
| We assume that the initial exposure to microbes occurs at birth and that the intrauterine environment is sterile, although this traditional view has been recently challenged and it has been shown that bacteria existed as low-abundance, low-biomass and sparse populations, in utero bacterial colonization did occur during healthy pregnancy. | [15,16] |
| Although SIgA titers and the proportion of IgA-coated bacteria are generally higher in the small intestine, our analysis is based on fecal sample data, which predominantly reflects the microbial composition of the colon. We therefore assume that the IgA responses observed in our data represent a combined mucosal immune response, with a dominant contribution from colonic organized lymphoid tissues. Although some of the IgA coating may result from cross-reactivity—where antibodies generated in Peyer’s patches of the small intestine in response to small-intestinal bacteria bind to structurally similar antigens on colonic bacteria—the observed taxon specificity of IgA binding in fecal samples suggests that many of these bacteria are likely true immunogens, eliciting local IgA responses within gut-associated inductive sites in the colon.  Colonic patches, like Peyer’s patches, are organized inductive structures within the gut-associated lymphoid tissue (GALT). They exhibit key immunological features required for IgA induction, including M cell-containing follicle-associated epithelium, germinal centers with defined dark and light zones (as observed in canonical secondary lymphoid tissues and inducible mucosal lymphoid structures), high endothelial venules (HEVs), and segregated B and T cell areas. Given this structural and functional similarity, our model focuses on the germinal center dynamics within such organized lymphoid tissues in the gut mucosa, without explicitly distinguishing between small-intestinal and colonic sites. Note that while scientific nomenclature sometimes includes mesenteric lymph nodes (MLNs) within the definition of GALT, we follow the classification presented by Mörbe *et al.* [17], which distinguishes between GALT structures (Peyer's patches and colonic patches) embedded within the intestinal wall and the separate intestine-draining MLNs. Thus, our definition of GALT excludes MLNs. | [17-27] |
| Human SIgA is highly somatically hypermutated (SHM), and SHM is commonly linked to T cell-dependent (TD) germinal center (GC) reactions, although not completely excluding T-cell independent (TI) mechanisms. While the contribution of TI pathways is well established in mice, particularly in early life, significant differences exist between mice and human infants. In mice, mucosal immune structures like PPs develop postnatally, promoting stronger TI responses initially. In contrast, human PPs, colonic patches, and isolated lymphoid follicles (ILFs) are organized by 22 weeks of gestation, enabling earlier initiation of GC reactions and favoring TD pathways even in infancy. This greater maturity in human infants allows them to initiate germinal center (GC) reactions earlier than mice. Moreover, many bacterial outer membrane components, including carbohydrate structures like lipopolysaccharides (LPS), are often attached to surface proteins, which can trigger TD responses even in early life given that the model organism is mature enough to do so. In light of this evidence, we assume that all the IgA producing cells are the product TD GC reactions, and no class-switch-recombination occurs in the lamina propria. | [17,28-32] |
| In lymphoid organs associated with the gut, germinal centers (GCs) are chronically present. B-cell clones induced in early life are maintained through chronic GCs in the gut, which serve as the predominant reservoir responsible for SIgA-secreting plasma cell maintenance. | [32–35] |
| Germinal center-derived, affinity matured B cell responses towards defined antigens are important for the effects of SIgA on the microbiota. Therefore, SIgA responses to gut commensals are assumed to be antigen-specific. | [36] |
| We assume that different taxonomic groups induce SIgA with different affinities. It has been shown that different commensal bacterial species are coated by IgA to varying extents (IgA-Seq data from Planer *et al.* [3], used for model fitting). Although the differential coating suggests that IgA affinities are different for different bacterial taxa, it does not provide direct proof because the established techniques do not account for the biases that might arise from using relative abundances when sorting IgA-bound and -unbound fractions. However, recent work by Jackson *et al* [37] proposed a probabilistic scoring method designed to address these biases, including the use of relative abundances, by adjusting for the bacterial composition before sorting and quantifying the likelihood of IgA binding. By showing varying degrees of IgA binding to different commensal bacteria after adjusting for the biases described, this study indicates strong likelihood of differences in IgA affinities. Combined with strain-specific IgA responses in the gut [36], we believe that it is appropriate to assume the affinities would vary. | [3,36,37] |
