# Supplementary material for: A model of early-life interactions between the gut microbiome and adaptive immunity provides insights into the ontogeny of immune tolerance
Source: PLoS Biol. 2025 Aug 14;23(8):e3003263. doi: 10.1371/journal.pbio.3003263 (PMC12352683; doi:10.1371/journal.pbio.3003263)
Supplement: S5 Table — Results of the global sensitivity analysis using the Morris method. This table lists the parameters included in the analysis, their respective descriptions, the ranges used for sampling, their inferred or calibrated values in the model, units, and the sensitivity metrics: μ (mean elementary effect, representing the overall influence of each parameter), μ* (mean absolute elementary effect, indicating the magnitude of the parameter’s impact irrespective of direction), and σ (standard deviation of the elementary effects, representing the variability or nonlinearity in the parameter’s effect on the output). The output variable considered in this analysis is the average endogenous SIgA affinity at DOL 735 against symbiotic commensals (Bifidobacteriaceae, Bacteroidaceae, and Clostridiales). The design includes 500 trajectories with 20 steps per trajectory, resulting in 10,000 total model evaluations. (DOCX) [file pbio.3003263.s017.docx]

| **Parameter (S7 Fig)** | **Description** | **Range of sampling** | **Inferred /calibrated value used in model** | **Unit** | **mu (**$\mu$**)** | **mu.star (**$\mu^{*}$**)** | **sigma (**$\sigma$**)** |
| --- | --- | --- | --- | --- | --- | --- | --- |
|  | | | | | | | |
| $\epsilon^{m}/\epsilon^{uc}$ | Ratio of the antigenic-sampling rate of ${y_{i}}^{L, m}$ to ${y_{i}}^{L, uc}$ by M cells. Represents the selective bias of M cells for sampling IgA-bacteria complexes. | [1,100] | 10 | Unitless | -2.1382 | 2.1544 | 7.4271 |
| $\tau^{\delta}$ | Multiplier to adjust the incremental increase in the selection threshold calculated in Eqn. 1.2.8. | [0.069, 0.945] | 0.42 | Unitless | 0.5941 | 0.6282 | 2.2591 |
| $C_{n}$ | Amplitude of the exponential function describing the diminishing pool of naïve T and B cells. | [0.005, 0.1] | 0.03 | Unitless | -0.4487 | 0.4564 | 2.8103 |
| $c_{n}$ | Decay rate of the exponential function describing the diminishing pool of naïve T and B cells. | [2, 30] x 1e-4 | 6.34 x 1e-4 | Unitless | -0.1466 | 0.324 | 2.0277 |
| $th_{range}$ | The plasma cell differentiation range, where $th_{high} = 1-th_{range}$ and $th_{ang} = 1+th_{range}$. | [0.01, 0.50] | 0.25 | Unitless | 9E-04 | 0.2928 | 1.6526 |
| $\alpha_{BC}$ | Invasiveness of *BC*. | [0.0069, 0.0946] | 0.036 | Unitless | 0.2107 | 0.2118 | 0.7995 |
| $\alpha_{C}$ | Invasiveness of *C*. | [0.0069, 0.0946] | 0.038 | Unitless | 0.1844 | 0.1905 | 0.6545 |
| $\tau^{c}$ | Multiplier to calculate the additional standard deviation in BCR affinity distribution after somatic hypermutation. | [6.90, 94.6] | 28.33 | Unitless | 0.0095 | 0.1632 | 0.8823 |
| $\kappa_{E}$ | Relative immunostimulatory capacity of *E*. | [215.9, 395.4] | 358 | Unitless | 0.0793 | 0.1334 | 0.6623 |
| $\psi^{m}$ | Activation rate of naïve B cells per unit of SIgA-antigen complex. | [0.01, 1] | 0.1 | 1/(Cells/gLC) | -0.1028 | 0.1194 | 0.3938 |
| $th_{apop}$ | Selection threshold multiplier determining the minimum BCR affinity to get sufficient T cell help to avoid apoptosis. | [0.01,0.49] | 0.25 | Unitless | -0.0039 | 0.0616 | 0.3263 |
| $\alpha_{B}$ | Invasiveness of *B*. | [0.0014, 0.0189] | 0.008 | Unitless | 0.0508 | 0.0528 | 0.2423 |
| $C_{I}$ | Maximum secretion capacity of plasma cells. | [5.43, 15.7] | 12.39 | Unitless | -0.0451 | 0.0508 | 0.1541 |
| $t^{m}$ | Time of M cell activation and the start of antigenic sampling. | [30, 154] | 129 | Days | 0.036 | 0.0411 | 0.5526 |
| $\tau^{new}$ | Multiplier to calculate the additional standard deviation in BCR affinity distribution in newly activated B cells. | [108.5, 314.0] | 180.21 | 1/(Cells/gLC) | 0.0197 | 0.027 | 0.1761 |
