# Supplementary material for: A model of early-life interactions between the gut microbiome and adaptive immunity provides insights into the ontogeny of immune tolerance
Source: PLoS Biol. 2025 Aug 14;23(8):e3003263. doi: 10.1371/journal.pbio.3003263 (PMC12352683; doi:10.1371/journal.pbio.3003263)
Supplement: S6 Table — This table presents parameters ranking above the 50th percentile (Q50) in our global sensitivity analysis (S7 Fig). For each parameter, we provide its biological interpretation, functional role within the model, and its quantitative influence on model outcomes. (DOCX) [file pbio.3003263.s018.docx]

| **Notation** | **Description** | **Interpretation** |
| --- | --- | --- |
| $\epsilon^{m}$/$\epsilon^{uc}$ | The ratio of the antigenic-sampling rate of masked and uncoated bacterial antigens by M cells. | This ratio represents the selective bias of M cells for sampling IgA-bacteria complexes. As $\epsilon^{m}$/$\epsilon^{uc}$ increases, it promotes higher accumulation of IgA-coated bacterial antigens relative to uncoated ones in the GALT inductive sites. This leads to the relatively higher activation of tolerogenic dendritic cells compared to inflammatory dendritic cells, and consequently leading to a lower Tfh:Tfr ratio. This ratio, proxied by the ratio of uncoated to total antigens accumulating in the inductive sites in Eqn. 1.2.8, directly influences the selection threshold parameter $\delta_{i}$ that governs the scale of affinity maturation, thus directly influencing the development of endogenous affinity levels. Note that the effect of $\epsilon^{m}$/$\epsilon^{uc}$ is not taxon-specific. A detailed analysis of the impact of $\epsilon^{m}$/$\epsilon^{uc}$ is presented in Fig 6. |
| $\tau^{\delta}$ | Multiplier to adjust the incremental increase in the selection threshold calculated in Eqn. 1.2.8. | This parameter directly influences the selection threshold value $\delta_{i}$ (Fig 2C). Biologically, it represents how “stringent” the immunological signals (such as Tfh:Tfr cell ratio and the inflammation level) in the germinal center environment are in selecting B cells based on their receptor affinity. Higher values of $\tau^{\delta}$ indicates the immune system is exerting stronger selection — allowing B cells with higher affinity for antigen receive sufficient help to survive and proliferate–, leading to faster increases in BCR affinity per P-SHM-S cycle.  This parameter can vary based on factors affecting costimulatory molecule expression levels, T-B cell interaction quality, and the architecture of germinal center microenvironments. This parameter can be inferred from experiments that track the distribution of BCR affinities over multiple time points in controlled germinal center reactions where all other variables (antigen availability, inflammatory cytokines, initial BCR affinity distribution, and Tfh:Tfr ratio) are either also measured or standardized, potentially through immunization experiments. Single-cell technologies tracking the fate of individual B cell clones combined with mathematical modeling could estimate this parameter by fitting observed affinity trajectories. Note that this parameter represents an intrinsic selection rate of the GC reactions, meaning that it is not taxon-specific. |
| $C_{n}$ | Amplitude of the exponential function describing the diminishing pool of naïve T and B cells. | This parameter is a proxy for the initial size of the naïve T and B cell pool. As $C_{n}$ increases (decreases), there will be more (less) naïve B cells migrating from the bone marrow to the GALT inductive site, being activated by bacterial antigens, and turning into circulating GC B cells at each round of proliferation, somatic hypermutation, and selection (P-SHM-S) cycle. A higher influx of newly activated cells broadens the BCR affinity distribution of the circulating GC B cells – effectively introducing lower-affinity clones into the pool – which reduces the average BCR affinity falling within the selection thresholds, thus lowering the average endogenous SIgA (eSIgA) affinity. Conversely, if $C_{n}$ becomes too low (approaching 0), the number of cells available to differentiate into circulating or plasma cells may be insufficient, leading to negligible eSIgA secreting plasma cells. However, such extreme cases were excluded from our sensitivity analysis; the range for $C_{n}$ was chosen to ensure that the lower bound still supports circulating and plasma cell differentiation. Since the abundance of bacteria is regulated by eSIgA concentration and affinities, which in turn regulates the immunostimulatory tone of the microenvironment, value of $C_{n}$ influences the affinity maturation process through ecological and inflammatory feedback loops. |
| $c_{n}$ | Decay rate of the exponential function describing the diminishing pool of naïve T and B cells. | This parameter determines how fast the naïve T and B cells differentiate into effector cells. As $c_{n}$ increases (decreases), naïve cells will be depleted faster (slower), leading to a faster (slower) convergence of the BCR affinity values of the endogenous plasma cells. A faster convergence means that the host will exhaust its supply of naïve cells earlier, prematurely ending GC reactions and affecting the final BCR affinity values of endogenous plasma cells. With slower convergence, naïve cells remain available longer, allowing GC reactions to continue and potentially achieve different BCR affinity outcomes. |
| $th_{range}$ | The plasma cell differentiation range, where  $th_{high} = 1-th_{range}$ and $th_{ang} = 1+th_{range}$. | This parameter defines the relative width of the affinity window around the selection threshold ($\delta_{i}$) that leads to plasma cell differentiation (Fig 1C). When set to a value of 0.2 (20%), for example, cells with BCR affinities between 80% and 120% of the threshold value will differentiate into plasma cells. Larger values create a more permissive selection process, while smaller values enforce stricter selection pressure on the circulating B cells. Therefore, this parameter affects both the range of BCR affinity and the number of circulating B cells turning to plasma cells at each round of proliferation, somatic hypermutation, and selection (P-SHM-S) cycle. |
| $\alpha_{i}$ | Relative invasiveness of taxon $i$. | This parameter defines the taxon's ability to penetrate intestinal epithelial cells. As this value increases, the SIgA-**uncoated** bacterial load in the GALT inductive sites increases, which in turn increases the converged endogenous affinity level against the taxon. To reduce the impact of identifiability problems during inference, we fixed the invasiveness of *Enterobacteriaceae* to 1 ($\alpha_{E}=1$) and inferred the invasiveness of the symbiotic commensals *Bifidobacteriaceae*, *Bacteroidaceae*, and *Clostridiales* relative to the pathogenic taxon *Enterobacteriaceae*. Value and effects of $\alpha_{i}$ are taxon-specific, meaning that modulating $\alpha_{i}$ directly impacts the affinity maturation against taxon $i$ via increasing the cumulative uncoated to total bacterial antigen ratio in Eqn. 1.2.8. However, since altering the endogenous affinity against a specific taxonomic group affects not only its own abundance but also the abundance of other taxa through community interactions, it can produce broader indirect effects. |
| $\tau^{c}$ | Multiplier to calculate the additional standard deviation in BCR affinity distribution after somatic hypermutation. | This parameter adjusts the standard deviation of the BCR affinity distribution of circulating B cells after each round of somatic hypermutation (Fig 1, step 5) .  If $\tau^{c}$ is too high: The affinity distribution of B cells flattens, increasing the probability of extremely high or low affinity B cells. This reduces the proportion of cells falling within the selection thresholds, resulting in fewer B cells differentiating into plasma cells or continuing circulation.  If $\tau^{c}$ is too low: The B cell population may lack any clones within the plasma cell differentiation range (between $th_{high}\delta_{i}$ and $th_{ang}\delta_{i}$, Fig 1, step 4) or circulation range (between $th_{apop}\delta_{i}$ and $th_{high}\delta_{i}$, Fig 1). This outcome is biologically implausible since the selection pressure imposed by the T cells in the GCs is competitive and operates on a relative basis, resembling a rank-based selection process.  Therefore, this parameter requires careful calibration based on either:   1. Known plasma cell numbers and affinities from experimental data, or 2. Known taxonomic abundances, by observing the feedback loop between IgA and microbial populations (as done in our calibration process). |
