# Supplementary material for: A model of early-life interactions between the gut microbiome and adaptive immunity provides insights into the ontogeny of immune tolerance
Source: PLoS Biol. 2025 Aug 14;23(8):e3003263. doi: 10.1371/journal.pbio.3003263 (PMC12352683; doi:10.1371/journal.pbio.3003263)
Supplement: S1 Text — (DOCX) [file pbio.3003263.s019.docx]

Supplementary Material for

A model of early-life interactions between the gut microbiome and adaptive immunity provides insights into the ontogeny of immune tolerance

Burcu Tepekule^1*^, Ai Ing Lim^2^, C. Jessica E. Metcalf^1*^

^1^ Dept of Ecology and Evolutionary Biology, Princeton University, Princeton, New Jersey, USA.

^2^ Dept of Molecular Biology, Princeton University, Princeton, New Jersey, USA.

* [burcutepekule@gmail.com](mailto:burcutepekule@gmail.com) (BT); [cjmetcalf@princeton.edu](mailto:cjmetcalf@princeton.edu) (CJEM)

**The PDF file includes:**

Supplementary Text

S1 to S12 Figs

S1 to S6 Tables

References (1–75)

**S1 Table.** **Taxonomic classification methods used across the studies for inference.** Summary of the taxonomic classification methods employed in studies used for inference, including details on the reference databases used, subjects, selection criteria, clustering methods, and bioinformatic tools.

**S2 Table. Key taxonomic groups and their corresponding inoculation time, oxygen (O_2_), and carbohydrate metabolism.**

**S3 Table. Parameters with their corresponding descriptions, units, ranges, prior distributions, and model fit estimates.** *A priori* distributions for 𝜏^c^ and 𝜏^new^ are parametrized to reflect the significant contribution of somatic hypermutation (SHM) relative to the contribution of newly activated B cells in increasing BCR diversity. While some degree of affinity maturation can occur in the absence of germinal centers, the extent and efficiency of this process are substantially greater with SHM. SHM targets the variable regions of BCR genes for high-rate mutations, leading to a dramatic increase in BCR diversity and specificity. This mechanism far surpasses the initial diversity provided by newly activated B cells through V(D)J recombination, crucially enhancing the immune system's ability to fine-tune and strengthen responses to specific antigens [6]. Γ(α,β) denotes the gamma distribution, where α and β denote the shape and the rate parameter, respectively. β(α,β) denotes the beta distribution, where α and β denote the shape parameters. Ɲ(μ,σ) denotes the normal distribution, where μ and σ denote the mean and the standard deviation, respectively. U(a,b) denotes the uniform distribution, where a and b denote the upper and lower bounds, respectively.

**S1 Fig. Inference results of RStan.** 2 chains are used with 500 and 1000 for warm-up and total iterations, respectively. 24 of 1000 (2.4%) transitions ended with a divergence. (**A**) Relative abundance estimate results for the maternal phase, for 154 days of exclusive breastfeeding (EBF) and 308 days of mixed feeding (MF). (**B**) Relative abundance estimate results for the maternal phase, for 308 days of mixed feeding (MF) with no exclusive breastfeeding. (**C**) Relative abundance estimate results for the steady phase, for 154 days of exclusive breastfeeding (EBF) and 308 days of mixed feeding (MF). Shaded areas represent the 95% confidence intervals. The data underlying this Figure can be found in https://doi.org/10.5281/zenodo.15629746.

**S2 Fig.** **Heatmap of normalized predictor importance values across time points from random forest models.** Each cell shows the normalized importance of a predictor in explaining total fecal abundance of *Enterobacteriaceae* at a given time point (in days), as determined by conditional permutation importance in a random‑forest model. Predictors include eSIgA affinity and concentration against *Enterobacteriaceae*, and total abundance of *Bifidobacteriaceae*, *Bacteroidaceae*, and *Clostridiales* in the gut lumen. A random predictor was included as a negative control. Predictor importance values were normalized within each time point between 0 and 1. The most influential variable in any column is black (value = 1.00) and progressively lighter shades indicate lower relative importance. Numeric values are overlaid for clarity. Starting from month 6 (DOL 180), endogenous immune responses against symbiotic commensals combined with ecological competition become the primary regulators of *Enterobacteriaceae* population, exerting stronger selection pressure than the endogenous SIgA (eSIgA) responses to *Enterobacteriaceae* itself, as seen from the decreasing importance of both the affinity and the concentration of eSIgA against *Enterobacteriaceae*. By DOL 720, importance values are relatively evenly distributed across predictors, consistent with the similarity in predictive power between total and SIgA-bound *Enterobacteriaceae* in Fig 3E. The data underlying this Figure can be found in https://doi.org/10.5281/zenodo.15629746.

***Role of Early Life Infections***

Early life gut dysbiosis plays a critical role in shaping the immune repertoire later in life [8], which can be recapitulated by our model. As an illustrative example, we simulated early-life infections by giving a selective advantage to the *Escherichia-Shigella* genus (compartment E in our model) over *Bifidobacteriaceae* by reducing the human milk oligosaccharides (HMOs) to 25% of their typical concentration in breastmilk while keeping all other parameters constant (maternal SIgA levels and affinities, breastfeeding duration, and mixed feeding periods). As illustrated in S3 Fig, early life dysbiosis (overgrowth of *Escherichia-Shigella*, highlighted in the magenta circle) leads to more hyperreactive response against all commensals (highlighted in the magenta rectangle), but especially against *Bifidobacteriaceae* (relative to the baseline scenario presented in Fig 2) as they are most severely affected by the inflammation resulting from *Escherichia-Shigella* overgrowth. However, we also observe a dominantly hyperreactive response against *Bacteroidaceae* and *Clostridiales* (eSIgA affinity converging to levels above 1), since the immune response against these commensals are also impacted by the sustained inflammation initiated during the early dysbiosis, in line with our predictor importance analysis in Fig 4.

**S3 Fig. Effects of early life dysbiosis on microbiome composition and immune response dynamics.** Simulation of an early-life infection scenario by giving a selective advantage to the Escherichia-Shigella genus (compartment E) relative to Bifidobacteriaceae (compartment B) by reducing the human milk oligosaccharides (HMOs) to 25% of their normal concentration in breastmilk while keeping all other parameters constant (maternal SIgA levels and affinities, breastfeeding duration, and mixed feeding periods). Panel **A)** demonstrates the early life dysbiosis characterized by the overgrowth of E compared to data (magenta circle), which results in higher levels of sustained inflammation during early life (panel **B)**), leading to a more hyperreactive response against symbiotic commensals (magenta rectangle, panel **C)**). The data underlying this Figure can be found in https://doi.org/10.5281/zenodo.15629746.

**S4 Fig. Illustration of model inputs without mixed feeding and differential impacts of EBF and MF durations in determining eSIgA affinity across a comprehensive range of feeding durations.** (**A**) Model inputs demonstrating a sharp transition from exclusive breastfeeding (EBF) to exclusive solid feeding (ECF), with no mixed feeding (MF) period in between, including normalized maternal secretory immunoglobulin A (mSIgA) concentration, human milk oligosaccharide (HMOs) and plant-derived polysaccharides (PDPs) calorie inputs, and timing of the endogenous immune system activation over time. EBF: exclusive breastfeeding; MF: mixed feeding; ECF: exclusive complementary feeding. (**B**) log (eSIgA Affinity) values at steady state for different combinations of EBF and MF durations, where the solid black line demonstrates the case of no MF followed by EBF. DOL: Day of life; E: *Enterobacteriaceae*; B: *Bifidobacteriaceae*; BC: *Bacteroidaceae*; C: *Clostridiales*. The data underlying this Figure can be found in https://doi.org/10.5281/zenodo.15629746.

**S5 Fig. Comparison of various breastfeeding scenarios and their impact on endogenous affinity maturation.** Relative abundances in fecal samples, absolute abundances in the gut lumen, and temporal progression of average endogenous SIgA (eSIgA) affinities for (**A**)**-**(**C**) control, (**D**)**-**(**F**) hyperreactive mSIgA in breastmilk (BM), (**G**)**-**(**I**) SIgA deficient BM, (**J**)**-**(**L**) only exclusive complementary feeding (ECF), and (**M**)**-**(**O**) ECF with probiotic (*Bacteroidaceae* and *Clostridiales*) supplementation. DOL: Day of life; E: *Enterobacteriaceae*; B: *Bifidobacteriaceae*; BC: *Bacteroidaceae*; C: *Clostridiales*. The data underlying this Figure can be found in https://doi.org/10.5281/zenodo.15629746.

**S6 Fig. Hypothetical scenario demonstrating the administration of TLR4 Antagonists to prevent inflammatory imprinting when mSIgA in breastmilk is insufficient.** Temporal progression of average endogenous SIgA (eSIgA) affinities for (**A**) control**,** (**B**) when mSIgA levels are 85% reduced, (**C**) when mSIgA levels are 85% reduced with TLR4 antagonists’ administration (90% reduction in TLR4 stimulation). DOL: Day of life; E: *Enterobacteriaceae*; B: *Bifidobacteriaceae*; BC: *Bacteroidaceae*; C: *Clostridiales*. The data underlying this Figure can be found in https://doi.org/10.5281/zenodo.15629746.

**S4 Table. Additional assumptions implicit to the model structure.**

***Global Sensitivity Analysis***

We implemented a global sensitivity analysis using the Morris method, which is a variance-based approach suitable for models with a large number of parameters [38]. The Morris method uses an OAT (One-At-a-Time) design to evaluate the impact of each parameter by varying them individually across a specified range. For this analysis, we considered 14 parameters: $t^{m}$, $\psi^{m}$, $C_{I}$, $C_{n}$, $c_{n}$, $th_{apop}$, $th_{range}$, $\tau^{new}$, $\tau^{\delta}$, $\tau^{c}$, $\alpha_{B}$, $\alpha_{BC}$, $\alpha_{C}$, $\kappa_{E}$ and 1 composite parameter: $\epsilon^{m}/\epsilon^{uc}$. We set up the Morris design with 500 trajectories, 10 levels per factor, and a grid jump of 2, resulting in 10000 total model evaluations (500 trajectories times 20 steps per trajectory). We used the average endogenous SIgA affinity at DOL 735 against symbiotic commensals (*Bifidobacteriaceae*, *Bacteroidaceae* and *Clostridiales*) as the output variable. For parameters with a prior distribution (see S3 Table), lower and upper bounds of the sampling ranges are based on the 5th and 95th percentiles of their respective prior distributions. Results are provided in S5 Table, with the parameters' respective descriptions, ranges of sampling, inferred/calibrated values used in our model, units, and sensitivity metrics including $\mu$ (mean elementary effect, indicating the overall influence of each parameter), $\mu^{*}$(mean absolute elementary effect, which captures the magnitude of the parameter's impact regardless of direction), and $\sigma$ (representing the variability or nonlinearity of the parameter's effect on the output).

**S5 Table. Results of the global sensitivity analysis.** Results of the global sensitivity analysis using the Morris method. This table lists the parameters included in the analysis, their respective descriptions, the ranges used for sampling, their inferred or calibrated values in the model, units, and the sensitivity metrics: $\mu$ (mean elementary effect, representing the overall influence of each parameter), $\mu^{*}$ (mean absolute elementary effect, indicating the magnitude of the parameter’s impact irrespective of direction), and σ (standard deviation of the elementary effects, representing the variability or nonlinearity in the parameter’s effect on the output). The output variable considered in this analysis is the average endogenous SIgA affinity at DOL 735 against symbiotic commensals (*Bifidobacteriaceae*, *Bacteroidaceae*, and *Clostridiales*). The design includes 500 trajectories with 20 steps per trajectory, resulting in 10,000 total model evaluations.

The mean absolute elementary effect ($\mu^{*}$) values represent the overall sensitivity of each parameter, quantifying the average impact that changes in the parameter have on the model's output. Since the relative ranking of $\mu^{*}$ values is key for identifying the most sensitive parameters, we used the 50th percentile as a threshold to distinguish the most influential ones (S7 Fig). Parameters with a $\mu^{*}$ above this threshold, $\{\epsilon^{m}/\epsilon^{uc}, \tau^{\delta}, c_{n}, C_{n}, th_{range}, \alpha_{BC}, \alpha_{C}\}$, are considered to be in the top 50% of sensitivity, representing the most influential factors driving model behavior. These parameters describe the ratio of the antigenic-sampling rate of masked and uncoated bacterial antigens by M cells ($\epsilon^{m}/\epsilon^{uc}$), the multiplier to adjust the incremental increase in the selection threshold during GC reactions ($\tau^{\delta}$), decay rate ($c_{n}$) and amplitude ($C_{n}$) of the exponential function describing the diminishing pool of naïve T and B cells, the plasma cell affinity differentiation range ($th_{range}$), and the invasiveness of *Bacteroidaceae*, and *Clostridiales* ($\alpha_{BC}, \alpha_{C}$). S6 Table provides detailed explanations of these highest-ranked parameters from our global sensitivity analysis and discusses their impact on model outcomes to improve the interpretability of the fundamental model dynamics.

**S7 Fig.** **Visualization of the global sensitivity analysis.** Visualization of the global sensitivity analysis presented in S5 Table. Red dashed lines represent the 50th and 95th percentiles to distinguish the most influential parameters. The data underlying this Figure can be found in https://doi.org/10.5281/zenodo.15629746.

These parameters – $\epsilon^{m}/\epsilon^{uc}$, $\tau^{\delta}$, $C_{n}$, $c_{n}$, $th_{range}$, $\alpha_{BC}$ and $\alpha_{C}$ – are then examined in isolation through local sensitivity analyses (Figs 6, S11, S9 and S8), alongside other model parameters. These analyses allow us to assess how localized variations in each parameter affect the model's output while holding the others constant, providing detailed insights into the individual impact of these parameters on endogenous affinity values.

**S6 Table. Interpretation of the highest-ranked parameters in global sensitivity analysis and their impact on model dynamics.** This table presents parameters ranking above the 50th percentile (Q50) in our global sensitivity analysis (S7 Fig). For each parameter, we provide its biological interpretation, functional role within the model, and its quantitative influence on model outcomes.

***Local Sensitivity Analyses***

**Sensitivity to sampling rates for SIgA-antigen immune complexes and SIgA-free antigens by M cells (Eqns. 1.1.6-1.1.7, parameters** $\epsilon^{m}, \epsilon^{uc}$**):** $\epsilon^{m}$ and $\epsilon^{uc}$ represent the antigenic-sampling rate of SIgA-bound (masked, ${y_{i}}^{L, m}$) and SIgA-free (uncoated, ${y_{i}}^{L, uc}$) antigens by M cells for each taxon $i$, respectively (S3 Table). $\epsilon^{m}/\epsilon^{uc}$ is identified as the most influential parameter by the global sensitivity analysis. The $\epsilon^{m}>\epsilon^{uc}$constraint in our baseline scenario reflects the well-characterized role of M cells in creating a positive feedback loop for the sampling of SIgA-antigen immune complexes [39-41]. Varying the ratio of these rates ($\epsilon^{m}/\epsilon^{uc}$) allows us to explore the importance of this M cell mediated feedback loop in impacting how ‘immune education’ unfolds (Fig 6). When $\epsilon^{m}/\epsilon^{uc}=1$, i.e., when M cells do not distinguish between the SIgA-bound and SIgA-free antigens and sample SIgA-bound antigens with a rate **as low as** the SIgA-free ones, affinity levels against symbiotic commensals (*Bifidobacteriaceae*, *Bacteroidaceae* and *Clostridiales*) increase compared to the baseline case $\epsilon^{m}/\epsilon^{uc}=1$0, approaching 1 for *Bacteroidaceae* and *Clostridiales*, reflecting a bias toward predominantly neutralizing rather than non-neutralizing masking behavior (compare the type of SIgA function relative to the level of antibody affinity, Fig 2B). The selective bias of M cells for sampling IgA-bacteria complexes promotes the production of non-neutralizing masking endogenous SIgA for symbiotic commensals, which are consequently presented as SIgA-immune complexes to the immune system, resulting in a feedback loop favoring tolerogenic imprinting.

**Sensitivity to the invasiveness of symbiotic commensals (parameters** $\alpha_{\boldsymbol{B}}, \alpha_{BC}, \alpha_{C}$): $\alpha_{i}$ is the invasiveness parameter, representing the bacteria's ability to penetrate intestinal epithelial cells. As invasiveness increases, a larger proportion of the bacterial population reaches the GALT inductive sites without being coated by SIgA. A higher uncoated bacterial load activates more dendritic cells and skews the local immune environment toward stronger Tfh cell imprinting. Consequently, the Tfh:Tfr ratio increases, promoting greater endogenous SIgA affinity maturation against the respective taxon (S8 Fig). Among the invasiveness parameters, $\alpha_{BC}$ and $\alpha_{C}$ are identified as the 6th and 7th most influential parameters in the global sensitivity analysis, with importance values modestly exceeding the median across all parameters (S7 Fig).

**S8 Fig. Sensitivity analysis to the invasiveness parameter.** Endogenous versus maternal affinity levels (eSIgA vs. mSIgA) across taxonomic groups and varying levels of invasiveness. Each point represents the converged eSIgA affinity level for a given taxon under different levels of invasiveness, with shape denoting the degree of invasiveness (baseline, 2-fold, or 5-fold increase) and color indicating taxonomic group (*Bacteroidaceae*, *Bifidobacteriaceae*, *Clostridiales*). As invasiveness increases, uncoated bacteria more readily access GALT inductive sites, resulting in enhanced dendritic cell activation and a higher Tfh:Tfr ratio, which promotes increased eSIgA affinity. The data underlying this Figure can be found in https://doi.org/10.5281/zenodo.15629746.

**Sensitivity to the ranges that determine the T cell help for different B cell fates (*Modeling the role of Germinal Centers*, parameters** $th_{apop}, th_{high}, th_{ang}$**):** Parameters $th_{apop}, th_{high},$ and $th_{ang}$ determine the ranges for B cell apoptosis, circulation, and differentiation to plasma cells, respectively; and together they reflect the selection pressure imposed by the T cells in the germinal centers (reflected as a rank-based selection process). A sensitivity analysis on these ranges keeping all else calibrated for the baseline scenario illustrates the range of potential outcomes resulting from variations in the feedback loops between Tfh and Tfr cells, either leading to a more generous (wider BCR affinity range) or stringent (narrower BCR affinity range) selection (S9 Fig). To do so, we first define the plasma cell differentiation range ([$th_{high}\delta_{i}$, $th_{ang}\delta_{i}$]) symmetrically around $\delta_{i}$ ($\delta_{i}$ denotes the antigen-specific selection threshold parameter representing the cumulative T cell help that determines various B cell fates, including apoptosis, circulation, or plasma cell differentiation; illustrated in Fig 2C.) using only one parameter $th_{range}$, where $th_{high} = 1-th_{range}$ and $th_{ang} = 1+th_{range}$ (note that in S3 Table $th_{high} =0.75$ and $th_{ang}=1.25$, leading to $th_{range}=0.25$). We then run our model for $0.05\leq th_{range}\leq0.75$ and $0.05\leq th_{apop}\leq0.75$ where cells with BCR affinity < $th_{apop}\delta_{i}$ fail to compete for T cell help, and die through apoptosis (note that in S3 Table $th_{apop}=0.25$). We apply the constraint $1-th_{range}\geq th_{apop}$ to ensure non-overlapping ranges defining the probability of continued circulating and probability of terminal differentiation to plasma cells.

**S9 Fig. Sensitivity analysis to the ranges that determine the T cell help for different B cell fates.** Heatmaps of average endogenous SIgA (eSIgA) affinity values at the end of 735 days (2 years) for $0.05\leq th_{range}\leq0.75$ and $0.05\leq th_{apop}\leq0.75$ for **A)** *Enterobacteriaceae,* **B)** *Bifidobacteriaceae*, **C)** *Bacteroidaceae* and **D)** *Clostridiales*. Colors represent the magnitude of the eSIgA affinity values, with darker colors indicating larger values. Numerical values are indicated in each box. NA represents $\{th_{range}, th_{apop}\}$ combinations with $1-th_{range}\geq th_{apop}$ constraint. Baseline values ($th_{range}=0.25$ and $th_{apop}=0.25$) are indicated with the bold black boxes. Note that all affinity values targeting the symbiotic commensals (*Bifidobacteriaceae, Bacteroidaceae, Clostridiales)* are below 1, reflecting their predominantly masking behavior. This figure demonstrates the robustness of our affinity maturation model, showing that the exact numerical values of $th_{apop}$ and $th_{range}$ do not affect the functional properties of the endogenous antibodies. The data underlying this Figure can be found in https://doi.org/10.5281/zenodo.15629746.

**Sensitivity to the time point of M cell maturation relative to the steroid levels in the breastmilk (parameter** $t^{m}$**):** Timing of M cell maturation (parameter $t^{m}$) was calibrated based on qualitative information in the literature (see main text). However, it is still not clear when exactly mature M cells emerge in human infants. To assess the sensitivity of our model to parameter $t^{m}$, we varied $t^{m}$ from 30 to 154 days (time of weaning), all else being kept as in the baseline scenario (S10 Fig).

Early opening of M cells, when the *Enterobacteriaceae* load is still high in the gut lumen, leads to a higher bacterial antigen burden being recovered from the GALT inductive sites (S10C Fig), making the infant more susceptible to enteric infections. However, this high antigenic load triggers a more aggressive affinity maturation process against *Enterobacteriaceae*, increasing the eSIgA affinity levels targeting it (S10A Fig), and thereby reducing the cumulative *Enterobacteriaceae* burden in the gut lumen over 2 years (735 days). Although this might initially seem more favorable for the host, our model does not incorporate the detrimental downstream systemic effects of enteric infections that might result from the increased *Enterobacteriaceae* burden recovered from the GALT inductive sites (S10C Fig). Moreover, our model assumes that M cell maturation coincides with the ontogenic timeline of other immune cells capable of initiating an endogenous immune response and producing high-affinity antibodies. In the event of a desynchronization between M cells and other immune components, the immune system would not be ready to initiate such a response, and the cumulative *Enterobacteriaceae* burden in the gut lumen would not decrease as much as shown in S10B Fig. Immune responses against symbiotic commensals remain largely unaffected (S10A Fig), which aligns with our global sensitivity analysis that identified $t^{m}$ as the second least influential parameter. However, it is important to note that this analysis only considers the average eSIgA affinity against symbiotic commensals as its output metric and does not capture the potential downstream effects of $t^{m}$ on high pathogenic load.

**S10 Fig. Impact of early and delayed M cell opening on endogenous SIgA affinity maturation and *Enterobacteriaceae* load. A)** Average eSIgA Affinity values, **B)** Cumulative *Enterobacteriaceae* load in the gut lumen, and **C)** Cumulative *Enterobacteriaceae* load in the GALT inductive sites over the course of 2 years (735 days) for different delay durations of M cell opening relative to the baseline M cell opening time (DOL 129). DOL: Day of life. Early M cell opening increases *Enterobacteriaceae* antigen recovery in GALT inductive sites, potentially heightening susceptibility to enteric infections, yet triggers a more aggressive affinity maturation process against *Enterobacteriaceae*, reducing their cumulative burden in the gut lumen over time. Immune responses against symbiotic commensals remain largely unaffected. The data underlying this Figure can be found in https://doi.org/10.5281/zenodo.15629746.

**Sensitivity to the multiplier to adjust the incremental increase in the selection threshold calculation (parameter** $\tau^{\delta}$**):** Parameter $\tau^{\delta}$ is used in Eqn. 1.2.8 to adjust the incremental increase in the selection threshold $\delta_{i}$ in response to sampled antigens in GALT inductive sites. Biologically, it represents how “stringent” the immunological signals in the germinal center environment are in selecting B cells based on their receptor affinity (see S6 Table for detailed explanation). It is identified as the second most influential parameter by the global sensitivity analysis, supported by its impact on the eSIgA affinities against symbiotic commensals (*Bifidobacteriaceae*, *Bacteroidaceae* and *Clostridiales*) shown in S11A Fig.

**S11 Fig. Impact of parameters** $\tau^{\delta}$**,** $C_{n}$**, and** $c_{n}$ **on endogenous SIgA (eSIgA) affinities.** Sensitivity of the average endogenous SIgA (eSIgA) affinity values at the end of 735 days (2 years) in response to **A)** the multiplier to adjust the incremental increase in the selection threshold during GC reactions ($\tau^{\delta}$), and **B)** the amplitude ($C_{n}$) and **C)** the decay rate ($c_{n}$) of the exponential function describing the diminishing pool of naïve T and B cells. Red dashed line marks the baseline values. Note that both axes are shown on logarithmic (base 10) scales. The data underlying this Figure can be found in https://doi.org/10.5281/zenodo.15629746.

**Sensitivity to the decay rate and the amplitude of the exponential function describing the diminishing pool of naïve T and B cells (parameters** $C_{n}$ **and** $c_{n}$**):** $C_{n}$ denotes the amplitude, and $c_{n}$ denotes the rate of decay of the pool of naïve T and B cells as the host ages and as these cells undergo imprinting. This process is modeled as an exponentially decreasing function of time $t$, $C_{n}e^{-c_{n}t}$. $C_{n}$ and $c_{n}$ are identified as the third and fourth most influential parameters by the global sensitivity analysis, respectively (S7 Fig). While $C_{n}$ represents the initial size of the naïve T and B cell pool – determining how **many** naïve B cells migrate from the bone marrow to join the circulating B cell pool and thus the GC reactions at each time point –, $c_{n}$ governs how **fast** the naïve B and T cells become activated and differentiate: naïve B cells joining the GC reactions and naïve T cells differentiating into effector subsets such as Tfh or Tfr cells. Together, they shape the affinity maturation process (see S6 Table for detailed explanation). A high $C_{n}$ introduces a higher number of newly activated B cells into the light zones of the germinal centers at each time step, broadening the BCR affinity distribution—effectively lowering the average affinity due to the high influx of lower-affinity clones (S11B Fig). A high $c_{n}$ depletes the naïve pool more rapidly, accelerating convergence and prematurely terminating GC reactions, resulting in reduced BCR affinity values of endogenous plasma cells (S11C Fig).

***Illustrative validation using an external dataset***

To assess the validity of our model outputs, we compared them against an independent, external dataset. This analysis serves as an illustrative example of how model predictions in Fig 3E can be externally evaluated by aligning them with real-world data trends. We examined the predictive power of microbial phyla and calprotectin levels in distinguishing celiac disease (CD, 2 subjects) from healthy controls (HC, 4 subjects) in infant gut microbiomes based on the data published by Olivares *et al.* [42]. We filtered subjects that were delivered vaginally, had no antibiotic exposure, and were breastfed until month 4, and used their samples collected at 6 months of age as predictors of disease state. Logistic regression models were used for each individual predictor (Abundances of *Proteobacteria*, *Actinobacteria*, *Bacteroidetes*, *Firmicutes*, and levels of Calprotectin), employing a cross-validation strategy with 3 folds and 100 repeats, while using upsampling to address class imbalance. Model performance was evaluated using Area Under the ROC Curve (AUC) with bootstrap-derived confidence intervals. Based on our model, we classified immunological outcomes as tolerant vs. not tolerant (affinities against all symbiotic commensals all below 1 simultaneously or not, respectively) and used this as a proxy for HC vs CD, respectively. We used 100 simulations where the breastfeeding duration randomly varied between 120 and 180 days to mimic the feeding patterns of the subjects. To represent a measurement taken within month 6, we sampled the predictor values at random days between day 150 and day 180. To calculate the AUC values at each iteration, we subsampled 6 subjects with 2 subjects being hyperreactive and 4 subjects being tolerant to resemble the data distribution. To prevent overfitting, we excluded any bootstrap iterations that produced AUC values of 1 during model performance evaluation. We then used the summary statistics of these AUC values. Our model showed good agreement with experimental data in terms of taxonomic rankings (S12A Fig), with no rank differences across all five predictors. Both model and data (S12B Fig) consistently identified Calprotectin as the strongest predictor (AUC of 1.00 in data and 0.73 on average in the model), which is expected given CD's inflammatory nature. More significantly, our model accurately reproduced the relative predictor importance patterns observed in the data across all taxonomic groups, providing a source of external validation for our model outcomes.

**S12 Fig. Comparison of predictive performance between cohort data and computational model.** **A)** Comparison of rankings for predictors between experimental data (blue) and model predictions (red). Connected points indicate the same predictor, with horizontal position showing rank order (1-5) in predictive importance. Identical ranking of paired points demonstrates the strong agreement between the model and the cohort data. **B)** Comparison of AUC (Area Under ROC Curve) values for cohort data (blue) and model predictions (red). Error bars represent the standard error. Calprotectin shows the highest predictive power in both datasets, while taxonomic groups demonstrate a moderate predictive performance. The dashed horizontal line at 0.5 represents the threshold for random prediction. The data underlying this Figure can be found in https://doi.org/10.5281/zenodo.15629746.

***Model Limitations***

**Model Complexity:** Our framework addresses a high-dimensional question related to early life immunity. In developing the model, we inevitably made decisions about its complexity, constrained by the qualitative and quantitative data available in the literature. Certain mechanisms, such as the detailed dynamics of T-cell-B-cell interactions in the gut lymphoid tissues, the regulation of commensal microbiota by IgA, and the influence of the multiple active components in breastmilk on early immune development remain open questions in the literature. Consequently, we adopted the most widely accepted explanations, which are detailed alongside underlying evidence in the main text and methods. To enable feasible parameter inference, we simplified some biological processes, such as excluding explicit representations of certain germinal center (GC) reactions (e.g., interactions with antigen-presenting cells and B cell antigen capture for T-cell presentation), abstracting the feedback mechanisms between Tfh, Tfr, and B cells, and generalizing interactions between IgA and microbial taxa to focus on overall effects rather than detailed microbe-antibody dynamics. Capturing early life dynamics requires these abstractions to be integrated with explicit exogenous and endogenous processes that occur at specific points during development, leading to a high dimensional model. The resulting model frame – which may appear both overly simplistic and overly complex - reflects a balance between minimizing identifiability issues during parameter inference while ensuring that the essential processes are adequately represented.

**Memory B cell compartment:** Our model does not include a distinct memory B cell compartment due to insufficient quantitative data for parametrizing B cell fate decisions between memory and plasma cell formation. Memory B cells are particularly important given the preferential differentiation of naive B cells to memory B cells during early life [43] and the contribution of neonatally induced circulating memory B cells to continuously shape the gut mucosal immune response [34]. However, persistent humoral memory to gut pathogens and commensals can be maintained either by long-lived memory B cells replenishing dying plasma cells or by long-lived plasma cells themselves [44]. Given that the composition of the microbiome remains relatively stable, and the nature of antigenic stimuli is consistent throughout adulthood (the Steady Phase in our model), both mechanisms would produce mathematically similar outcomes. Since our model assumes a stable microbiome composition as the Steady Phase, we effectively represented the continuous replenishment of plasma cells by memory cells by assigning a plasma cell death rate of zero at steady state. This approach maintains a stable plasma cell population even in the absence of the stimulating bacterial antigen, indirectly reflecting the role of the memory compartment in sustaining long-term humoral immunity. While this simplification captures the overall effect of memory B cells on sustained antibody production, it does not explicitly model the dynamics of the memory B cell population, which remains a limitation of our current framework.

**B cell selection and antigen diversity:** We assume that B cell interactions with different antigens occur in isolation. While in reality competition for multiple antigens simultaneously could influence B cell selection, our model does not account for such interactions. By treating each antigen independently, we simplify the dynamics of affinity maturation, which may overlook potential synergistic or competitive effects in response to multiple antigens. However, quantitative data to sufficiently parametrize such interactions is currently lacking in the field.

**Role of M cells during early life in humans:** Our assumptions of the timing of M cell maturation and its significant role in antigenic sampling heavily relies on mouse models, and evidence for M-cell based antigenic sampling for humans during early-life is almost non-existent. However, there is substantial evidence for the presence and function of M cells in adult humans. Gullberg *et al.* [45] characterized M cells in human Peyer's patches, identifying specific cell adhesion molecules and demonstrating their role in facilitating antigen uptake. Furthermore, Hase *et al.* [46] identified glycoprotein 2 (GP2) as a specific transcytotic receptor on M cells in both mice and humans, suggesting a conserved mechanism across species. These studies provide strong evidence for the existence and functional importance of M cells in human intestinal tissue. Our sensitivity analysis on the timing of M cell maturation quantitatively explores the possibility that M cell development in human infants may not significantly limit the efficacy of antigen sampling, simulating a scenario where M cells begin antigenic sampling as early as day 30.

**Additional effects of SIgA on community composition and antigenic sampling:** In addition to its masking role in our model, SIgA plays a crucial role in promoting niche colonization and stabilizing the gut microbiota. By creating temporal and spatial niches along the gastrointestinal tract, SIgA enhances microbial diversity and fosters beneficial host–microbiota interactions [39]. Additionally, Rollenske *et al.* showed that SIgA coating protects the microbiome from environmental stressors like bile toxicity and bacteriophages [47]. In our model, SIgA's colonization-promoting effects could theoretically increase the net growth rate of commensals, represented by a scaling coefficient. This adjustment would weaken colonization resistance in scenarios with insufficient IgA (either maternal or endogenous), as commensals would experience reduced growth. To maintain the same model fit in cases of no breastfeeding (and thus lack of maternal SIgA), re-calibrating the competition terms—likely by enhancing the neutralization of pathogens by commensals—would be necessary to preserve the longitudinal microbial abundances in the gut lumen. Additionally, SIgA likely influences antigenic sampling by modulating microbial proximity to the gut epithelium, beyond its masking role and M cell adhesion bias (which are incorporated in our model). In our model, this effect would modify the sampling rate based on the change in a particular taxon's proximity to the epithelium. Unfortunately, the quantitative data required to accurately capture these effects remain unavailable in the current literature.

**Innate immune mechanisms:** Our model focuses on adaptive immune responses and necessarily omits several critical processes that change dynamically over early-life. In particular, exclusion of innate immune mechanisms means leaving out the tolerogenic bias of the Pattern Recognition Receptors (PRRs) and Dendritic Cells during infancy, known to direct T-cells towards a largely 'anti-inflammatory' response [48]. This phenomenon will influence the fate of commensal-specific T cells, eventually affecting the endogenous antibody response. In our model, the absence of this temporal tolerogenic bias is likely to be numerically (though not mechanistically) compensated by the combination of anti-inflammatory effects of maternal antibody masking and the inherent anti-inflammatory properties of symbiotic commensals. While the potential overestimation of these anti-inflammatory properties may affect the binary immune outcome of a tolerogenic or hyperreactive state given the duration of different feeding practices, the remainder of our analysis and qualitative results and conclusions should remain robust against such biases.

**Cross-reactivity of SIgA:** While we model antigen-specific SIgA responses independently across taxonomic groups, this does not account for evidence that SIgA can exhibit cross-species and even cross-phyla reactivity. Such cross-reactivity may arise from shared epitopes between species, similar glycan motifs present on taxonomically distant bacteria, or a combination of canonical and noncanonical binding [49]. These mechanisms allow individual IgA clones to bind to diverse microbial taxa, potentially blurring the boundaries of taxon-specific immune targeting. However, the mechanistic basis of this “cross-species reactivity” remains unclear [49]. Quantitative data on the range and specificity of SIgA binding across microbial communities are still limited, making it difficult to parameterize cross-reactive binding events in a mechanistically grounded way. While our antigen-specific modeling approach preserves model tractability and captures key dynamics of interest, it likely underestimates the masking effect of low-affinity SIgA binding across symbiotic commensals, representing an important avenue for future model refinement.

**Impact of LPS structures on immune education:** Our model treats LPS as a generic inflammatory signal, primarily focusing on the immunostimulatory effects of hexa-acylated lipid A derived from Escherichia-Shigella. However, experimental evidence, notably from Vatanen *et al*. [50], demonstrates that LPS molecules vary significantly in their immunogenic properties depending on their acylation pattern and bacterial origin. Penta- and tetra-acylated LPS produced by Bacteroides dorei, for example, are antagonistic to TLR4 signaling and actively suppress immune activation. These immunoinhibitory LPS types not only fail to induce endotoxin tolerance but can also block the immune-educational effects of stimulatory LPS, as shown in both in vitro assays and mouse models of autoimmunity. Because our current model does not distinguish between LPS subtypes, it cannot fully capture scenarios in which tolerance is impaired due to insufficient immune stimulation. Future iterations of the model could incorporate functionally distinct LPS categories to explore both ends of the immune education spectrum—from hyperactivation-induced breakdown to under-stimulation-induced failure.

**Alternative Pathways for Immune Imprinting:** Our model focuses on the induction of microbiota-specific IgA via germinal center dynamics within organized mucosal inductive sites, such as Peyer’s patches and colonic patches. These structures directly sample luminal antigens through M cells, are known to support affinity maturation and class switching in response to microbial colonization, and have been shown to be indispensable for generating taxon-specific IgA against commensal bacteria [51,52]​. We do not explicitly incorporate parallel routes of immune activation, such as the goblet cell-associated antigen passage (GAP)–mesenteric lymph node (MLN) axis. This pathway is particularly relevant during pre-weaning, when GAPs facilitate the delivery of luminal antigens to tolerogenic dendritic cells in the lamina propria, which then migrate to MLNs to support regulatory T cell expansion and systemic immune imprinting [53]. While MLNs are capable of supporting germinal center formation [54], their role in microbiota-specific SIgA generation appears to be primarily modulatory. Both PPs and MLNs interact with dendritic cells and B cells, but PPs and colonic patches are more directly involved in the initiation of taxon-specific responses, whereas MLNs contribute to the broader regulation and dissemination of IgA-producing cells [54-56]. These mechanisms, while essential for establishing immune homeostasis, lie outside the specific scope of our current model.

**Gut permeability:** Temporal changes in the tight junctions and permeability of the gut epithelium are also elided in our framing. These factors influence the translocation of antigens, pathogens, and toxins from the gut lumen into the underlying tissues, increasing the risk of pathogen infiltration. The scenario of higher permeability of the gut epithelium during very early stages of ontogeny is partially recapitulated during our sensitivity analysis for the timing of M cell maturation and the initiation of antigenic sampling. Early opening of M cells — akin to compromised tight junctions and increased gut permeability during early ontogeny — allows a higher bacterial antigen burden to be recovered from GALT inductive sites. This elevated antigenic load initiates a more aggressive affinity maturation process against *Enterobacteriaceae*, thus recapitulating an exacerbated immune response against this taxon when gut permeability is compromised. The affinity maturation processes for the symbiotic commensals (*Bifidobacteriaceae*, *Bacteroidaceae*, and *Clostridiales*) are mostly unaffected, demonstrating the strong anti-inflammatory influence of the maternal antibody masking of these bacterial taxa. Similar to these results, implementing the temporal changes in gut permeability into our model is likely to increase the antigenic load recovered from the GALT inductive sites during the very early stages of ontogeny. However, the re-calibration of antigenic sampling rates and affinity maturation parameters such that the endogenous affinity levels still converge to maternal ones will counterweight this temporal bias. This adjustment would likely result in a steeper convergence of the affinity levels without affecting any other outcomes regarding the local antibody response.

**Functional shifts in gut microbiome:** Lastly, strain-specific shifts in the gut microbiome are not represented in our model, yet these shifts are known to affect metabolic and functional development. Our model represents bacterial influences on the microenvironment in a binary fashion (inflammatory or anti-inflammatory), a simplification that yields model tractability, but comes at the expense of generalizing the maturation of the antibody response towards a taxonomic group rather than specific strains. However, formally addressing the known distinct roles of certain bacterial strains in gut immune system development (*e.g.* [16]) and modeling their load and timing of inoculation would enhance our model's ability to address questions specific to the impact of hygiene practices, nutrition, and therapeutic use of probiotics on immune education.

**Impact of unconsidered exogenous variables:** While our model captures primary exogenous inputs that are well-characterized in early immune development, it does not account for other potentially influential factors, such as milk-derived EGF, lipids, viral exposures, or environmental stressors. These variables, though not included in our current model due to lack of robust quantitative data, could affect the immune landscape and modify the predicted outcomes. We suggest that future iterations of the model incorporate these variables, as data becomes available, to provide a more comprehensive analysis of early-life immune dynamics.

***Experimental Model Validation***

Our model is rooted in a mathematical formalization of well-described mechanisms, with the goal of predicting hard-to-intuit outcomes of emergent properties of mechanistic interactions. However, how the different mechanisms are combined in the model may lead to inappropriate weight to different processes, leading to mismatch between model predictions, and empirical outcomes, and this could be interrogated with simple experiments or cohort studies. We provide some plausible experiments below to test the predictions of our model.

**1. Longitudinal cohort study to validate predictive power of fecal samples**

**Prediction:** Our model predicts that the predictive power of SIgA-bound Enterobacteriaceae abundance for downstream immune phenotypes follows a non-monotonic pattern over the course of ontogeny (Fig 3E).

**Approach:** This can be tested in a longitudinal human birth cohort by collecting monthly fecal samples for 16S rRNA sequencing and IgA-seq from birth to two years of age, coupled with clinical follow-up for allergic or inflammatory outcomes. Machine learning models trained on different timepoints could assess how well microbial and SIgA-binding features at each stage predict immune outcomes. This would validate the model’s proposed temporal window of maximal diagnostic value and inform optimal sampling strategies in clinical research.

**2. Probiotic supplementation as an intervention when breastfeeding is not possible**

**Prediction:** Our simulations indicate that in the absence of maternal antibodies, the early introduction of key symbiotic commensals belonging to *Bacteroidaceae* and *Clostridiales* can reduce the pathogenic-to-commensal ratio up to 30-fold in the gut lumen (Fig 5A). This finding supports the use of targeted probiotic supplementation as a practical intervention when breastfeeding is not possible.

**Approach:** In mouse models, neonates receiving exclusive complementary feeding could be administered defined probiotic cocktails, with outcome measures including Enterobacteriaceae abundance in fecal samples and inflammatory biomarkers such as calprotectin. Based on our model predictions (Fig 5A), we would expect a marked reduction in the pathogenic-to-commensal ratio — potentially up to 30-fold — along with a steadily decreasing trend during the first 30 days of life. While the precise magnitude may vary, reproducing this directional shift would lend support to the proposed mechanism and provide a foundation for future translational work. Although further validation in human settings is needed, this experimental design offers a feasible approach to test whether targeted early-life supplementation can reshape gut ecology in accordance with model predictions.

**3. Attenuation of hyperreactivity in offspring of mothers with IBD or allergies**

**Prediction:** Our model predicts that even when maternal SIgA displays hyperreactivity toward commensals—as may occur in mothers with IBD or allergic disorders—the immune system of the offspring does not simply replicate this reactivity, but exhibits an attenuated phenotype (Table 1 and S5 Fig).

**Approach:** Murine models of maternal gut inflammation provide a tractable system to test this prediction. Dams with DSS-induced colitis—a condition known to alter intestinal SIgA reactivity—can be used to generate offspring exposed to hyperreactive maternal antibodies via breastfeeding. Fecal IgA-seq can be performed on both dams and their offspring across developmental timepoints to quantify the similarity of SIgA-binding profiles. Comparing the magnitude of hyperreactivity in maternal versus offspring SIgA responses will allow quantification of attenuation, which can be directly compared with model-predictions in Table 1. Complementary measurements of tolerogenic markers in the gut, such as regulatory T cell abundance and/or cytokine levels in the lamina propria, can further assess whether immune programming remains biased toward tolerance. Experimental confirmation of such attenuation would support the model’s prediction that breastmilk, even from an immunologically dysregulated mother, retains regulatory properties that buffer against the vertical transmission of inflammatory phenotypes.

**4. Knockout experiments to test the significance of M cell sampling bias**

**Prediction:** Our model predicts that the bias in M cell-mediated antigen sampling — the preferential uptake of SIgA-coated bacteria over uncoated ones — is critical for driving the development of immune tolerance (Figs 6 and S6). This bias results in an overrepresentation of masked commensals in antigen presentation (since the neutralized ones will be expulsed from the lumen, they will not be sampled), promoting tolerogenic programming of dendritic cells, particularly via the C-type lectin receptor SIGNR1 [57] and regulatory follicular T cell responses. If this sampling preference is lost, the tolerogenic bias of the GC reactions will also be lost, increasing the risk of hyperreactivity.

**Approach:** While the selective adherence of M cells to SIgA is functionally well-established [58,59], the molecular identity of the responsible receptor remains unknown, making direct genetic knockout experiments unfeasible. However, it is not the sampling bias per se but its downstream effect — the conditioning of dendritic cells in the subepithelial dome — that ultimately drives tolerance. Therefore, our prediction can be tested using SIGNR1 knockout (Signr1–/–) mice, where DCs lack the receptor necessary for tolerogenic response to SIgA [60]. Neonatal mice can be colonized with a defined microbial consortium and provided maternal SIgA via breastfeeding. Comparing fecal IgA-seq profiles from Signr1–/– mice and wild-type controls, one can assess whether the absence of SIGNR1 disrupts the expected development of masking SIgA profiles and tolerance.

***Experimental Model Parametrization***

1. **Characterizing how antibody affinity levels map to bacteria coating rates (Eqns. 1.1.1- 1.1.2):** Differential coating rates of bacteria can be quantified by using IgA-Seq. To mathematically map coating rates to antibody affinity levels (as reflected in the model, Fig 1B), Surface Plasmon Resonance (SPR) [61,62] or Bio-Layer Interferometry (BLI) [63] can be used to quantify the binding affinity of SIgA for bacterial antigens. Using this data for multiple symbiotic and pathogenic strains, one can quantify the function to map affinity levels to masking/neutralizing rates.
2. **Characterizing how the selection threshold (our quantification of T cell help determining the B cell fates) changes during affinity maturation:** The selection threshold in our model is a composite variable that reflects the outcome of multiple selection processes occurring within the germinal centers. This approach aligns with the common practice in mathematical modeling of pooling and abstracting detailed mechanisms into higher-level parameters. A selection bias toward an increased BCR affinity range when Tfh:Tfr ratio [64,65] and/or the inflammatory tone of the microenvironment [66] is increasing is well established in the literature. However, the mathematical nature of this relationship is assumed to be multiplicative in our model (Eqn. 1.2.8), with the addition of a factor to account for their differences in magnitude (Materials and Methods, section Immune Dynamics*)*. Quantifying the nature of this relationship (additive, multiplicative, non-linear) is challenging, but several experimental approaches can be designed to investigate this hypothesis:
   1. ***In vitro* germinal center reactions:** Organoid cultures or germinal center-like structures *in vitro* can be used, where one can control the levels of cytokines, chemokines, and T-cell interactions. With established protocols for *in vitro* germinal center B cell culture, we can elucidate the molecular mechanisms of GC B cell differentiation [67,68]. By varying levels of cytokines (e.g., IL-4, IL-21) and T-cell help (e.g., by adding Tfh cells or anti-CD40 antibodies), isolating B cells and using Surface Plasmon Resonance (SPR) to quantify their binding affinity at different time points, one can curate a dataset where the longitudinal relationship between the affinity levels, T cell help, and microenvironmental effects can be quantified.
   2. **Animal models with conditional knockouts:** Similar to *in vitro* methods, we can apply conditional knockout mouse models of cytokine receptors (e.g. IL-21R) [69] or T-cell help (e.g. CD40-CD40L interactions) [70,71], isolate plasma cells from lamina propria at different time points during ontogeny, quantify the B-cell binding affinity as described in point a., and quantify the relationship.
   3. ***Ex Vivo* Lymphoid Tissue Cultures:** *Ex vivo* engineered organotypic cultures have enabled the real-time study and control of biological functioning of mammalian tissues. A B cell follicle organoid made of nanocomposite biomaterials recapitulating the anatomical microenvironment of a lymphoid tissue that provides the basis to induce accelerated germinal center reactions has been already demonstrated in the literature [72,73]. Thus, another option is to culture explanted lymphoid tissues and manipulate the levels of microenvironmental factors (e.g., through cytokine treatments) and T-cell help (e.g., through anti-CD40 or anti-ICOS treatments), isolate the plasma cells at different time points, quantify the affinity as described in point a., and quantify the relationship.
3. **Quantifying the proliferation, somatic hypermutation, and selection (P-SHM-S) cycle:** To measure which B cells with specific BCR affinities are selected, and which have died during proliferation, somatic hypermutation (SHM), and selection in germinal centers (GCs), several methods can be employed.
   1. One can utilize single-cell RNA sequencing (scRNA-seq) as demonstrated by Corinaldesi *et al.* [74]. Their study used scRNA-seq to track immunoglobulin repertoire and transcriptomic changes in germinal center B cells, providing detailed insights into the selection and differentiation of B cells based on affinity maturation​​. This method allows for the precise characterization of B cell populations and their affinity profiles, which can be used to quantify how the distribution of BCR affinities change over the course of P-SHM-S cycles in our model.
   2. Another alternative is to use EdU incorporation assays, as described by Biram and Shulman [75]. Their study demonstrates the use of EdU labeling to measure B cell proliferation *in vivo*, providing a detailed methodology for tracking B cell proliferation and selection within germinal centers using flow cytometry​​. This technique can quantify the impact of BCR affinity on cell survival and proliferation, which can be used to quantify the selection range for different B cell fates (points 1-7 under Modeling the role of Germinal Centers, Materials and Methods) that is used in our model.

References

1. Tsukuda N, Yahagi K, Hara T, Watanabe Y, Matsumoto H, Mori H, et al. Key bacterial taxa and metabolic pathways affecting gut short-chain fatty acid profiles in early life. ISME J. 2021;15: 2574–2590. doi:10.1038/s41396-021-00937-7

2. Palmer C, Bik EM, DiGiulio DB, Relman DA, Brown PO. Development of the human infant intestinal microbiota. PLoS Biol. 2007;5: e177.

3. Planer JD, Peng Y, Kau AL, Blanton LV, Ndao IM, Tarr PI, et al. Development of the gut microbiota and mucosal IgA responses in twins and gnotobiotic mice. Nature. 2016;534: 263–266. doi:10.1038/nature17940

4. Pan K. The Effects of Feeding Type on the Gut Microbiota of Neonates and Early Infants. J Infect Dis Case Rep. 2022; 1–5. doi:10.47363/JIDSCR/2022(3)159

5. van der Waaij LA, Kroese FG, Visser A, Nelis GF, Westerveld BD, Jansen PL, et al. Immunoglobulin coating of faecal bacteria in inflammatory bowel disease. Eur J Gastroenterol Hepatol. 2004;16: 669–674. doi:10.1097/01.meg.0000108346.41221.19

6. Tellier J, Nutt SL. The secret to longevity, plasma cell style. Nat Immunol. 2022;23: 1507–1508. doi:10.1038/s41590-022-01340-w

7. Brandtzaeg P. Mucosal immunity: integration between mother and the breast-fed infant. Vaccine. 2003;21: 3382–3388.

8. Wernroth M-L, Peura S, Hedman AM, Hetty S, Vicenzi S, Kennedy B, et al. Development of gut microbiota during the first 2 years of life. Sci Rep. 2022;12: 9080.

9. Pop M, Walker AW, Paulson J, Lindsay B, Antonio M, Hossain MA, et al. Diarrhea in young children from low-income countries leads to large-scale alterations in intestinal microbiota composition. Genome Biol. 2014;15: 1–12.

10. Favre L, Spertini F, Corthésy B. Secretory IgA Possesses Intrinsic Modulatory Properties Stimulating Mucosal and Systemic Immune Responses. J Immunol. 2005;175: 2793–2800. doi:10.4049/jimmunol.175.5.2793

11. Morbach H, Eichhorn EM, Liese JG, Girschick HJ. Reference values for B cell subpopulations from infancy to adulthood. Clin Exp Immunol. 2010;162: 271–279. doi:10.1111/j.1365-2249.2010.04206.x

12. Cashman KS, Jenks SA, Woodruff MC, Tomar D, Tipton CM, Scharer CD, et al. Understanding and measuring human B‐cell tolerance and its breakdown in autoimmune disease. Immunol Rev. 2019;292: 76–89. doi:10.1111/imr.12820

13. Davis CL, Wahid R, Toapanta FR, Simon JK, Sztein MB, Levy D. Applying Mathematical Tools to Accelerate Vaccine Development: Modeling Shigella Immune Dynamics. PLoS ONE. 2013;8: e59465. doi:10.1371/journal.pone.0059465

14. Siegrist C-A, Aspinall R. B-cell responses to vaccination at the extremes of age. Nat Rev Immunol. 2009;9: 185–194. doi:10.1038/nri2508

15. Silverstein RB, Mysorekar IU. Group therapy on in utero colonization: seeking common truths and a way forward. Microbiome. 2021;9. doi:10.1186/s40168-020-00968-w

16. Xiao L, Zhao F. Microbial transmission, colonisation and succession: from pregnancy to infancy. Gut. 2023;72: 772–786. doi:10.1136/gutjnl-2022-328970

17. Mörbe UM, Jørgensen PB, Fenton TM, von Burg N, Riis LB, Spencer J, et al. Human gut-associated lymphoid tissues (GALT); diversity, structure, and function. Mucosal Immunol. 2021;14: 793–802. doi:10.1038/s41385-021-00389-4

18. Owen RL. Uptake and transport of intestinal macromolecules and microorganisms by M cells in Peyer’s patches—a personal and historical perspective. Elsevier; 1999. pp. 157–163.

19. Agace WW, McCoy KD. Regionalized Development and Maintenance of the Intestinal Adaptive Immune Landscape. Immunity. 2017;46: 532–548. doi:10.1016/j.immuni.2017.04.004

20. Smith P, MacDonald T, Blumberg R. Society for Mucosal Immunology. Principles of mucosal immunology. London: Garland Science. 2013.

21. Baptista A, Olivier B, Goverse G, Greuter M, Knippenberg M, Kusser K, et al. Colonic patch and colonic SILT development are independent and differentially regulated events. Mucosal Immunol. 2013;6: 511–521.

22. Buettner M, Lochner M. Development and function of secondary and tertiary lymphoid organs in the small intestine and the colon. Front Immunol. 2016;7: 342.

23. Chang S-Y, Cha H-R, Uematsu S, Akira S, Igarashi O, Kiyono H, et al. Colonic patches direct the cross-talk between systemic compartments and large intestine independently of innate immunity. J Immunol. 2008;180: 1609–1618.

24. Owen RL, Piazza AJ, Ermak TH. Ultrastructural and cytoarchitectural features of lymphoreticular organs in the colon and rectum of adult BALB/c mice. Am J Anat. 1991;190: 10–18.

25. Miller H, Zhang J, KuoLee R, Patel GB, Chen W. Intestinal M cells: the fallible sentinels? World J Gastroenterol WJG. 2007;13: 1477.

26. Shu J-X, Zhong C-S, Shi Z-J, Zeng B, Xu L-H, Ye J-Z, et al. Berberine augments hypertrophy of colonic patches in mice with intraperitoneal bacterial infection. Int Immunopharmacol. 2021;90: 107242.

27. Dohi T, Rennert PD, Fujihashi K, Kiyono H, Shirai Y, Kawamura YI, et al. Elimination of colonic patches with lymphotoxin β receptor-Ig prevents Th2 cell-type colitis. J Immunol. 2001;167: 2781–2790.

28. Lycke NY, Bemark M. The role of Peyer’s patches in synchronizing gut IgA responses. Front Immunol. 2012;3. doi:10.3389/fimmu.2012.00329

29. Gibbons D, Spencer J. Mouse and human intestinal immunity: same ballpark, different players; different rules, same score. Mucosal Immunol. 2011;4: 148–157.

30. Barone F, Patel P, Sanderson J, Spencer J. Gut-associated lymphoid tissue contains the molecular machinery to support T-cell-dependent and T-cell-independent class switch recombination. Mucosal Immunol. 2009;2: 495–503. doi:10.1038/mi.2009.106

31. Knoop KA, Newberry RD. Isolated Lymphoid Follicles are Dynamic Reservoirs for the Induction of Intestinal IgA. Front Immunol. 2012;3. doi:10.3389/fimmu.2012.00084

32. Sollid LM, Iversen R. Tango of B cells with T cells in the making of secretory antibodies to gut bacteria. Nat Rev Gastroenterol Hepatol. 2022;20: 120–128. doi:10.1038/s41575-022-00674-y

33. Nowosad CR, Mesin L, Castro TBR, Wichmann C, Donaldson GP, Araki T, et al. Tunable dynamics of B cell selection in gut germinal centres. Nature. 2020;588: 321–326. doi:10.1038/s41586-020-2865-9

34. Vergani S, Muleta KG, Da Silva C, Doyle A, Kristiansen TA, Sodini S, et al. A self-sustaining layer of early-life-origin B cells drives steady-state IgA responses in the adult gut. Immunity. 2022;55: 1829-1842.e6. doi:10.1016/j.immuni.2022.08.018

35. Zhao Q, Elson CO. Adaptive immune education by gut microbiota antigens. Immunology. 2018;154: 28–37. doi:10.1111/imm.12896

36. Ng KW, Hobbs A, Wichmann C, Victora GD, Donaldson GP. B cell responses to the gut microbiota. Advances in Immunology. Elsevier; 2022. pp. 95–131. doi:10.1016/bs.ai.2022.08.003

37. Jackson MA, Pearson C, Ilott NE, Huus KE, Hegazy AN, Webber J, et al. Accurate identification and quantification of commensal microbiota bound by host immunoglobulins. Microbiome. 2021;9: 1–22.

38. Qian G, Mahdi A. Sensitivity analysis methods in the biomedical sciences. Math Biosci. 2020;323: 108306.

39. Pabst O, Cerovic V, Hornef M. Secretory IgA in the Coordination of Establishment and Maintenance of the Microbiota. Trends Immunol. 2016;37: 287–296. doi:10.1016/j.it.2016.03.002

40. Fransen F, Zagato E, Mazzini E, Fosso B, Manzari C, El Aidy S, et al. BALB/c and C57BL/6 Mice Differ in Polyreactive IgA Abundance, which Impacts the Generation of Antigen-Specific IgA and Microbiota Diversity. Immunity. 2015;43: 527–540. doi:10.1016/j.immuni.2015.08.011

41. Liu Y, Rhoads J. Communication between B-Cells and Microbiota for the Maintenance of Intestinal Homeostasis. Antibodies. 2013;2: 535–553. doi:10.3390/antib2040535

42. Olivares M, Walker AW, Capilla A, Benítez-Páez A, Palau F, Parkhill J, et al. Gut microbiota trajectory in early life may predict development of celiac disease. Microbiome. 2018;6: 1–11.

43. Semmes EC, Chen J-L, Goswami R, Burt TD, Permar SR, Fouda GG. Understanding Early-Life Adaptive Immunity to Guide Interventions for Pediatric Health. Front Immunol. 2021;11. doi:10.3389/fimmu.2020.595297

44. Spencer J, Sollid LM. The human intestinal B-cell response. Mucosal Immunol. 2016;9: 1113–1124.

45. Gullberg E, Keita ÅV, Sa’ad YS, Andersson M, Caldwell KD, Söderholm JD, et al. Identification of cell adhesion molecules in the human follicle-associated epithelium that improve nanoparticle uptake into the Peyer’s patches. J Pharmacol Exp Ther. 2006;319: 632–639.

46. Hase K, Kawano K, Nochi T, Pontes GS, Fukuda S, Ebisawa M, et al. Uptake through glycoprotein 2 of FimH+ bacteria by M cells initiates mucosal immune response. Nature. 2009;462: 226–230.

47. Rollenske T, Burkhalter S, Muerner L, von Gunten S, Lukasiewicz J, Wardemann H, et al. Parallelism of intestinal secretory IgA shapes functional microbial fitness. Nature. 2021;598: 657–661.

48. Goenka A, Kollmann TR. Development of immunity in early life. J Infect. 2015;71: S112–S120.

49. Pabst O, Slack E. IgA and the intestinal microbiota: the importance of being specific. Mucosal Immunol. 2020;13: 12–21. doi:10.1038/s41385-019-0227-4

50. Vatanen T, Kostic AD, d’Hennezel E, Siljander H, Franzosa EA, Yassour M, et al. Variation in microbiome LPS immunogenicity contributes to autoimmunity in humans. Cell. 2016;165: 842–853.

51. Hahn A, Thiessen N, Pabst R, Buettner M, Bode U. Mesenteric lymph nodes are not required for an intestinal immunoglobulin A response to oral cholera toxin. Immunology. 2010;129: 427–436.

52. Rios D, Wood M, Li J, Chassaing B, Gewirtz A and, Williams I. Antigen sampling by intestinal M cells is the principal pathway initiating mucosal IgA production to commensal enteric bacteria. Mucosal Immunol. 2016;9: 907–916.

53. Knoop KA, Gustafsson JK, McDonald KG, Kulkarni DH, Coughlin PE, McCrate S, et al. Microbial antigen encounter during a preweaning interval is critical for tolerance to gut bacteria. Sci Immunol. 2017;2. doi:10.1126/sciimmunol.aao1314

54. Li C, Lam E, Perez-Shibayama C, Ward LA, Zhang J, Lee D, et al. Early-life programming of mesenteric lymph node stromal cell identity by the lymphotoxin pathway regulates adult mucosal immunity. Sci Immunol. 2019;4: eaax1027.

55. Hashizume-Takizawa T, Shibata N, Kurashima Y, Kiyono H, Kurita-Ochiai T, Fujihashi K. Distinct roles for Peyer’s patch B cells for induction of antigen-specific IgA antibody responses in mice administered oral recombinant Salmonella. Int Immunol. 2019;31: 531–541.

56. Hashizume-Takizawa T, Kobayashi R, Tsuzukibashi O, Saito M, Kurita-Ochiai T. CCR7-deficient mice exhibit a delayed antigen-specific mucosal IgA antibody response to an oral recombinant Salmonella strain. Pathog Dis. 2019;77: ftz024.

57. Diana J, Moura IC, Vaugier C, Gestin A, Tissandie E, Beaudoin L, et al. Secretory IgA induces tolerogenic dendritic cells through SIGNR1 dampening autoimmunity in mice. J Immunol. 2013;191: 2335–2343.

58. Mantis NJ, Cheung MC, Chintalacharuvu KR, Rey J, Corthésy B, Neutra MR. Selective adherence of IgA to murine Peyer’s patch M cells: evidence for a novel IgA receptor. J Immunol. 2002;169: 1844–1851.

59. Rol N, Favre L, Benyacoub J, Corthésy B. The role of secretory immunoglobulin A in the natural sensing of commensal bacteria by mouse Peyer’s patch dendritic cells. J Biol Chem. 2012;287: 40074–40082.

60. Wieland CW, Koppel EA, den Dunnen J, Florquin S, McKenzie AN, van Kooyk Y, et al. Mice lacking SIGNR1 have stronger T helper 1 responses to Mycobacterium tuberculosis. Microbes Infect. 2007;9: 134–141.

61. Chardin H, Mercier K, Frydman C, Vollmer N. Surface plasmon resonance imaging: a method to measure the affinity of the antibodies in allergy diagnosis. J Immunol Methods. 2014;405: 23–28.

62. Schasfoort RB, van Weperen J, van Amsterdam M, Parisot J, Hendriks J, Koerselman M, et al. Presence and strength of binding of IgM, IgG and IgA antibodies against SARS-CoV-2 during COVID-19 infection. Biosens Bioelectron. 2021;183: 113165.

63. Noy-Porat T, Alcalay R, Mechaly A, Peretz E, Makdasi E, Rosenfeld R, et al. Characterization of antibody-antigen interactions using biolayer interferometry. STAR Protoc. 2021;2: 100836.

64. Huang Y, Chen Z, Wang H, Ba X, Shen P, Lin W, et al. Follicular regulatory T cells: a novel target for immunotherapy? Clin Transl Immunol. 2020;9: e1106.

65. Wing JB, Ise W, Kurosaki T, Sakaguchi S. Regulatory T cells control antigen-specific expansion of Tfh cell number and humoral immune responses via the coreceptor CTLA-4. Immunity. 2014;41: 1013–1025.

66. Zotos D, Coquet JM, Zhang Y, Light A, D’Costa K, Kallies A, et al. IL-21 regulates germinal center B cell differentiation and proliferation through a B cell–intrinsic mechanism. J Exp Med. 2010;207: 365–378.

67. El Shikh MEM, El Sayed R, Aly NAR, Prediletto E, Hands R, Fossati-Jimack L, et al. Follicular dendritic cell differentiation is associated with distinct synovial pathotype signatures in rheumatoid arthritis. Front Med. 2022;9: 1013660.

68. Haniuda K, Nojima T, Kitamura D. In vitro-induced germinal center b cell culture system. Germinal Cent Methods Protoc. 2017; 125–133.

69. Huang X, Yang W, Yao S, Bilotta AJ, Lu Y, Zhou Z, et al. IL-21 promotes intestinal memory IgA responses. J Immunol. 2020;205: 1944–1952.

70. Lei X-F, Ohkawara Y, Stämpfli MR, Mastruzzo C, Marr RA, Snider D, et al. Disruption of antigen-induced inflammatory responses in CD40 ligand knockout mice. J Clin Invest. 1998;101: 1342–1353.

71. Xu J, Foy TM, Laman JD, Elliott EA, Dunn JJ, Waldschmidt TJ, et al. Mice deficient for the CD40 ligand. Immunity. 1994;1: 423–431.

72. Purwada A, Shah SB, Béguelin W, August A, Melnick AM, Singh A. Ex vivo synthetic immune tissues with T cell signals for differentiating antigen-specific, high affinity germinal center B cells. Biomaterials. 2019;198: 27–36.

73. Purwada A, Jaiswal MK, Ahn H, Nojima T, Kitamura D, Gaharwar AK, et al. Ex vivo engineered immune organoids for controlled germinal center reactions. Biomaterials. 2015;63: 24–34.

74. Corinaldesi C, Holmes AB, Shen Q, Grunstein E, Pasqualucci L, Dalla-Favera R, et al. Tracking immunoglobulin repertoire and transcriptomic changes in germinal center B cells by single-cell analysis. Front Immunol. 2022;12: 818758.

75. Biram A, Shulman Z. Evaluation of B cell proliferation in vivo by EdU incorporation assay. Bio-Protoc. 2020;10: e3602–e3602.
