# Supplementary material for: A model of early-life interactions between the gut microbiome and adaptive immunity provides insights into the ontogeny of immune tolerance
Source: PLoS Biol. 2025 Aug 14;23(8):e3003263. doi: 10.1371/journal.pbio.3003263 (PMC12352683; doi:10.1371/journal.pbio.3003263)
Supplement: Attachment — Submitted filename: PLOS BIO RESPONSE TO REVIEWERS.pdf [file pbio.3003263.s020.pdf]

Dear Dr Tepekule,

Thank you for your patience while your manuscript "The ontogeny of immune tolerance: a model of early-life secretory IgA - gut microbiome interactions" was peer-reviewed at PLOS Biology. Your study has now been assessed and discussed by the PLOS Biology editors, an Academic Editor with relevant expertise and several independent reviewers. As you will see in their reports at the end of this email, despite noting the interest of the study, the reviewers raise significant concerns about the conclusiveness of the work and assumptions of the model.

Based on these reviews, I regret that we will not be able to invite revision of the study, as successfully addressing the concerns raised would entail a significant amount of work (beyond what could be reasonably expected of a revision) and whether the current conclusions would be supported is unclear.

All three reviewers (commented) about the potential of the model. However, all reviewers and the Academic Editor are concerned about the assumptions made and how difficult the study was to read. Reviewer 1 has several critiques like the complexity of the model which makes it hard to evaluate what is contributing to the results, that the model might be overfit since there is no experimental validation (the major critique), and the lack of accessibility to non-technical experts. The reviewer also mentioned that there is no consideration for early life infections and memory phenotypes within the model. Reviewer 2 mentions that the model does not consider formula-fed infants and you should provide more information on how the input data was collated. Reviewer 3 thinks the model could be useful but raises some concerns that should either be incorporated in the model or at least discussed.

After discussion with the reviewers and the Academic Editor, we believe that the study needs some experimental validation and restructuring to improve readability, as well as to incorporate the significant limitations. While we understand that not all parameters can be considered in the model, because producing the required data would require substantial cost and effort with an uncertain outcome, we cannot in good faith invite revision of your study. However, given our and the reviewers' interest in the topic, if further work were to allow you to comprehensively address the reviewer concerns outlined above, we would be willing to consider an extensively revised manuscript as a re-submission. The article would receive a new number and submission date and we would evaluate it in view of any related work published in the interim, but we would make every attempt to engage the same Academic Editor and reviewers in its assessment, if no novelty concerns arise.

We appreciate that the scale of the requested additional work is significant and understand that you may prefer to pursue faster publication of this work elsewhere. At this point, your manuscript is no longer under active consideration at PLOS Biology. We might be able to facilitate a faster consideration at another PLOS journal; if this is of interest to you, please let me know.

Please feel free to contact me if you would like to discuss next steps for your manuscript. If you do make the choice to revise and re-submit to PLOS Biology, please provide a point-by-point response to the reviewers' comments when re-submitting. You should include this as a separate file, labelled 'Response to Reviewers'. Please also provide the tracking number of this manuscript [PBIOLOGY-D-24-03629R1] in the 'previous interaction' section of the online submission form and in your cover letter.

I am sorry that we cannot be more positive on this occasion. I thank you for having considered PLOS Biology for publication and hope that you will find the reviewers' feedback helpful as you consider how to proceed with this work.

Sincerely,
Melissa

Melissa Vazquez Hernandez
Associate Editor
PLOS Biology

mvazquezhernandez@plos.org

We would like to thank the editor and reviewers for their insightful and constructive feedback on our manuscript. We have taken the concerns seriously and implemented a wide range of substantial revisions, which we believe significantly improve the manuscript's clarity, biological grounding, and more effectively describe how it contributes to the field. Below we briefly summarize the key changes:

1. **Model complexity and interpretability:** We included a detailed summary table (Table S6) explaining the biological significance and mechanistic impact of the highest-ranked parameters from our global sensitivity analysis. To improve interpretability, we revised the discussion section to highlight the clinical relevance and novelty of our model predictions, and revised Figure 1A and 1B alongside the detailed figure captions.

2. **Motivation, validation, and biological plausibility:** We incorporated a new external validation using independent cohort data from Olivares *et al.* (2018). Our model successfully replicated taxonomic predictor importance rankings and distinguished tolerant versus hyperreactive phenotypes using cross-validated AUC metrics, demonstrating strong alignment with experimental data. Ideally, a more comprehensive set of experiments would have strengthened our findings, but such extensive animal studies and/or longitudinal cohort studies would require substantial resources beyond the scope of the current work. Nevertheless, we revisited the manuscript to more clearly communicate our core motivation of laying the foundations for defining 'immune education'. We explicitly acknowledge that the underlying assumptions, model structure, and predicted outcomes remain open to refinement through future experimental studies and empirical testing, now detailing a range of experiments to probe model validity.

3. **Immunological memory:** We added simulations showing that our model broadly captures memory responses by implicitly modeling plasma cell longevity and replenishment. These simulations are built around known features of plasma cell and memory cell demography and demonstrate that immunological memory can persist long after the extinction of a microbial taxon, directly addressing concerns about the relevance of antibody profiles to past exposures.

4. **Early-life infections and dysbiosis:** We added simulations of early-life dysbiosis by artificially promoting *Enterobacteriaceae* growth, and showed how this leads to broader hyperreactivity later in life. This scenario illustrates how early-life infections can shape long-term immune trajectories, directly responding to reviewer concerns.

5. **Biological assumptions and anatomical context:** We clarified our anatomical focus (colonic vs. small intestine), updated terminology throughout the manuscript to reflect the broader GALT context, and added discussion of SIgA cross-reactivity and alternative immune activation pathways (e.g., GAP–MLN axis).

6. **Maternal cytokines, M cell maturation, and diversity of LPS structures:** We revised the manuscript to address the implications of the exclusion of maternal cytokine transfer, the diversity of LPS structures, and the uncertain timing of M cell maturation in humans. For the latter point, we referred to our sensitivity analysis to address the uncertainty of M cell activation time. For the prior point, we also included new simulations to show how tolerance is impaired when inflammatory LPS by *Enterobacteriaceae* was absent from the gut, consistent with Vatanen *et al* (2016).

7. **Reformulation of SIgA functionality:** We replaced the binary masking/neutralizing assumption with a continuous function of both activities based on affinity. We re-fitted the model under this new framework and updated all relevant figures (e.g., Fig. 2B, 3A–B, and 6).

8. **Feeding scenarios and data transparency:** We clarified how formula-fed infants were included in the inference process and made all curated input datasets (relative abundances, IgA-seq, metadata) along

with the preprocessing scripts publicly available via a GitHub repository to enhance transparency and reproducibility.

9. **Accessibility and figure clarity:** We revised most main figures to improve clarity and accessibility, including simplified color schemes, updated labels, and more informative figure legends tailored to both technical and non-technical audiences.

We believe these revisions have meaningfully addressed the reviewers' main concerns and significantly enhanced the manuscript's scientific rigor, biological realism, and accessibility. We hope our revised manuscript is suitable for publication, and we are grateful for the opportunity to resubmit our work.

REVIEWERS' COMMENTS:

Reviewer #1:

The paper presents a model of early life development of the sIgA compartment in newborns during the first two years of life in an attempt to shed light on how 'immune education' in the gut takes place. Using several recently published datasets, the authors develop a highly detailed mathematical model of the interplay between gut bacteria and the developing immune system of the newborn baby.

The authors use data from several recently published papers on the microbiome and sIgA responses during the first two years of life to estimate model parameters using two major assumptions:
1. The infant sIgA resembles that of their mother.
2. The repertoire generally stabilizes at two years of age.

Four major bacterial species are selected as a simplified model of the gut microbiome.

Secretory IgA is characterized into two categories: masking and neutralizing, thereby defining three subpopulations of bacteria - uncoated, masked, and neutralized. Masked and neutralized bacteria can be estimated from IgA-seq and BugFACS data. IgA affinity is assumed to be linked to its masking vs. neutralizing roles.

Relative bacterial abundance and IgA-seq data derived from infants' fecal samples are used for model parameterization and are used to eventually reconstruct relative abundances from the feces, which are then used to estimate relative abundances in the gut. This part of the model is validated using data from Planer et al. [21].

The model is then used to estimate immune phenotypes from the abundance of IgA-coated Enterobacteriaceae from specific ontogeny stages. Specifically, they show that in most cases the immune phenotypes converge to tolerogenic phenotypes in over 90% of the simulations of different feeding scenarios, but only from data early after birth or later following stabilization.

The paper then moves to address what factors may contribute to the immune system after weaning, even after some of the core cell types are no longer present in the child. Specifically, they investigate the relationship between the duration of EBF (exclusive breastfeeding) on affinity maturation of antibodies. The model assumes that extended EBF delays M cell maturation and therefore postpones the maturation of T-cells and B-cells. They simulate different EBF and MF (mixed feeding) duration periods and their effects on pre-weaning and post-weaning commensals. Their simulations show that the effects on the two populations are different using a feature importance framework. In line with the hypothesis, they present that both EBF and MF durations contribute to the variance in the eSigIgA affinities to post-weaning commensals, with a more prominent role for MF duration. If EBF is followed by ECF (exclusive complementary feeding) directly, developing tolerance to post-weaning commensals is reduced. The paper uses a random forest approach to claim that the effects of

EBF post-weaning are mostly driven by microenvironmental stimulation rather than taxonomic composition — i.e., that the conditions and signals within the child's gut are critical for shaping the infant's response to the presence and abundance of specific taxa. This is an interesting finding, which may be challenging to study experimentally.

They then use work by Ramanan et al., which demonstrated that in mice, immune priming of the newborn is mostly mediated by maternal sIgA, which could be biased toward more tolerogenic or inflammatory phenotypes. They compared five different feeding scenarios coupled with varying maternal immunological profiles and showed that in all but the control group, there was cross-generational transmission of a pro-inflammatory phenotype, suggesting a prominent role for sIgA in immune imprinting. The scenarios differed by the level of pathogenic control, with ECF + probiotics and sIgA-deficient feeding leading to a transient increase in the pathogenic-to-symbiotic ratio followed by a decrease, which increased the risk of NEC. An IBD-like phenotype showed the worst outcome in the infant despite the abundance of neutralizing antibodies against Enterobacteriaceae.

Overall, it is clear the authors have made an effort to make the paper accessible to the non-modeling crowd, but even as a computationally trained scientist, I struggled to read this paper. My major critiques are the following:

1. The models developed in this paper are very complex and involve a broad range of cells, compartments, and bacteria. They also rely on several key assumptions or hypotheses that the authors deduce from recently published experimental studies. There are so many parameters and equations that are fit that it is extremely difficult to assess how and which of them contribute to the findings reported in the paper. I was not able to follow the math in detail, and it certainly seems to me that someone should, but this may be a highly difficult task given the complexity of this model.

We acknowledge the model's complexity. As noted in the reviewer's subsequent comments, the current level of complexity still required us to make simplifications, such as the exclusion of memory B cells, a set of limitations discussed in detail in our model limitations section. We believe that adequately capturing the microbiome-IgA relationships requires at minimum the level of complexity present in our model. However, we understand the challenge of interpreting the effects of parameters and their role in the model outcomes. To address concerns about model interpretability, we have included a Table S6 in the Supplementary Material (lines 1969-1974) in the revised version, also included below). This table explains the highest ranked parameters (above Q50, Fig. S6) in our global sensitivity analysis in detail and discusses their impact on model outcomes, thus providing a more directly accessible interpretation of the fundamental model dynamics.

Table S6. Interpretation of the highest-ranked parameters in global sensitivity analysis and their impact on model dynamics. This table presents parameters ranking above the 50th percentile (Q50) in our global sensitivity analysis (Fig. S6). For each parameter, we provide its biological interpretation, functional role within the model, and its quantitative influence on model outcomes.

**Notation**	**Description**	**Interpretation**
$\epsilon^{m}/\epsilon^{uc}$	The ratio of the antigenic-sampling rate of masked and uncoated bacterial antigens by M cells.	This ratio represents the selective bias of M cells for sampling IgA-bacteria complexes. As $\epsilon^{m}/\epsilon^{uc}$ increases, it promotes higher accumulation of IgA-coated bacterial antigens relative to uncoated ones in the GALT inductive sites. This leads to the relatively higher activation of tolerogenic dendritic cells compared to inflammatory dendritic cells, and consequently leading to a lower Tfh:Tfr ratio. This ratio, proxied by the ratio of uncoated to total antigens accumulating in the inductive sites in Eqn. S1.2.8, directly influences the selection threshold parameter δ_i that governs the scale of affinity maturation, thus directly influencing the development of endogenous affinity levels. Note that the effect of $\epsilon^{m}/\epsilon^{uc}$ is not taxon-specific. A detailed analysis of the impact of $\epsilon^{m}/\epsilon^{uc}$ is presented in Fig. 6.
τ^{δ}	Multiplier to adjust the incremental increase in	This parameter directly influences the selection threshold value δ_i (Fig. 2C).

	the selection threshold calculated in Eqn. S1.2.8.	Biologically, it represents how “stringent” the immunological signals (such as Tfh:Tfr cell ratio and the inflammation level) in the germinal center environment are in selecting B cells based on their receptor affinity. Higher values of τ^{δ} indicates the immune system is exerting stronger selection — allowing B cells with higher affinity for antigen receive sufficient help to survive and proliferate–, leading to faster increases in BCR affinity per P-SHM-S cycle. In our model, this parameter is fixed and assumed to depend on the individual's intrinsic efficiency of germinal center reactions.<br><br>This parameter can vary based on genetic factors affecting costimulatory molecule expression levels, T-B cell interaction quality, and the architecture of germinal center microenvironments..This parameter can be inferred from experiments that track the distribution of BCR affinities over multiple time points in controlled germinal center reactions where all other variables (antigen availability, inflammatory cytokines, initial BCR affinity distribution, and Tfh:Tfr ratio) are standardized, potentially through immunization experiments. Single-cell technologies tracking the fate of individual B cell clones combined with mathematical modeling could estimate this parameter by fitting observed affinity trajectories. Note that this parameter represents an intrinsic efficiency rate of the GC reactions, meaning that it is not taxon-specific.
C_n	Amplitude of the exponential function describing the diminishing pool of naïve T and B cells.	This parameter is a proxy for the initial size of the naïve T and B cell pool. As C_n increases (decreases), there will be more (less) naïve B cells migrating from the bone marrow to the GALT inductive site, being activated by bacterial antigens, and turning into circulating GC B cells at each round of proliferation, somatic hypermutation, and selection (P-SHM-S) cycle. A higher influx of newly activated cells broadens the BCR affinity distribution of the circulating GC B cells – effectively introducing lower-affinity clones into the pool – which reduces the average BCR affinity falling within the selection thresholds, thus lowering the average endogenous SIgA (eSIgA) affinity. Conversely, if C_n becomes too low (approaching 0), the number of cells available to differentiate into circulating or plasma cells may be insufficient, leading to negligible eSIgA secreting plasma cells. However, such extreme cases were excluded from our sensitivity analysis; the range for C_n was chosen to ensure that the lower bound still supports circulating and plasma cell differentiation. Since the abundance of bacteria is regulated by eSIgA concentration and affinities, which in turn regulates the immunostimulatory tone of the microenvironment, value of C_n influences the affinity maturation process through ecological and inflammatory feedback loops.
c_n	Decay rate of the exponential function describing the diminishing pool of naïve T and B cells.	This parameter determines the rate at which naïve B and T cells become activated and differentiate—naïve B cells joining the GC reactions and naïve T cells differentiating into effector subsets such as Tfh or Tfr cells. As c_n increases (decreases), naïve cells will be depleted faster (slower), leading to a faster (slower) convergence of the BCR affinity values of the endogenous plasma cells. A faster convergence means that the host will exhaust its supply of naïve cells earlier, prematurely ending GC reactions and affecting the final BCR affinity values of endogenous plasma cells. With slower convergence, naïve cells remain available longer, allowing GC reactions to continue and potentially achieve different BCR affinity outcomes.
th_{range}	The plasma cell differentiation range, where $th_{high} = 1 - th_{range}$ and $th_{ang} = 1 + th_{range}$.	This parameter defines the relative width of the affinity window around the selection threshold (δ_i) that leads to plasma cell differentiation (Fig. 1C). When set to a value of 0.2 (20%), for example, cells with BCR affinities between 80% and 120% of the threshold value will differentiate into plasma cells. Larger values create a more permissive selection process, while smaller values enforce stricter selection pressure on the circulating B cells. Therefore, this parameter affects both the range of BCR affinity and the number of circulating B cells turning to plasma cells at each round of proliferation, somatic hypermutation, and selection (P-SHM-S) cycle.

α_i	Relative invasiveness of taxon i.	This parameter defines the taxon's ability to penetrate intestinal epithelial cells. As this value increases, the SIgA-**uncoated** bacterial load in the GALT inductive sites increases, which in turn increases the converged endogenous affinity level against the taxon. To reduce the impact of identifiability problems during inference, we fixed the invasiveness of *Enterobacteriaceae* to 1 ($\alpha_E = 1$) and inferred the invasiveness of the symbiotic commensals *Bifidobacteriaceae*, *Bacteroidaceae*, and *Clostridiales* relative to the pathogenic taxon *Enterobacteriaceae*. Value and effects of α_i are taxon-specific, meaning that modulating α_i directly impacts the affinity maturation against taxon i via increasing the cumulative uncoated to total bacterial antigen ratio in Eqn. S1.2.8. However, since altering the endogenous affinity against a specific taxonomic group affects not only its own abundance but also the abundance of other taxa through community interactions, it can produce broader indirect effects.
τ^c	Multiplier to calculate the additional standard deviation in BCR affinity distribution after somatic hypermutation.	This parameter adjusts the standard deviation of the BCR affinity distribution of circulating B cells after each round of somatic hypermutation (Fig. 1, step 5) .<br><br>If τ^c is too high: The affinity distribution of B cells flattens, increasing the probability of extremely high or low affinity B cells. This reduces the proportion of cells falling within the selection thresholds, resulting in fewer B cells differentiating into plasma cells or continuing circulation.<br><br>If τ^c is too low: The B cell population may lack any clones within the plasma cell differentiation range (between $th_{high}\delta_i$ and $th_{ang}\delta_i$, Fig. 1, step 4) or circulation range (between $th_{apop}\delta_i$ and $th_{high}\delta_i$, Fig. 1). This outcome is biologically implausible since the selection pressure imposed by the T cells in the GCs is competitive and operates on a relative basis, resembling a rank-based selection process.<br><br>Therefore, this parameter requires careful calibration based on either:<br><br>1. Known plasma cell numbers and affinities from experimental data, or<br>2. Known taxonomic abundances, by observing the feedback loop between IgA and microbial populations (as done in our calibration process).

2. More importantly, as a modeler myself, the first criticism of any model by experimentalists is their lack of trust in results generated by models they lack the training to understand. This is true unless these models provide predictions that can be translated into, hopefully, simple experiments to test and validate them. All of the findings reported in this paper either recapitulate published data or provide predictions that have not been validated as part of this study and may be very difficult to validate overall. This is, in part, a limitation of trying to model any complex system. However, if the model cannot provide testable predictions that can either refute it or validate it, it is very hard to judge its scientific merit and contribution. In a model with so many parameters, fit to relatively small datasets (albeit ones that are probably current state-of-the-art), it is very easy to overfit the model. This, in my mind, is the major weakness of the paper. I would, therefore, urge the authors to either point to specific predictions provided by the model that are not currently known — and possibly to suggest feasible experiments that can be carried out to test these predictions. Ideally, at least one such prediction would be validated as part of the current study. Even if this is not the case, the paper needs to clearly outline findings that are not currently proven and why and to what extent they matter.

We approached the important challenge of validation in three ways: first, we tried to improve our manuscript by clearly communicating that the primary scope and merit of this work is to lay the foundations of a mathematical modeling framework that synthesizes the extensive qualitative and quantitative knowledge from existing literature; second, we include a new experimental model validation section that directly engages with ways of probing model predictions to assess model validity; and finally, we extended the work to include one test of our model via existing external data. For the latter, we extensively reviewed the literature to try and locate suitable

datasets to probe our model predictions; this was the only one that emerged. It would be fantastic to extend our manuscript with further such investigations of model validity; however, we possess neither the resources nor the expertise to do so. However, we hope that this analysis will inspire others to produce results designed in ways that will inform the core principles of immune education. Point by point, with references to the new elements:

1. Clearly articulating the novel predictions of our model in the discussion section and their clinical relevance.
2. Incorporating an additional analysis for external validation using the cohort data provided in Olivares *et al.* (2018), as explained below (Supplementary material, section "Illustrative validation using an external dataset", lines 2122-2161 and lines 652-654 in the revised main text). This analysis serves as an illustrative example of how model predictions in Fig. 3E can be externally evaluated by aligning them with real-world data.

   "Illustrative validation using an external dataset

   To assess the validity of our model outputs, we compared them against an independent, external dataset. This analysis serves as an illustrative example of how model predictions in Fig. 3E can be externally evaluated by aligning them with existing data not used in model development. We examined the predictive power of microbial phyla and calprotectin levels in distinguishing celiac disease (CD, 2 subjects) from healthy controls (HC, 4 subjects) in infant gut microbiomes based on the data published by Olivares et al. (2018)*. We filtered subjects that were delivered vaginally, had no antibiotic exposure, and were breastfed until month 4, and used their samples collected at 6 months of age as predictors of disease state. Logistic regression models were used for each individual predictor (Abundances of Proteobacteria, Actinobacteria, Bacteroidetes, Firmicutes, and levels of Calprotectin), employing a cross-validation strategy with 3 folds and 100 repeats, while using upsampling to address class imbalance. Model performance was evaluated using Area Under the ROC Curve (AUC) with bootstrap-derived confidence intervals. Based on our model, we classified immunological outcomes as tolerant vs. not tolerant (affinities against all symbiotic commensals all below 1 simultaneously or not, respectively) and used this as a proxy for HC vs CD, respectively. We used 100 simulations where the breastfeeding duration randomly varied between 120 and 180 days to mimic the feeding patterns of the subjects in the study. To represent a measurement taken within month 6, we sampled the predictor values at random days between day 150 and day 180. To calculate the AUC values at each iteration, we subsampled 6 subjects with 2 subjects being hyperreactive and 4 subjects being tolerant to resemble the data distribution. To prevent overfitting, we excluded any bootstrap iterations that produced AUC values of 1 during model performance evaluation. We then used the summary statistics of these AUC values. Our model showed good agreement with experimental data in terms of taxonomic rankings (Fig. S11A), with no rank differences across all five predictors. Both model and data (Fig. S11B) consistently identified Calprotectin as the strongest predictor (AUC of 1.00 in data and 0.73 on average in the model), which is expected given CD's inflammatory nature. More significantly, our model accurately reproduced the relative predictor importance patterns observed in the data across all taxonomic groups, providing a source of external validation for our model outcomes.*

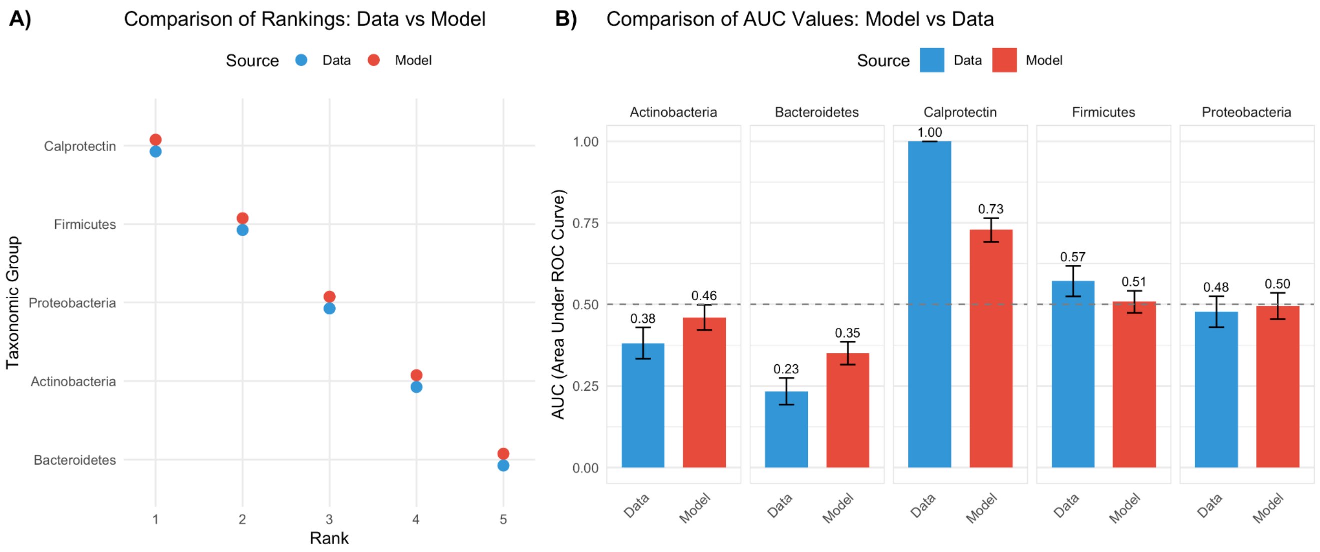

Fig S11. Comparison of predictive performance between cohort data and computational model. A) *Comparison of rankings for predictors between experimental data (blue) and model predictions (red). Connected points indicate the same predictor, with horizontal position showing rank order (1-5) in predictive importance. Identical ranking of paired points demonstrates the strong agreement between the model and the cohort data.* ***B)*** *Comparison of AUC (Area Under ROC Curve) values for cohort data (blue) and model predictions (red). Error bars represent the standard error. Calprotectin shows the highest predictive power in both datasets, while taxonomic groups demonstrate a moderate predictive performance. The dashed horizontal line at 0.5 represents the threshold for random prediction."*

3. Adding a new section to the supplementary material called "Experimental Model Validation" (old version renamed to "Experimental Model Parametrization" in the revised version) to provide plausible experiments and cohort studies that would either validate or reject the predictions of our model (lines 2334-2403), as provided below.

Experimental Model Validation

Our model is rooted in a mathematical formalization of well-described mechanisms, with the goal of predicting hard-to-intuit outcomes of emergent properties of mechanistic interactions. However, how the different mechanisms are combined in the model may lead to inappropriate weight to different processes, leading to mismatch between model predictions, and empirical outcomes, and this could be interrogated with simple experiments or cohort studies. We provide some plausible experiments below to test the predictions of our model.

1. Longitudinal cohort study to validate predictive power of fecal samples

Prediction: *Our model predicts that the predictive power of SIgA-bound Enterobacteriaceae abundance for downstream immune phenotypes follows a non-monotonic pattern over the course of ontogeny (Fig. 3E).*

Approach: *This can be tested in a longitudinal human birth cohort by collecting monthly fecal samples for 16S rRNA sequencing and IgA-seq from birth to two years of age, coupled with clinical follow-up for allergic or inflammatory outcomes. Machine learning models trained on different timepoints could assess how well microbial and SIgA-binding features at each stage predict immune outcomes. This would validate the model's proposed temporal window of maximal diagnostic value and inform optimal sampling strategies in clinical research.*

2. Probiotic supplementation as an intervention when breastfeeding is not possible

Prediction: *Our simulations indicate that in the absence of maternal antibodies, the early introduction of key symbiotic commensals belonging to Bacteroidaceae and Clostridiales can reduce the pathogenic-to-commensal ratio up to 30 fold in the gut lumen (Fig. 5A). This finding supports the use of targeted probiotic supplementation as a practical intervention when breastfeeding is not possible.*

Approach: *In mouse models, neonates receiving exclusive complementary feeding could be administered defined probiotic cocktails, with outcome measures including Enterobacteriaceae abundance in fecal samples and inflammatory biomarkers such as calprotectin. Based on our model predictions (Fig. 5A), we would expect a marked reduction in the pathogenic-to-commensal ratio — potentially up to 30-fold — along with a steadily decreasing trend during the first 30 days of life. While the precise magnitude may vary, reproducing this directional shift would lend support to the proposed mechanism and provide a foundation for future translational work. Although further validation in human settings is needed, this experimental design offers a feasible approach to test whether targeted early-life supplementation can reshape gut ecology in accordance with model predictions.*

3. Attenuation of hyperreactivity in offspring of mothers with IBD or allergies

Prediction: *Our model predicts that even when maternal SIgA displays hyperreactivity toward commensals—as may occur in mothers with IBD or allergic disorders—the immune system of the offspring does not simply replicate this reactivity, but exhibits an attenuated phenotype (Table 1, Fig. S4).*

Approach: *Murine models of maternal gut inflammation provide a tractable system to test this prediction. Dams with DSS-induced colitis—a condition known to alter intestinal SIgA reactivity—can be used to generate offspring exposed to hyperreactive maternal antibodies via breastfeeding. Fecal IgA-seq can be performed on both dams and their offspring across developmental timepoints to quantify the similarity of SIgA-binding profiles. Comparing the magnitude of hyperreactivity in maternal versus offspring SIgA responses will allow quantification of attenuation, which can be directly compared with model-predictions in Table 1. Complementary measurements of tolerogenic markers in the gut, such as regulatory T cell abundance and/or cytokine levels in the lamina propria, can further assess whether immune programming remains biased toward tolerance. Experimental confirmation of such attenuation would support the model's prediction that breastmilk, even from an immunologically dysregulated mother, retains regulatory properties that buffer against the vertical transmission of inflammatory phenotypes.*

4. Knockout experiments to test the significance of M cell sampling bias

Prediction: *Our model predicts that the bias in M cell-mediated antigen sampling — the preferential uptake of SIgA-coated bacteria over uncoated ones — is critical for driving the development of immune tolerance (Figs S6 and 6). This bias results in an overrepresentation of masked commensals in antigen presentation (since the neutralized ones will be expulsed from the lumen, they will not be sampled), promoting tolerogenic programming of dendritic cells, particularly via the C-type lectin receptor SIGNR1 [75] and regulatory follicular T cell responses. If this sampling preference is lost, the tolerogenic bias of the GC reactions will also be lost, increasing the risk of hyperreactivity.*

Approach: *While the selective adherence of M cells to SIgA is functionally well-established [73,74], the molecular identity of the responsible receptor remains unknown, making direct genetic knockout experiments unfeasible. However, it is not the sampling bias per se but its downstream effect — the conditioning of dendritic cells in the subepithelial dome — that ultimately drives tolerance. Therefore, our prediction can be tested using SIGNR1 knockout (Signr1–/–) mice, where DCs lack the receptor necessary for tolerogenic response to SIgA [165]. Neonatal mice can be colonized with a defined microbial consortium and provided maternal SIgA via breastfeeding. Comparing fecal IgA-seq profiles from Signr1–/– mice and wild-type controls, one can assess whether the absence of SIGNR1 disrupts the expected development of masking SIgA profiles and tolerance.*

3. The figures in the paper — including the model overview — were hard to follow. Legends need to be more detailed, and additional effort should be made to make these findings accessible to the non-technical reader. For example, even though I am fully versed in feature importance measures, I was unclear as to how the directionality of the weights is determined and what the dotted line was — perhaps an average, but that does not make sense on panel B. Similarly, what is the shaded area in Figure 5A? Do these predicted abundances actually align with data from humans? There have been a few good studies on sIgA-deficient infants — is the increase in Enterobacteriaceae over time reported?

We have revised all the figures and their legends (especially Fig. 1) in the main text to improve clarity and accessibility for both technical and non-technical readers. Regarding the specific points raised:

1. **Feature importance directionality:** In the original figure, the direction of the weights reflected the correlation between the predictors and the outcome, as noted in the figure legend: *"The directionality of*

the importance values is determined by the correlation between the outcome variable and the predictors". However, we agree this added unnecessary complexity to the interpretation of predictor importance. We have revised this figure to display only the magnitude of importance.

2. **Dotted lines in Panel B:** The dotted lines represented 50% importance thresholds, intended as reference points for comparison. Upon reconsideration, we agree these were potentially confusing, and removed them in the revised version.
3. **Shaded area in Figure 5A:** This shading indicated the region where the pathogenic/symbiotic commensal ratio fell below 1. We recognize this was redundant and removed it in the revised version.

Regarding alignment with SIgA deficiency data: To clarify, Figure 5 explores scenarios related to maternal (not infant) SIgA deficiency. Unfortunately, there is currently no publicly available data to our knowledge that directly aligns with our model predictions in this context. Most existing literature focuses on preterm infants with necrotizing enterocolitis (NEC) susceptibility, which provides qualitative supporting evidence for our findings. For example, NEC is associated with increased *Enterobacteriaceae* abundance in the gut microbiome, consistent with our model's predictions of elevated *Enterobacteriaceae* levels under maternal SIgA deficiency. Gopalakrishna *et al.* [60] demonstrated that IgA is critical for preventing NEC in a mouse model, with pups reared by IgA-deficient mothers showing increased disease susceptibility **despite exposure to maternal milk.** Additionally, multiple studies have shown correlations between reduced breastmilk IgA levels and inflammatory conditions in offspring, as mentioned in our results section. However, these studies cannot be used for direct quantitative validation of our model outputs, as they lack the necessary longitudinal data on infant gut microbiome composition and breastmilk immunological profiles. This highlights a significant gap in the current literature that our modeling approach aims to identify, underscoring the need for comprehensive longitudinal studies that capture both maternal sIgA profiles and infant microbiome development.

4. A major assumption made in the paper is that sIgA can be neutralizing or masking — and this distinction on its own is not very clear to me. Any bacteria that are coated by antibodies are not able to properly interact with the gut lumen and other bacteria. If the only difference between the N and M antibodies is affinity, this is a somewhat strange classification to make, as affinity is a highly continuous measure, and antibodies often have partial neutralization capacity (quantified by the neutralization titer, for example).

Thank you for this insightful comment. We agree that masking and neutralizing functions should not be mutually exclusive, and antibodies may simultaneously exert both functions – albeit at different rates – depending on their affinity. We have revised our model removing the assumption that these functions are mutually exclusive.

In the revised model, antibodies can both mask and neutralize simultaneously with different rates based on their affinity levels (Fig. 2B). We have completely re-fitted the model and performed all analyses from scratch with this improvement. Although this change does not substantially impact most simulated scenarios (since the majority of feeding patterns lead to tolerance, resulting in very low endogenous SIgA affinities for symbiotic commensals and very high affinities for pathogenic taxa, leading to dominantly masking and dominantly neutralizing antibodies with small rates of the opposite function respectively), it does affect intermediate cases, in ways that we do feel significantly improve our results. The key revisions resulting from this change include:

1. **Fig. 2B:** Revised to show that SIgA function rates include both masking and neutralizing functionalities for all taxa.
2. **Fig. 3A:** Revised to reflect the continuous gradient of masking rates rather than binary outcomes of tolerance versus hyperreactivity, since affinity values and their actions are now continuous measures.
3. **Fig. 3B:** Similarly revised to demonstrate both masking and neutralizing capacities of all antibodies across the affinity spectrum.
4. **Fig. 6 and Table 1:** Added to better explore the changes in our analysis from Fig. 5, particularly for the "Hyperreactive SIgA in breastmilk" scenario, after implementing the partial capacity changes suggested by the reviewer.

We appreciate the reviewer's comment that we feel has considerably enhanced the biological plausibility of our manuscript and led to interesting additional insights such as the phenomenon of attenuated hyperreactivity explored in Fig. 6 and Table 1 in the revised version.

Another important point completely ignored by the model is the existence of memory phenotypes in the gut that may reflect early exposure to specific commensals, which may no longer reside in the gut. Relying solely on IgA-seq data misses any antibody specificities that may have developed earlier to pathogenic or commensal bacteria that are no longer present in the gut. Some of these unbound sIgA antibodies may also be a product of overproduction of antibodies targeted against existing bacteria, which may indicate recent dysbiosis.

We appreciate the reviewer's insightful comment regarding memory phenotypes that may reflect early exposure to specific commensals no longer present in the gut. This is indeed a crucial point – we had initially incorporated it indirectly into our model (formally unpacked in Supplementary Material, 'Modeling the role of Germinal Centres'), and discussed it. In the revised version, we further discuss in the Supplementary Material ('Memory B cell compartment' section of our model limitations) that while our model does not explicitly represent memory B cells as a separate compartment, we do account for immunological memory mathematically, which helps maintain a stable plasma cell population even in the absence of the stimulating bacterial antigen (lines 1541-1553). Specifically, we model this by setting the death rate of plasma cells to approach zero as the host matures, which effectively mimics the continuous replenishment of dying plasma cells by memory cells. This approach allows our model to maintain antibody specificities against bacteria that were encountered early in development but may no longer be present in the gut microbiota during later stages.

To explore the reviewer's comment and further ascertain whether this element of the model demonstrates the required capability, we conducted additional simulations where we implemented an extinction rate for *Bifidobacteriaceae* starting from day 120 and day 720 of life (two separate scenarios). As shown in the figure below, even when exposure to *Bifidobacteriaceae* is limited to only the first 3 months of life, our model maintains a consistent level of endogenous SIgA response against this bacterium for the next 10 years of simulation. This persistent response in the absence of the bacterial stimulus precisely captures the immunological memory phenomenon that the reviewer mentioned above. This aspect of our model ensures that we do not rely solely on **current** IgA-seq data, but also account for historically developed antibody specificities against bacteria that may no longer reside in the gut - addressing the reviewer's first concern regarding memory.

While we've addressed this point both mathematically in the Methods section and verbally in our Model Limitations section, we are willing to include the additional analysis below in the supplementary material if the reviewer considers it essential for clarity. Given the current length of our supplementary material, we opted not to include it in this revision.

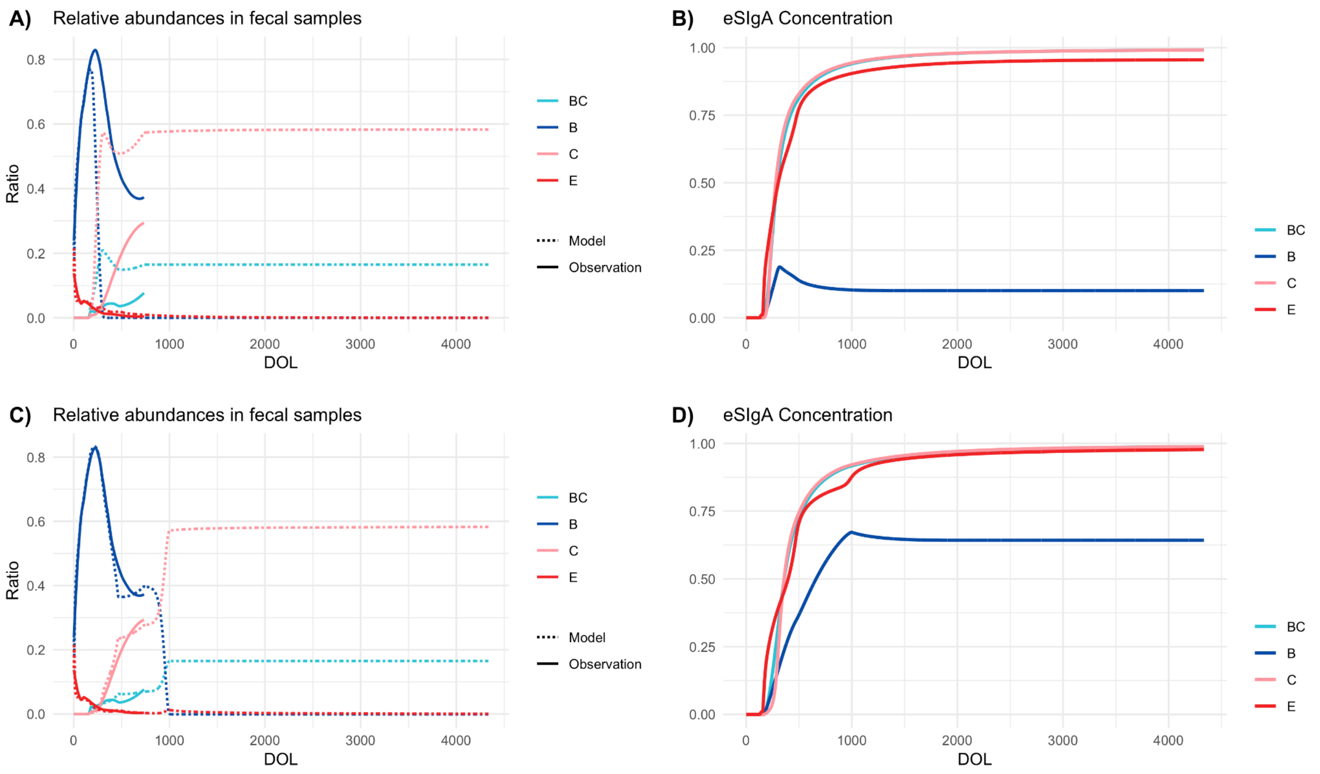

Fig R1. Demonstration of B cell memory by extinction simulations. Model outcomes simulating the gradual decline of *Bifidobacteriaceae* after **A-B)** DOL 120 and **C-D)** DOL 720 until its extinction. In our model, plasma cell death rate approaches zero as the host matures, mimicking T-cell-dependent cell longevity. Following *Bifidobacteriaceae* extinction, some plasma cells die due to lack of antigenic stimulation. However, cells that have received sufficient T cell help during later stages of maturation maintain eSIgA secretion at constant levels (panels **B** and **D**), effectively mimicking the continuous replenishment of dying plasma cells by memory cells.

5. The role of early life infections is completely ignored in the model, and how they may impact the development of the sIgA repertoire. Some infants suffer greatly from dysbiosis early in life, while others do not, and this will certainly also play a major role in shaping their antibody repertoire at two years of age.

Early life dysbiosis indeed plays a critical role in shaping the immune repertoire later in life (Belkaid *et al.*, 2015), which can also be recapitulated by our model. Although our original submission focused primarily on nutrition, the model is capable of simulating dysbiosis scenarios. To demonstrate this, we simulated early life infection by giving a selective advantage to the *Escherichia-Shigella* genus (compartment E in our model) relative to *Bifidobacteriaceae* by reducing the human milk oligosaccharides (HMOs) to 25% of their normal concentration in breastmilk while keeping all other parameters constant (maternal SIgA levels and affinities, breastfeeding duration, and mixed feeding periods).

As illustrated in the figure below, early life dysbiosis (overgrowth of *Escherichia-Shigella* compared to *Bifidobacteriaceae*, highlighted in the magenta circle) leads to more hyperreactive response against all commensals (highlighted in the magenta rectangle), but especially against *Bifidobacteriaceae* (compared to baseline) as they are most severely affected by the inflammation resulting from *Escherichia-Shigella* overgrowth. However, we also observe a dominantly hyperreactive response against *Bacteroidaceae* and *Clostridiales* (eSIgA affinity converging to levels above 1), since the immune response against these commensals are also impacted by the sustained inflammation initiated during the early dysbiosis, in line with our predictor importance analysis in Fig. 4.

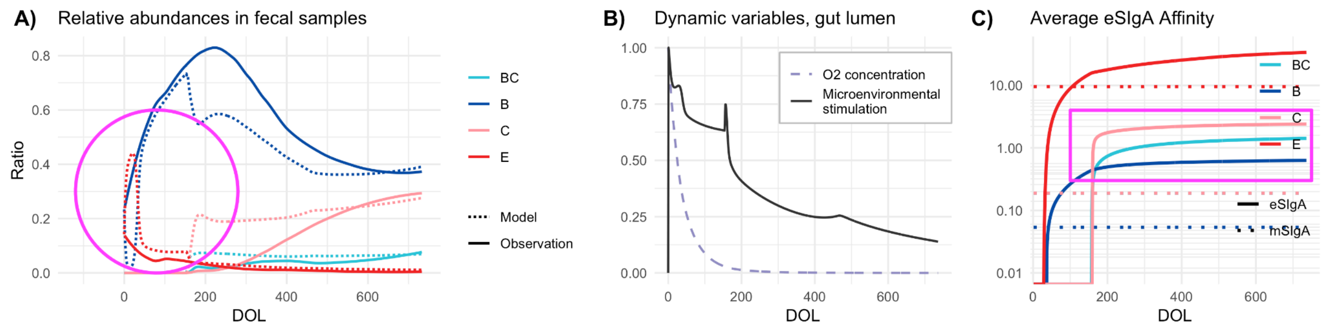

Fig. R2: Effects of early life dysbiosis on microbiome composition and immune response dynamics. Simulation of an early-life infection scenario by giving a selective advantage to the *Escherichia-Shigella* genus (compartment E) relative to *Bifidobacteriaceae* (compartment B) by reducing the human milk oligosaccharides (HMOs) to 25% of their normal concentration in breastmilk while keeping all other parameters constant (maternal SIgA levels and affinities, breastfeeding duration, and mixed feeding periods). Panel **A)** demonstrates the early life dysbiosis characterized by the overgrowth of E compared to data (magenta circle), which results in higher levels of sustained inflammation during early life (panel **B)**), leading to a more hyperreactive response against symbiotic commensals (magenta rectangle, panel **C)**).

Similar to our previous response, while we are completely willing to incorporate the additional analysis and figures into the supplementary material if deemed essential for clarity, we chose not to include them in this revision due to the current length of the supplementary materials. However, should the reviewer consider these additions necessary, we would be happy to provide them in the next revision.

Reviewer #2 (Matthew Olm):

In this manuscript, Tepekule et al. develop a model to explain IgA dynamics in the early microbiome, integrating both theoretical and experimental insights. They use this model to infer how various fecal samples could predict immunological trajectories.

Overall, I found this to be an interesting and valuable contribution to the field. I was particularly impressed by the authors' comprehensive review and synthesis of existing literature, which provides a strong foundation for their modeling approach. As someone outside the field of modeling, I found some aspects of the modeling challenging to follow, and I have concerns regarding certain assumptions and their potential impact on the results. However, this work offers a much-needed framework for understanding the role of IgA in infancy and has the potential to guide future research in this area.

Sincerely,
Dr. Matthew Olm
CU Boulder

Specific Comments:

1. I am particularly interested in how the input data were collated and formatted for the models. IgA-Seq data can be analyzed in multiple ways, and it would be helpful to clarify whether the authors processed raw data (e.g. sequencing reads themselves) or relied on previously published data in the form of supplemental tables.

2. Would it be feasible to provide all parsed and formatted input data for the model as a supplemental table? This would improve transparency and reproducibility.

We agree with the reviewer that data transparency and reproducibility is essential. To address points 1 and 2: We relied primarily on previously published data from supplementary tables, and where necessary, extracted quantitative data from figures in the original publications. In the previous manuscript version, we shared the

relative abundance data from Planer *et al.* and Tsukuda *et al.*, including our R script for pre-processing (Supplementary materials, "Data Curation and Pre-processing").

Upon the reviewer's recommendation, we have further improved transparency by curating and formatting all datasets used throughout our inference process, including both the relative abundance data, IgA-Seq data, and relative abundance data in the absence of breastfeeding. These comprehensive data files are now available in our repository at https://github.com/burcutepekule/ONIMTOL/. Each subfolder is named by the lead author of the corresponding reference (./TSUKUDA_DATA for relative abundance data, ./PLANER_DATA for IgA-Seq data, and PAN_DATA for relative abundance data in the absence of breastfeeding) including the data as Excel spreadsheets under the respective folders with R scripts we used to pre-process the data prior to inference (./DATA_TSUKUDA.R, ./DATA_PLANER.R., and ./DATA_PAN.R). We have revised the README.md of the repository and the corresponding sections of the supplementary materials accordingly.

3. The manuscript does not appear to discuss how formula-fed infants (who lack maternal IgA input) fit into this modeling framework.

Thank you for raising this important point, which we indeed addressed in our study but failed to communicate clearly in the manuscript. Our analysis included two distinct scenarios for the relative abundances during model fitting:

1. A baseline scenario with 154 days of exclusive breastfeeding followed by 308 days of mixed feeding, using high-resolution temporal relative abundance data from Tsukuda et al.

2. Cross-sectional data from Pan et al. providing relative abundance values for exclusively formula-fed children (as shown in Fig 2A of Pan et al.).

During inference, simulations mimicking the scenario in Pan *et al.* did not have any maternal IgA input as the reviewer pointed out. While this methodology was described in the Supplementary Material's "Data Curation and Pre-processing" section, we recognize this was not sufficiently clear and was omitted from Fig. 1A. To address this, we have revised Fig. 1A to acknowledge the data contribution from Pan *et al*. and have expanded our "Data Curation and Pre-processing" section to improve clarity as follows:

Lines 1737-1750: *"We used two datasets based on relative abundance data derived from stool samples during inference. First dataset is the 16S rRNA sequencing analysis combined with feeding metadata presented in Tsukuda et al. (20), …"*

Lines 1794-1804: *"The second dataset by Pan et al. [22] consists of 16S rRNA sequencing data from infants categorized by feeding type: breastfed, partially breastfed, and formula-fed. Due to the lack of detailed information on the partially breastfed group, we focus on the cross-sectional abundance data from formula-fed infants to impose additional constraints on the inference procedure. Relative abundances of E (~32%), B (~21%), BC (~23%), and C (~12%) provided in [22] for formula fed infants at day 30 of life (https://github.com/burcutepekule/ONIMTOL/blob/main/PAN_DATA/Figure2a_quantified.png) are scaled according to the total relative abundance of E+B+BC+C (72%) at day 29 provided in (20) and incorporated into the composite likelihood function representing the scenario of no breastfeeding. Including this scenario provides valuable information to the inference process as to how different calorie sources (HMOs and PDPs) impact the growth rate of different taxa."*

4. The analysis presented in Figure 3E would benefit from additional details, including the specific studies used for the predictions, the number of samples included in the analysis, and a clearer explanation of what exactly is being predicted. Additionally, including a version of this figure with data points representing the ground-truth data would provide better context for evaluating the model's predictive performance.

We agree that Figure 3E needed additional clarification. We revised the main text (lines 306-321) to better explain the analysis as follows. We want to clarify that the figure presents results derived from synthetic data generated by our model, a point we have now explicitly stated in the revised manuscript.

"Using this synthetically generated data, we explored the diagnostic potential of early life fecal microbiome data in predicting emergent immunopathology. Specifically, we analyzed how effectively the total and SIgA-bound Enterobacteriaceae abundance from fecal samples collected at various developmental stages could discriminate between individuals who ultimately developed tolerance versus hyperreactivity after 2 years (at DOL 735). We focused on SIgA-bound Enterobacteriaceae as our primary marker based on the evidence that SIgA coating patterns can identify bacteria associated with immune dysregulation and inflammatory conditions [43]. We first compared the total and SIgA-bound Enterobacteriaceae abundance between tolerant and hyperreactive subjects at different time points throughout development. Higher levels of SIgA-bound Enterobacteriaceae abundance gradually flip from reflecting tolerant to reflecting hyperreactive immunological states over the time course of ontogeny (Fig. 3D). Lower SIgA-bound Enterobacteriaceae observed for hyperreactive phenotypes during early phases of ontogeny are indicative of insufficient Enterobacteriaceae neutralization by maternal antibodies. As the host matures, this pattern alters, with the hyperreactive phenotype exhibiting an increase in SIgA-bound Enterobacteriaceae abundance relative to the tolerant state, echoing the enrichment in SIgA-coated Enterobacteriaceae in adults with IBD-like phenotypes [62,63]."

Regarding alignment with ground truth data: We were unable to find a publicly available dataset with sufficient temporal resolution to validate our results at each specific time point. However, we have conducted an additional analysis that compares the predictive power of taxa abundance and calprotectin levels in the cohort data provided in Olivares *et al.* (2018) with the predictive power of taxa abundance and microenvironmental stimulation in our model, as detailed below (Supplementary material, section "Illustrative validation using an external dataset", lines 2122-2161).

"Illustrative validation using an external dataset

To assess the validity of our model outputs, we compared them against an independent, external dataset. This analysis serves as an illustrative example of how model predictions in Fig. 3E can be externally evaluated by aligning them with real-world data trends. We examined the predictive power of microbial phyla and calprotectin levels in distinguishing celiac disease (CD, 2 subjects) from healthy controls (HC, 4 subjects) in infant gut microbiomes based on the data published by Olivares et al. (2018). We filtered subjects that were delivered vaginally, had no antibiotic exposure, and were breastfed until month 4, and used their samples collected at 6 months of age as predictors of disease state. Logistic regression models were used for each individual predictor (Abundances of Proteobacteria, Actinobacteria, Bacteroidetes, Firmicutes, and levels of Calprotectin), employing a cross-validation strategy with 3 folds and 100 repeats, while using upsampling to address class imbalance. Model performance was evaluated using Area Under the ROC Curve (AUC) with bootstrap-derived confidence intervals. Based on our model, we classified immunological outcomes as tolerant vs. not tolerant (affinities against all symbiotic commensals all below 1 simultaneously or not, respectively) and used this as a proxy for HC vs CD, respectively. We used 100 simulations where the breastfeeding duration randomly varied between 120 and 180 days to mimic the feeding patterns of the subjects. To represent a measurement taken within month 6, we sampled the predictor values at random days between day 150 and day 180. To calculate the AUC values at each iteration, we subsampled 6 subjects with 2 subjects being hyperreactive and 4 subjects being tolerant to resemble the data distribution. To prevent overfitting, we excluded any bootstrap iterations that produced AUC values of 1 during model performance evaluation. We then used the summary statistics of these AUC values. Our model showed good agreement with experimental data in terms of taxonomic rankings (Fig. S11A), with no rank differences across all five predictors. Both model and data (Fig. S11B) consistently identified Calprotectin as the strongest predictor (AUC of 1.00 in data and 0.73 on average in the model), which is expected given CD's inflammatory nature. More significantly, our model accurately reproduced the relative predictor importance patterns observed in the data across all taxonomic groups, providing a source of external validation for our model outcomes.

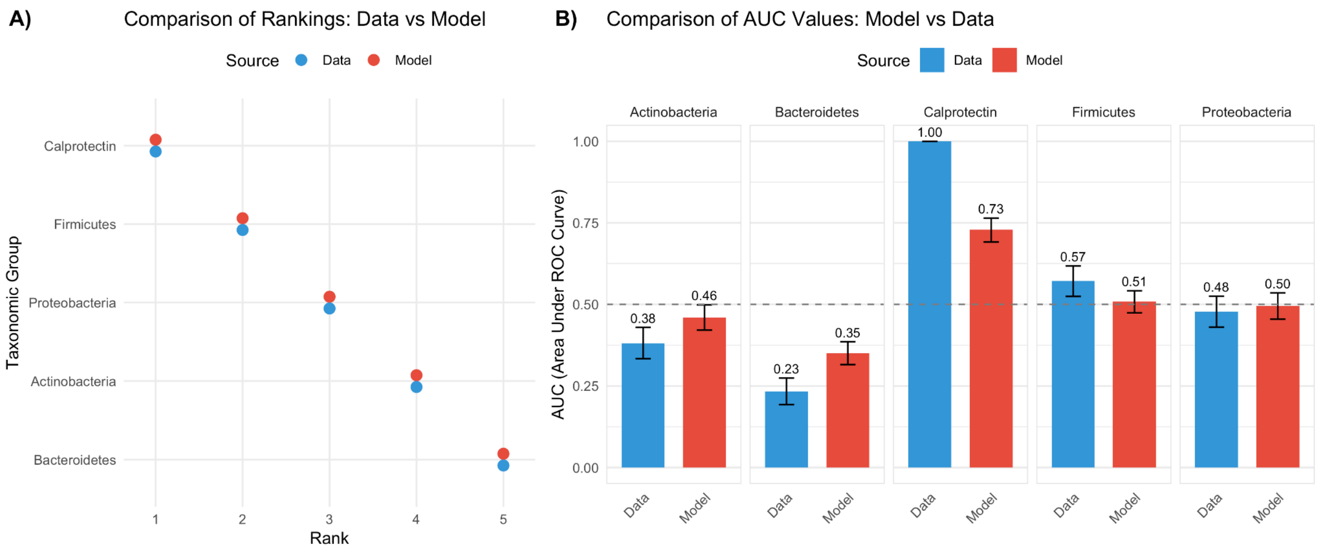

Fig S11. Comparison of predictive performance between cohort data and computational model. A) *Comparison of rankings for predictors between experimental data (blue) and model predictions (red). Connected points indicate the same predictor, with horizontal position showing rank order (1-5) in predictive importance. Identical ranking of paired points demonstrates the strong agreement between the model and the cohort data.* ***B)*** *Comparison of AUC (Area Under ROC Curve) values for cohort data (blue) and model predictions (red). Error bars represent the standard error. Calprotectin shows the highest predictive power in both datasets, while taxonomic groups demonstrate a moderate predictive performance. The dashed horizontal line at 0.5 represents the threshold for random prediction."*

Reviewer #3:

In the manuscript "The ontogeny of immune tolerance: a model of early life secretory IgA - gut microbiome interaction" the authors present a mechanistic mathematical model of postnatal microbiota development and immune maturation using existing microbiota and SIgA-16SrDNA sequencing data in combination with metadata e.g. on breastfeeding going beyond previous attempts (e.g. Planer et al., Nature, 2016). Aim is a quantitative parameter of postnatal immune maturation. Based on existing datasets from several independent studies and with several assumptions e.g. of dietary factors or luminal oxygen concentration they provide evidence that the inflammatory tone during the postnatal period determines postweaning mucosal immune maturation. The topic is timely and medically important given the association of early life events with life-long disease susceptibility. The reviewer recognizes the timeliness and effort and possible value of this study although future modifications may be needed to adjust the model. Some concerns remain that should be discussed/incorporated in the assessment.

Major points

1. The molecular structures recognized by SIgA are largely unknown although some (mainly peptide) structures have been recognized. The majority may actually recognize carbohydrate structures with major cross reactivity between bacterial taxa (and even phyla) generated independent of T cell help.

We agree with the reviewer that there has been accumulating evidence in the literature regarding T-independent IgA production due to direct B cell activation through carbohydrate structures, without the help of T cells and somatic hypermutation, thus more cross reactivity. However, two points of evidence led us to make the assumption that most IgA responses are T-dependent in humans. First, as mentioned in Table S4 (Additional assumptions implicit to the model structure), Human SIgA is highly somatically hypermutated (SHM), and SHM is commonly linked to T cell-dependent (TD) germinal center (GC) reactions (although not completely excluding T-cell independent (TI) mechanisms) (Gibbons *et al.*, 2011). Human Peyer's Patches, colonic patches, and isolated lymphoid follicles (ILFs) are organized by 22 weeks of gestation, which makes human infants able to

initiate GC reactions earlier and favor TD pathways even in infancy. Second, recent literature suggests that the TI/TD division is somewhat artificial in many contexts, as most microbes present as intact immunogenic particles containing both T-independent and T-dependent antigens, often in close proximity or even covalently linked (Bemark *et al.*, 2021). B cell receptors engaging with these particles may trigger uptake of the entire structure, leading to presentation of peptides derived from any protein within the microbe. This supports our modeling assumption that IgA-producing cells predominantly arise from TD germinal center reactions (Table S4, Additional assumptions implicit to the model structure).

Regarding cross-reactivity, this is something we haven't explicitly modeled in the current framework. While we acknowledge that cross-reactivity between bacterial taxa exists, as the reviewer correctly points out, the specific patterns and extent of this cross-reactivity remain largely unknown and difficult to quantify systematically. Pabst and Slack *et al.* (Mucosal Immunology, 2020) provide an extensive review of this topic, noting that "cross-species reactivity" of SIgA (binding to seemingly unrelated members of the microbiota) is a recognized phenomenon, but its mechanistic basis is still unclear. They state that cross-reactivity can occur through multiple mechanisms: shared epitopes between species, similar glycan motifs present on taxonomically distant bacteria, or a combination of canonical and noncanonical binding. However, without comprehensive empirical data on the specific molecular patterns shared between our taxonomic groups, the relative contribution of these shared patterns to overall IgA binding, and the quantitative strength of cross-reactive binding compared to species-specific binding, it would be challenging to implement a well-parameterized model of cross-reactivity. **Therefore, assuming that the antigen-specific responses are the main drivers of the ecological composition,** we chose to model IgA responses to each taxonomic group independently, in an antigen specific manner. This simplification allowed us to maintain model tractability while still capturing the primary dynamics of interest.

We fully agree that incorporating cross-reactivity would enhance model realism and should be discussed as a model limitation. Therefore, we added the following paragraph to our Model Limitations section (lines 2252-2263), and also mentioned cross-reactivity in our response to comment #3.

*"**Cross-reactivity of SIgA:** While we model antigen-specific SIgA responses independently across taxonomic groups, this does not account for evidence that SIgA can exhibit cross-species and even cross-phyla reactivity. Such cross-reactivity may arise from shared epitopes between species, similar glycan motifs present on taxonomically distant bacteria, or a combination of canonical and noncanonical binding (Pabst and Slack et al., 2020). These mechanisms allow individual IgA clones to bind to diverse microbial taxa, potentially blurring the boundaries of taxon-specific immune targeting. However, the mechanistic basis of this "cross-species reactivity" remains unclear (Pabst and Slack et al., 2020). Quantitative data on the range and specificity of SIgA binding across microbial communities are still limited, making it difficult to parameterize cross-reactive binding events in a mechanistically grounded way. While our antigen-specific modeling approach preserves model tractability and captures key dynamics of interest, it likely underestimates the masking effect of low-affinity SIgA binding across symbiotic commensals, representing an important avenue for future model refinement. "*

2. Although Peyer's patches are important for SIgA production, other pathways of immune activation (e.g. via GAPs and MLN) exist.

We thank the reviewer for highlighting that Peyer's patches (PPs) are not the sole site of immune activation in the gut, and that Goblet Cell-Associated Antigen Passages (GAPs) and mesenteric lymph nodes (MLNs) contribute to the complexity of mucosal immune responses.

GAPs play a crucial role in sampling luminal antigens and delivering them to lamina propria antigen-presenting cells during early life. This route is essential for establishing oral tolerance—particularly during the pre-weaning period—by promoting the imprinting of tolerogenic dendritic cells (DCs) and maintaining regulatory T cells (Tregs) (Kulkarni et al., 2019; McDole et al., 2012; Knoop et al., 2017). MLNs, as the downstream draining site of the imprinted DCs, serve as lymphoid organs where migratory dendritic cells can support IgA class switching and germinal center formation (Li et al., 2019). However, their role in microbiota-specific SIgA generation appears to be primarily modulatory. Both PPs and MLNs interact with dendritic cells and B cells, but PPs and

colonic patches are more directly involved in the initial antigen-specific response, whereas MLNs contribute to the broader regulation and dissemination of IgA-producing cells (Hashizume-Takizawa et al., 2019a; Hashizume-Takizawa et al., 2019b; Li et al., 2019). Hahn et al. (2010) demonstrated that MLN resection did not abrogate SIgA responses and, suggesting that MLNs are secondary to SIgA induction against enteric antigens.

Our model specifically focuses on the IgA responses to the gut microbiota during early life, a process that is most robustly supported by germinal center reactions within organized mucosal inductive sites—including PPs, colonic patches, and isolated lymphoid follicles. These structures provide direct access to luminal antigens through M cells, and have been shown to be indispensable for generating taxon-specific IgA against commensal bacteria (Rios et al., 2015).

To acknowledge the broader immunological context, we have added a paragraph to the model limitations section, noting that while our focus is on organized GALT structures and germinal center dynamics, we do not incorporate parallel routes such as the GAP–MLN axis that are relevant for early-life tolerance and systemic imprinting, as follows (lines 2279-2294, Model Limitations section in Supplementary materials):

*"**Alternative Pathways for Immune Imprinting:** Our model focuses on the induction of microbiota-specific IgA via germinal center dynamics within organized mucosal inductive sites, such as Peyer's patches and colonic patches. These structures directly sample luminal antigens through M cells, are known to support affinity maturation and class switching in response to microbial colonization, and have been shown to be indispensable for generating taxon-specific IgA against commensal bacteria []. We do not explicitly incorporate parallel routes of immune activation, such as the goblet cell-associated antigen passage (GAP)–mesenteric lymph node (MLN) axis. This pathway is particularly relevant during pre-weaning, when GAPs facilitate the delivery of luminal antigens to tolerogenic dendritic cells in the lamina propria, which then migrate to MLNs to support regulatory T cell expansion and systemic immune imprinting []. While MLNs are capable of supporting germinal center formation [], their role in microbiota-specific SIgA generation appears to be primarily modulatory. Both PPs and MLNs interact with dendritic cells and B cells, but PPs and colonic patches are more directly involved in the initiation of taxon-specific responses, whereas MLNs contribute to the broader regulation and dissemination of IgA-producing cells []. These mechanisms, while essential for establishing immune homeostasis, lie outside the specific scope of our current model."*

3. The human microbiota data have been mainly derived from fecal samples that by and large are representative for the colonic microbiota. SIgA, however, is mainly produced in the small intestine (where also the Payer's patches are located). Thus, the majority of SIgA-bound (colonic) bacteria may not indicate immune activation but reflect cross reactivity to common (e.g. carbohydrate) surface structures.

We thank the reviewer for highlighting the important distinction between the anatomical origin of fecal microbiota and that of SIgA production. In our initial framing, we focused on the small intestine and Peyer's patches as the primary site of SIgA induction, based on the well-established observation that SIgA titers and the proportion of IgA-coated bacteria are generally higher in the small intestine than in the colon.

In light of the reviewer's comment, we have clarified the anatomical interpretation underlying our model to more accurately reflect the nature of the data used—namely, fecal samples, which predominantly capture colonic microbial communities. Accordingly, our framework is more representative of a combined mucosal immune response, with a dominant contribution from colonic organized lymphoid tissues. While our anatomical emphasis has shifted, the structure of our model remains unchanged because the immunological processes we describe—specifically the germinal center dynamics involved in SIgA induction—are shared across both small-intestinal and colonic organized lymphoid tissues.

Colonic patches, like Peyer's patches, are organized inductive structures within the gut-associated lymphoid tissue (GALT). They exhibit key immunological features required for IgA induction, including M cell-containing follicle-associated epithelium, germinal centers with defined dark and light zones (as observed in canonical secondary lymphoid tissues and inducible mucosal lymphoid structures), high endothelial venules (HEVs), and segregated B and T cell areas. These similarities support our focus on the germinal center dynamics within

organized inductive sites of the gut-associated lymphoid tissue (GALT), without explicitly distinguishing between colonic and small-intestinal sites.

Moreover, although some IgA coating may result from cross-reactivity—where antibodies generated in Peyer's patches in response to local bacteria can bind structurally similar antigens on colonic bacteria—the high specificity of SIgA binding in fecal samples observed in the literature suggests that many of these bacteria are true immunogens capable of inducing local IgA responses in the colon. To acknowledge this nuance, we have revised Fig 1C, used the phrase *"organized gut-associated lymphoid tissue (GALT) inductive sites"* throughout the text rather than exclusively focusing on Peyer's patches, and revised the row on anatomical location in the implicit model assumptions table (Table S4) as follows (relevant citations are in the second column of Table S4, lines 1911-1912):

"Although SIgA titers and the proportion of IgA-coated bacteria are generally higher in the small intestine, our analysis is based on fecal sample data, which predominantly reflects the microbial composition of the colon. We therefore assume that the IgA responses observed in our data represent a combined mucosal immune response, with a dominant contribution from colonic organized lymphoid tissues. Although some of the IgA coating may result from cross-reactivity—where antibodies generated in Peyer's patches of the small intestine in response to small-intestinal bacteria bind to structurally similar antigens on colonic bacteria—the observed taxon specificity of IgA binding in fecal samples suggests that many of these bacteria are likely true immunogens, eliciting local IgA responses within gut-associated inductive sites in the colon.

Colonic patches, like Peyer's patches, are organized inductive structures within the gut-associated lymphoid tissue (GALT). They exhibit key immunological features required for IgA induction, including M cell-containing follicle-associated epithelium, germinal centers with defined dark and light zones (as observed in canonical secondary lymphoid tissues and inducible mucosal lymphoid structures), high endothelial venules (HEVs), and segregated B and T cell areas. Given this structural and functional similarity, our model focuses on the germinal center dynamics within such organized lymphoid tissues in the gut mucosa, without explicitly distinguishing between small-intestinal and colonic sites."

4. Breast milk, besides SIgA, HMO and dietary lipids and carbohydrates also contains EGF and TGFb influencing GAP activity and adaptive immune priming.

We appreciate the reviewer's observation. Our model accounts for the timing of M cell maturation by incorporating the role of maternal steroids, which regulate M cell development — a key determinant of antigen sampling at GALT inductive sites. While we do not explicitly model GAP activity, which is influenced by factors such as breastmilk EGF levels, our approach indirectly captures similar preweaning-associated transitions in antigen exposure by using maternal SIgA levels as a proxy for declining maternal steroids (Supplementary Materials, section *Informing time varying components of the model*, lines 1582-1596). Notably, EGF levels in breastmilk have been observed to decline around the time of weaning, similar to maternal steroids (e.g., Fig. 2 in Wagner *et al.*, 2021). However, since GAPs are not explicitly included in our model (see response to comment #2), we did not incorporate EGF concentrations directly. Nevertheless, we acknowledged how EGF levels in the breastmilk can influence the GAP activity in the revised version, where we discuss the GAP-MLN axis under Model Limitations section (Supplementary Materials, section *Alternative Pathways for Immune Imprinting*, lines 2279-2294).

Regarding TGF-β, we have acknowledged the role of maternally derived cytokines in shaping adaptive immune responses in the revised version (see response to comment #8 and lines 524-527 in the main text).

5. Mature M cells emerge at weaning in mice, the time point of their emergence in newborn humans is unknown but most likely earlier.

Indeed, while mature M cells are known to emerge around the time of weaning in mice, the timing of their maturation in human infants remains unclear, making precise parameterization challenging. However, this is exactly why we include a sensitivity analysis. Under the section *Local Sensitivity Analyses* in the supplementary

material, we explore a wide range of values for the timing of M cell maturation parameter (t^m), from day 30 to day 154 (time of weaning), to assess its potential impact on model outcomes. As seen in Fig Sxx and supported by our global sensitivity analysis, this parameter does not have a notable effect on the affinity maturation against symbiotic commensals. However, the pathogenic load in both the gut lumen and GALT inductive sites increases when M cells open earlier (a detailed explanation of this analysis can be found in section “Sensitivity to the time point of M cell maturation relative to the steroid levels in the breastmilk (parameter t^m), lines 2050-2055”). To acknowledge the uncertainty around the timing of M cell maturation in humans, we added the following sentence to the revised version: *“However, it is still not clear when exactly mature M cells emerge in human infants.”* before pointing to the sensitivity analysis (lines 2052-2053). We also revised the main text, emphasizing that this timing belongs to data from mice, and our sensitivity analysis addresses the ambiguity around the timing for humans (lines 131-137) as follows: *“M cells are shown to appear shortly before weaning in mice models, primarily due to decreasing concentrations of maternal steroids in breastmilk [35], for which we use mSIgA as a proxy, given a lack of quantitative data. While the timing of M cell maturation in humans remains unclear, we address this uncertainty through sensitivity analysis (Supplementary Materials, Local Sensitivity Analyses)."*

6. The authors claim that the inflammatory tone during the postnatal period determines postweaning mucosal immune maturation. Could the causality also be inverse and immune maturation cause the inflammatory tone?

We thank the reviewer for this insightful question regarding causality in our model. The reviewer correctly identifies an important consideration about the relationship between inflammatory tone and immune maturation. We acknowledge that our model incorporates a bidirectional relationship between inflammatory tone and mucosal immune responses. Inflammatory tone influences IgA production through dendritic cell conditioning and epithelial integrity, while IgA regulates microbial composition, which in turn affects inflammatory tone. This creates a cyclic relationship where cause and effect can be difficult to disentangle.

However, our predictor importance analysis in Figure 4B provides evidence for **temporal precedence** that helps establish directionality. By examining variables **at the start of weaning** (microenvironmental stimulation and pre-weaning commensal abundance) and their ability to predict eSIgA affinities against **post-weaning** taxa, we can observe which early factors most strongly influence later immune outcomes. This temporal sequence—where microenvironmental conditions during very early stages of life predict immune responses against bacteria that are introduced later to the gut—supports our interpretation that early inflammatory tone sets the stage for subsequent immune maturation. While immune maturation undoubtedly feeds back to influence inflammatory tone, our model suggests that the initial conditions established during exclusive breastfeeding have enduring effects that persist through the inflammatory tone.

We agree that this is an important point to address in the main text. We revised our results section by adding the following sentence (lines 421-427).

“While inflammatory tone and immune maturation undoubtedly have bidirectional interactions in our model, making causality difficult to disentangle, our analysis provides evidence for directionality through temporal precedence: early microenvironmental conditions at the start of weaning predict subsequent immune responses against taxa that only appear post-weaning. This temporal sequence helps untangle the cyclic relationship and supports the directionality from early inflammatory tone to downstream immune maturation.”

7. In the discussion, the authors propose that reducing the inflammatory potential e.g. of LPS from gram negative bacteria may promote tolerance. This is in contrast to work e.g. by Vatenen et al., Cell 2016 or Erika von Mutius showing a protective effect of early life endotoxin (or low abundance of TLR$ antagonistic pentaacylated Bacteroides LPS) from diabetes and asthma.

We thank the reviewer for this important point. Indeed, as highlighted by Vatanen *et al.*, not all LPS molecules are equal in immunogenicity. Our model focuses on hexa-acylated LPS from *Escherichia-Shigella*, which is known to be highly immunostimulatory. We propose that excessive stimulation from such LPS, particularly in the context of barrier integrity and/or concurrent infections, may contribute to the loss of tolerance. However, we

acknowledge that complete absence of immunogenic LPS — replaced by immunoinhibitory penta-acylated LPS (e.g., from *Bacteroides dorei*) — may also impair immune education, consistent with the findings of Vatanen *et al*. In fact, our model partially captures their findings: when there is significantly less hexa-acylated LPS from *Escherichia-Shigella* compared to baseline (artificially achieved by reducing the growth rate of *Escherichia-Shigella* by half), the immune system is *understimulated*, immune activation is inhibited, and our results show that immune education is impaired with zero to negligible affinity maturation against symbiotic commensals (Fig. R3). However, our model is primarily calibrated to explore scenarios of excess inflammation and breakdown of tolerance, rather than the nuances of how structurally distinct LPS molecules shape immune education. As such, we have not systematically analyzed alternative scenarios involving immunoinhibitory LPS. However, we agree that this is an important point to mention, therefore we have:

1. Revised the discussion section to make the importance of diverse LPS structures clear, as indicated in bold format below (lines 627-639):

"These findings, combined with the influence of ***excess*** *microenvironmental stimulation on endogenous SIgA affinity maturation suggests the possibility of alternative therapeutic approaches for promoting tolerogenic imprinting when maternal immunity is hyperreactive or insufficient. […] However, a complete exploration of such a scenario would necessitate expanding our model to explicitly incorporate cellular immunity,* ***and to account for the distinct immunological effects of structurally diverse LPS molecules on early-life immune education [78] (Supplementary material, section Model Limitations).****"*

2. Added a new section "Impact of LPS structures on immune education" in Model Limitations section in the revised version of the manuscript (lines 2265-2277), as follows,

"Impact of LPS structures on immune education: *Our model treats LPS as a generic inflammatory signal, primarily focusing on the immunostimulatory effects of hexa-acylated lipid A derived from Escherichia-Shigella. However, experimental evidence, notably from Vatanen et al. [78], demonstrates that LPS molecules vary significantly in their immunogenic properties depending on their acylation pattern and bacterial origin. Penta- and tetra-acylated LPS produced by Bacteroides dorei, for example, are antagonistic to TLR4 signaling and actively suppress immune activation. These immunoinhibitory LPS types not only fail to induce endotoxin tolerance, but can also block the immune-educational effects of stimulatory LPS, as shown in both in vitro assays and mouse models of autoimmunity. Because our current model does not distinguish between LPS subtypes, it cannot fully capture scenarios in which tolerance is impaired due to insufficient immune stimulation. Future iterations of the model could incorporate functionally distinct LPS categories to explore both ends of the immune education spectrum—from hyperactivation-induced breakdown to under-stimulation-induced failure."*

We are also happy to include the additional analysis in Fig. R3 in the supplementary material if the reviewer considers it essential for clarity. Given the current length of our supplementary material, we opted not to include it in this revision.

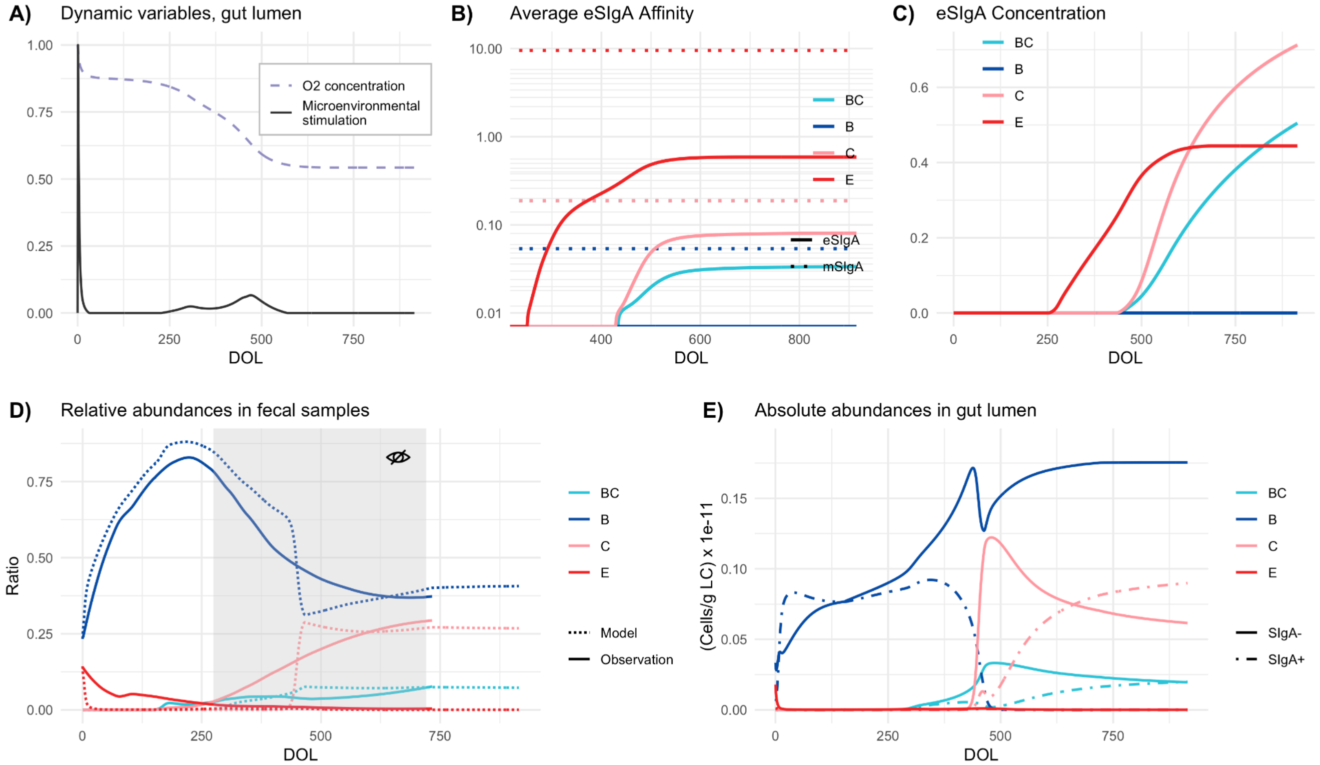

Fig. R3: Model-based exploration of immune responses in the absence of highly immunostimulatory LPS from *Enterobacteriaceae*. This figure illustrates how lack of microenvironmental stimulation driven by *Enterobacteriaceae* influences immune education and eSIgA affinity maturation. When there is significantly less hexa-acylated LPS from *Escherichia-Shigella* compared to baseline (artificially achieved by reducing the growth rate of *Escherichia-Shigella* by half, panel D), the immune system is *understimulated* (panel A), immune activation is inhibited, and immune education is impaired with zero to negligible affinity maturation against symbiotic commensals (panel B). **(A)** Normalized oxygen concentration and microenvironmental stimulation (normalized by its peak value, reflecting the inflammatory tone of the environment) over time. **(B)** Temporal progression of average endogenous SIgA (eSIgA) affinities (solid lines) gradually approaching the mSIgA affinities (dashed lines), **(C)** and the normalized antibody concentrations in the gut lumen for each key taxon. **(D)** Relative abundances in fecal samples aligned with observational data, where the gray area highlights the out-of-sample data during parameter inference. **(E)** Absolute abundances in the gut lumen for key taxa distinguished by coating status. Model fit estimates for all parameters with their respective description, unit, and prior distribution are provided in Table S3. DOL: Day of life; E: *Enterobacteriaceae*; B: *Bifidobacteriaceae*; BC: *Bacteroidaceae*; C: *Clostridiales*.

However, our model is primarily focused on the pathological consequences of hyperstimulation, and does not yet account for immunoinhibitory LPS subtypes such as those produced by Bacteroides dorei.

8. Published data suggest that mucosal immune priming may also be influenced by maternal cytokines (e.g. IL-6, Lim, Science, 2021) prior to birth. Please comment.

We agree with the reviewer that maternal cytokines can influence mucosal immune priming in the infant prior to birth. While our current model and analysis focus on SIgA-mediated effects via breastmilk, we acknowledge that maternal cytokines and other inflammatory mediators also contribute to shaping early immune development. Due to limited availability of quantitative data on their levels in breastmilk or their systemic transmission, we did not explicitly include them in our parameterization. However, their relevance is especially important in the context of Fig. 5, where we examine the attenuation of hyperreactivity across generations. To clarify this point for the reader, we have added the following sentence to the revised manuscript (lines 524-527):

"However, it is important to note that this result of attenuated transmission of hyperreactivity does not account for potential contributions from maternally derived cytokines (e.g., IL-1β, IL-6, TNF-α, TGF-β) or other mediators transferred through the placenta or breastmilk [76]"

Minor points:

* In Fig. 2E the authors normalize the antibody concentrations in the gut. Normalized to what?

In our formulation of the antibody concentrations, the values are intrinsically bounded between 0 and 1 due to the structure of our ODE system. Eqn (S1.2.2) ensures eSIgA remains bounded between 0 and 1 when number of plasma cells B_i^p stay below 1, which is an intrinsic constraint in our model to reflect the carrying capacity of plasma cells (Table S3). At eSIgA = 0, the derivative is positive (deSIgA/dt = $C_I B_i^p$ > 0), ensuring growth from zero. At eSIgA = 1, the derivative becomes $I_{hl}(B_i^p - 1)$, which is negative as long as $B_i^p < 1$, and zero when B_i^p=1, preventing values from exceeding 1. Therefore, S1.2.2 describes the change in the **fraction** of maximum possible antibody concentration, where 0 indicates complete absence and 1 indicates saturation. The normalization arises naturally from the mathematical formulation rather than from rescaling measured data. This approach allows us to focus on the relative dynamics of antibody behavior in the gut without being constrained by specific concentration units.

* In Fig. 2H the authors used the same dashed line for SIgA+ as in the Fig.2G which was there used for the model. This makes it hard to follow

We have revised Fig 2G and Fig 2H using different line types.

* In Fig. 4A/B the authors used Bacteroidaceae and Clostridiales as one group. For the sake of uniformity it would be good to distinguish them

We have revised Fig 4A and Fig 4B accordingly.

* In Fig.5 the authors used the same colors as in Fig.2 when illustrating the different taxa. I would advice using different colors or only working with one and the different line types

We have revised the colors in Fig. 5 and made sure they are distinct from Fig. 2.
