# Supplementary material for: A model of early-life interactions between the gut microbiome and adaptive immunity provides insights into the ontogeny of immune tolerance
Source: PLoS Biol. 2025 Aug 14;23(8):e3003263. doi: 10.1371/journal.pbio.3003263 (PMC12352683; doi:10.1371/journal.pbio.3003263)
Supplement: Attachment — Submitted filename: PLOS BIO RESPONSE TO REVIEWERS FINAL.docx [file pbio.3003263.s022.docx]

We would like to thank the PLOS Biology editors, the Academic Editor and two of the original reviewers for considering our manuscript for publication. We have addressed all the remaining points, and below we briefly summarize the key changes:

a) We routinely suggest changes to titles to ensure maximum accessibility for a broad, non-specialist readership, and to ensure they reflect the contents of the paper. In this case, we would suggest a minor edit to the title, as follows. Please ensure you change both the manuscript file and the online submission system, as they need to match for final acceptance:

"A model of early-life interactions between the gut microbiome and adaptive immunity provides insights into the ontogeny of immune tolerance"

Revised as suggested.

b) Could you please confirm that there is not grant number in some of the funding agencies?

Yes, we confirm that the grant of Ai Ing Lim **(AIL)** does not have a grant number. We are adding two other grants to the financial disclosure which were overlooked in the initial submission (grants added for **CJEM** also do not have grant numbers). We will provide the complete up-to-date funding information to Anitha Samidurai (publications assistant) as they also asked about the financial disclosure in the technical requirements.

c) Please note that per journal policy, the model system/species (human) studied should be clearly stated in the abstract of your manuscript.

Added to the abstract as suggested: *“[...]. We address this gap by synthesizing existing literature to develop a mechanistic mathematical model representing the interplay between gut ecology and adaptive immunity* ***in humans*** *during early life.”*

d) During the discussion one of the reviewers suggested that you put Fig R2 from your response to R1 point 5 in the supplementary material, as they found the analysis useful. We would like to encourage this.

This Figure has been added to the supplementary as Fig. S3., under the new section Role of Early Life Infections.

e) We do not have a word limit. Could you please move the section named Materials and Methods currently in the supplements in the main text? This would allow readers an easier access to the model. This also goes for the additional text and references placed there.

Revised as suggested.

f) Please supply the numerical values either in the a supplementary file or as a permanent DOI’d deposition for the following figures: Figure R2ABC, 2A-I, 3A-E, 4AB, 5A-C, 6A-D, S1ABC, S3AB, S4A-O, S5ABC, S6, S7, S9, S10, S11, Table 1.NOTE: the numerical data provided should include all replicates AND the way in which the plotted mean and errors were derived (it should not present only the mean/average values).

We have added all scripts that can regenerate all the Figures and Table 1 in the manuscript.

g) Please cite the location of the data clearly in all relevant main and supplementary Figure legends, e.g. “The data underlying this Figure can be found in S1 Data” or “The data underlying this Figure can be found in https://doi.org/10.5281/zenodo.XXXXX”

Revised as suggested:

*“The data underlying this Figure can be found in https://doi.org/10.5281/zenodo.15629746.”*

h) Please ensure that your Data Statement in the submission system accurately describes where your data can be found and is in final format, as it will be published as written there.

Revised as suggested:

*Data and materials availability: All data and code used in this work is available at https://doi.org/10.5281/zenodo.15629746 under the GNU General Public License v3.0.*

i) Many thanks for providing the underlying code in GitHub. However, because Github depositions can be readily changed or deleted, please make a permanent DOI’d copy (e.g. in Zenodo) and provide this URL in the manuscript and Data Availability Statement.

Please note that we cannot accept sole deposition of code in GitHub, as this could be changed after publication. However, you can archive this version of your publicly available GitHub code to Zenodo. Once you do this, it will generate a DOI number, which you will need to provide in the Data Accessibility Statement (you are welcome to also provide the GitHub access information). See the process for doing this here: https://docs.github.com/en/repositories/archiving-a-github-repository/referencing-and-citing-content

All points above related to the public repository has been updated with the corresponding permanent Zenodo DOI.

As you address these items, please take this last chance to review your reference list to ensure that it is complete and correct. If you have cited papers that have been retracted, please include the rationale for doing so in the manuscript text, or remove these references and replace them with relevant current references. Any changes to the reference list should be mentioned in the cover letter that accompanies your revised manuscript.

No changes have been made to the reference list.

*** Please note that some formulas are edited due to the discrepancy between the formatting between Google docs and Microsoft Word. Although these are tracked as revisions in the tracked version of the manuscript, none of the formulas have technically changed and are all identical with the previous version mathematically.

------------------------------------------------------------------------

REVIEWERS' COMMENTS:

------------------------------------------------------------------------

**Reviewer #3 (Mathias Hornef):** The authors have made a major effort and responded to all my points and the points raised by the other reviewers seriously, extensively and in very much detail. They also included changes in the manuscript where appropriate. It remains unclear to me how big the effect of potential presently neglected or unknown influencing factors might be for the final outcome of the model. But I do believe that it may be worth trying and subsequent adaptations might ultimately allow and lead to a suitable approach. I therefore would opt to recommend publication.

The only way I could think of further enhancing interest and attention to a wider audience could be some sort of one or two example inputs with calculation to illustrate the potential value and applicability for wet lab researchers in this area.

We completely agree with the reviewer's suggestion, as one of the primary aims of this study is to provide a quantitative tool that is accessible to the broader research community. To address this point, we have developed a user-friendly Shiny application (<https://burcutepekule.shinyapps.io/ONIMTOL/>) that allows researchers to easily manipulate model parameters and obtain model predictions through an intuitive interface. This tool eliminates the need for manual calculations and makes our modeling approach readily applicable for experimental researchers, regardless of their computational background. For more advanced users, our repository contains the scripts where the user can have full control over the model dynamics and parameters and can improve/manipulate it given their own experimental setup.
